# A green fabrication of pharmacologically relevant fused pyrimidines using recyclable caffeine–H_3_PO_4_ catalyst: insight into antibacterial and cytotoxic efficacy

**DOI:** 10.1039/d5ra10127a

**Published:** 2026-02-13

**Authors:** Ayushi Bhatnagar, Nitish Rai, Namita Ashish Singh, Vidhi Jain, Juhi Goyal, Gangotri Pemawat

**Affiliations:** a Department of Chemistry, University College of Science, Mohanlal Sukhadia University Udaipur Rajasthan India 313001 drgpemawat@mlsu.ac.in; b Department of Zoology, University of Lucknow Uttar Pradesh India 226 007; c Department of Microbiology, Mohanlal Sukhadia University Udaipur Rajasthan India 313001; d Department of Biotechnology, Mohanlal Sukhadia University Udaipur Rajasthan India 313001

## Abstract

An eco-friendly and high-yielding one-pot protocol has been developed for the synthesis of pyrimido[4,5-*d*]pyrimidine derivatives utilizing caffeine–H_3_PO_4_ as a recyclable, metal-free acidic catalyst under ethanolic conditions. This green approach delivers the desired fused heterocycles in notable yields within short reaction times, avoiding the use of harsh reagents and tedious purification steps. The catalyst was systematically characterized by FT-IR, NMR, XRD, SEM, EDX, TGA, DTG, DTA and DSC analyses, confirming its stability, robustness, and catalytic proficiency. Notably, caffeine–H_3_PO_4_ exhibited excellent recyclability across multiple runs with negligible reduction in activity. Eco-metric assessment further substantiated the environmentally benign nature of the process. Preliminary biological screening demonstrated significant cytotoxic potential, while computational studies including molecular docking, pharmacokinetic, and ADMET analyses supported the experimental outcomes and indicated favorable drug-like attributes. In summary, this work presents a green and practical synthetic route to biologically important pyrimido[4,5-*d*]pyrimidine frameworks, showcasing caffeine–H_3_PO_4_ as an effective and eco-compatible catalyst in modern heterocyclic chemistry.

## Abstract 2

An eco-friendly and high-yielding one-pot protocol has been developed for the synthesis of pyrimido[4,5-*d*]pyrimidine derivatives utilizing caffeine–H_3_PO_4_ as a recyclable, metal-free acidic catalyst under ethanolic conditions.

## Graphical abstract

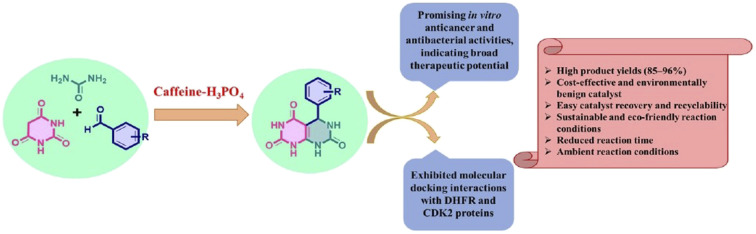

## Introduction

The chemistry of many medications, bioactive natural items, and functional materials is based on the crucial class of molecules known as organic heterocycles.^1–3^ They are essential to modern drug discovery and material innovation because of their capacity to participate in a variety of chemical transformations and create a range of interactions with biological systems.^4^ Because of their structural versatility and biological significance, pyrimidine and its derivatives have become key components of heterocyclic chemistry within this broad field.^5^ Basic biomolecules like cytosine, thymine, and uracil, essential parts of nucleic acids that support genetic information, are built around the pyrimidine ring.^6,7^ Pyrimidine-based compounds have shown a broad variety of multi-therapeutic potential, including antibacterial, anticancer, antiviral, and anti-inflammatory activities, in addition to their biological occurrence.^8^ In medicinal chemistry, this structural motif serves as a privileged pharmacophore that can bind precisely and selectively with biological receptors *via* electrostatic, π–π stacking, and hydrogen bonding.^9^ Their notable binding affinity arises from these characteristics, rendering pyrimidine frameworks attractive scaffolds for rational drug design. Reported studies indicate that pyrimidine derivatives commonly display binding affinities within the µM to nM range (approximately 10^−6^–10^−9^ M), depending on the biological target and the nature of structural modifications.^10,11^ Pyrimidine-containing drugs have also been explored for their pharmacokinetic behavior, particularly oral absorption and metabolic characteristics.^12–14^ For example, the orally administered prodrug Capecitabine (IC_50_ ≈ 235 µM) achieves higher systemic exposure relative to its active metabolite 5-fluorouracil (IC_50_ ≈ 39.81 µM), which requires *in vivo* conversion. Likewise, pyrimidine-based tyrosine kinase inhibitors such as Imatinib (IC_50_ ≈ 0.1 µM) exhibit high oral absorption, while others like Pazopanib (IC_50_ ≈ 0.03 µM) show comparatively moderate bioavailability.^15,16^ In addition, literature reports suggest that pyrimidine derivatives often possess adequate metabolic persistence, with microsomal half-lives typically exceeding 30–60 min or retaining more than 50–70% of the parent compound under standard assay conditions.^17,18^ Structural tunability of the pyrimidine core has further been associated with preferential interactions toward intended biological targets, with reported selectivity margins commonly falling in the 10–100-fold range, depending on substitution patterns and target class.^19,20^ Reported studies indicate that several pyrimidine-based compounds exhibit moderate stability in the presence of cytochrome P450 (CYP450) enzymes, as reflected by acceptable half-lives in human or rodent liver microsomal assays; however, the extent of CYP-mediated metabolism varies with substitution patterns and target class.^21^ Collectively, these literature observations highlight the versatility of pyrimidine scaffolds in supporting key parameters relevant to drug discovery.

Pyrimido[4,5-*d*]pyrimidine derivatives, which are made up of two condensed pyrimidine rings, have drawn a lot of attention among different fused heterocyclic systems due to their unique structure and wide range of therapeutic uses.^22,23^ These substances are potential prospects for therapeutic research due to their exceptional bioactivity, which includes receptor-specific binding, kinase regulation, and enzyme inhibition.^24,25^ Their fused structure's rigidity and conjugation promote molecular planarity and receptor complementarity, which enhances biological performance and selectivity.^26,27^ Several well-known medications, such Trimethoprim, a DHFR inhibitor with strong antibacterial activity (IC_50_ ≈ 198 µM against human DHFR); Pemetrexed, an antifolate chemotherapeutic agent (IC_50_ ≈ 6.6 µM against DHFR); Allopurinol, a xanthine oxidase inhibitor commonly used in the treatment of gout (IC_50_ ≈ 0.82 µM); and Minoxidil, an antihypertensive drug that also promotes hair growth (IC_50_ ≈ 20 µM in lipoxygenase inhibition assays).^28,29^ When taken as a whole, these examples highlight the crucial role that pyrimidine-based frameworks especially fused systems like pyrimido[4,5-*d*]pyrimidines play in contemporary synthetic and medicinal chemistry, acting as adaptable templates for the creation of novel therapeutic agents.^30,31^

**Fig. 1:**
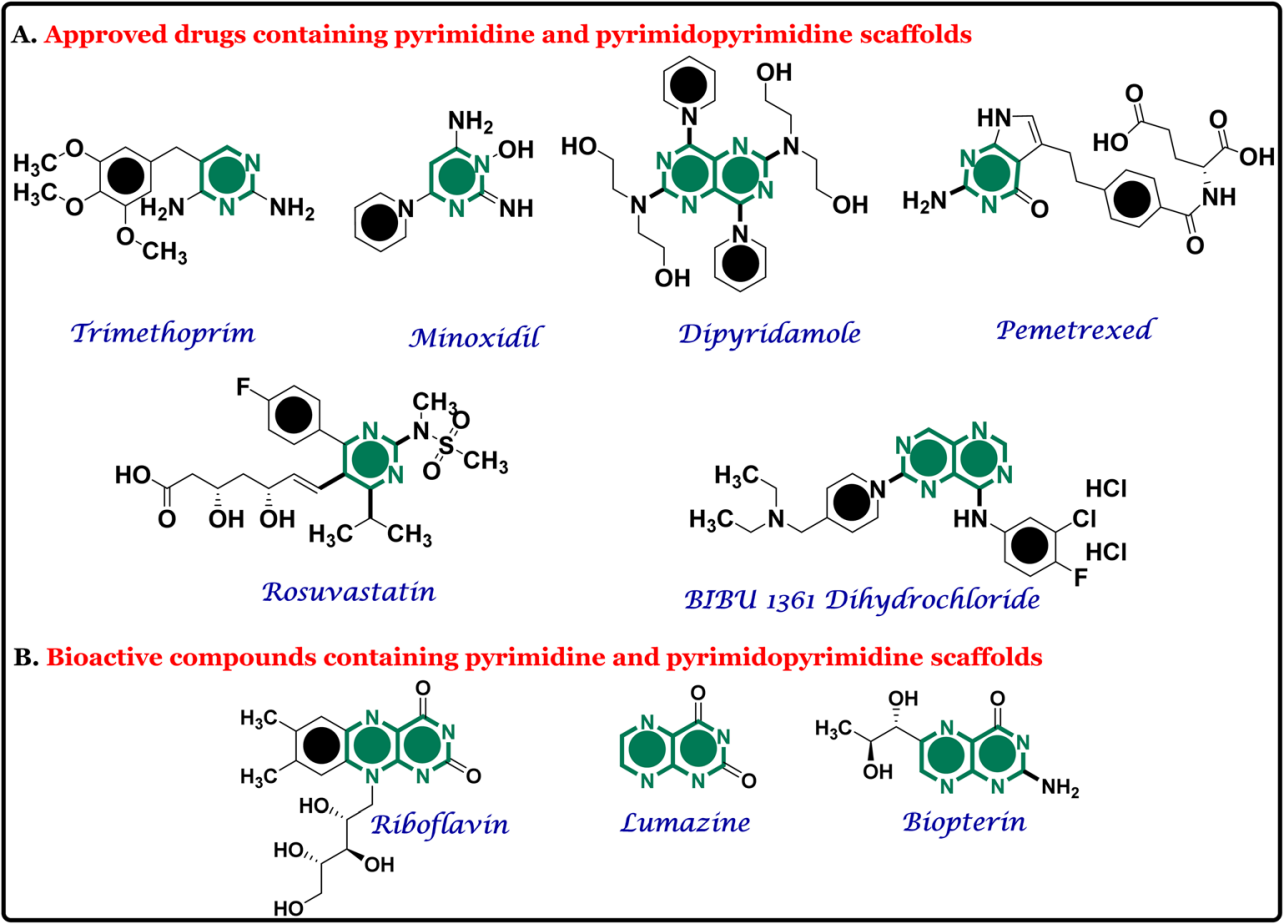
Representative examples of (A) approved drugs and (B) bioactive scaffolds containing pyrimidine and pyrimidopyrimidine scaffolds, illustrating the structural diversity and broad therapeutic relevance of these frameworks in modern medicinal chemistry.

These examples highlight the significance of pyrimidine and its fused analogs in drug design and clinical applications. Fig. 1 presents several approved drugs featuring the pyrimidine and pyrimidopyrimidine framework, highlighting its versatility in therapeutic applications.^32^

**Fig. 2:**
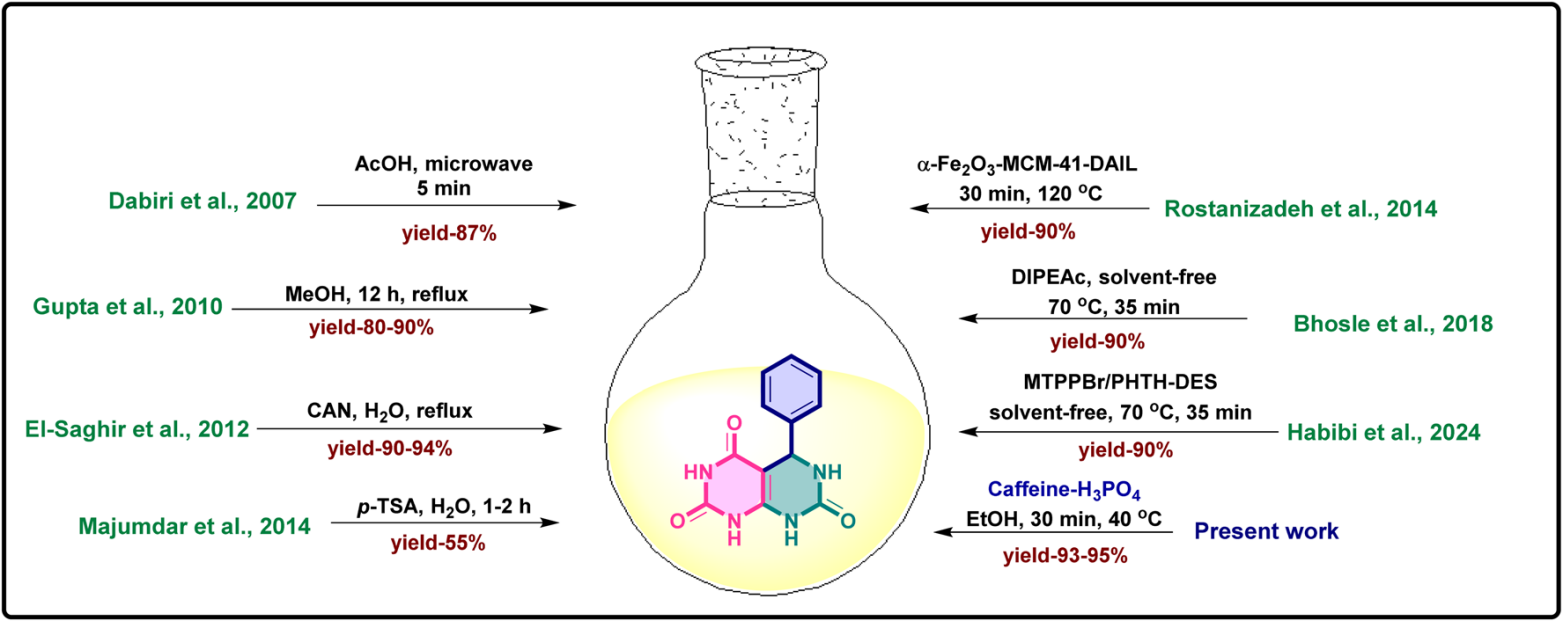
Comparative evaluation of the present caffeine–H_3_PO_4_ catalytic system with previously reported methods, highlighting improvements in reaction efficiency, yield, and eco-friendliness.

As a result, there is ongoing interest in creating effective and environmentally friendly processes for the synthesis of these molecules.^33^ A number of synthetic methods, such as condensation, cyclization, and multicomponent processes, have been documented over the years for the synthesis of pyrimido[4,5-*d*]pyrimidine derivatives.^34–36^ For these reactions, a variety of catalysts have been used, including metal-based catalysts, Lewis acids, mineral acids, and ionic liquids.^37^ Nevertheless, these techniques frequently have disadvantages including hazardous chemicals, lengthy reaction times, high reaction temperatures, and challenging catalyst recovery.^38,39^ The development of recyclable, metal-free, ecologically safe catalytic systems that can function effectively in mild settings has been aided by the growing emphasis on green chemistry in recent years.^40^ Caffeine, a naturally occurring xanthine alkaloid, has emerged as a safe, inexpensive, and biodegradable catalyst. Its molecular structure contains both carbonyl and nitrogen sites that can participate in hydrogen bonding and acid–base interactions, allowing it to facilitate a variety of organic transformations.^41,42^ Because of its availability, non-toxic nature, and mild catalytic behavior, caffeine has been explored for green organic synthesis involving condensation and multicomponent reactions.^43^ Phosphoric acid was employed in combination with caffeine to synergistically enhance catalytic efficiency under mild reaction conditions. This dual-acid system integrates the organic framework and stabilizing influence of caffeine with the Brønsted acidity of phosphoric acid, enabling effective proton donation and efficient activation of carbonyl functionalities.^44,45^ The presence of caffeine further improves catalyst stability, tunability, and ease of handling, while the operational simplicity and robustness of the system make it suitable for scalable synthesis. As part of our ongoing efforts to develop environmentally benign and efficient catalytic systems, we designed this caffeine–H_3_PO_4_ catalytic system as a recyclable, metal-free catalyst operating under mild ethanolic conditions.^46^ The protocol affords the desired pyrimido[4,5-*d*]pyrimidine derivatives in high yields (85–93%) within short reaction times (25–30 min), using a straightforward work-up procedure and enabling catalyst reusability over multiple cycles, thereby providing a sustainable and practical approach for the synthesis of physiologically relevant fused pyrimidine frameworks.^47,48^ Overall, this work provides an efficient and adaptable catalytic strategy that can be applied beyond pyrimidine frameworks, supporting greener synthetic methodologies and streamlined heterocycle construction.^49,50^ Its simplicity, environmentally conscious design, and wide-ranging applicability make it valuable for both academic research and potential industrial use.^51,52^ Pyrimidopyrimidine derivatives have been previously reported through various synthetic approaches, a few of which are mentioned below (Fig. 2).

## Results and discussion

**Fig. 3:**
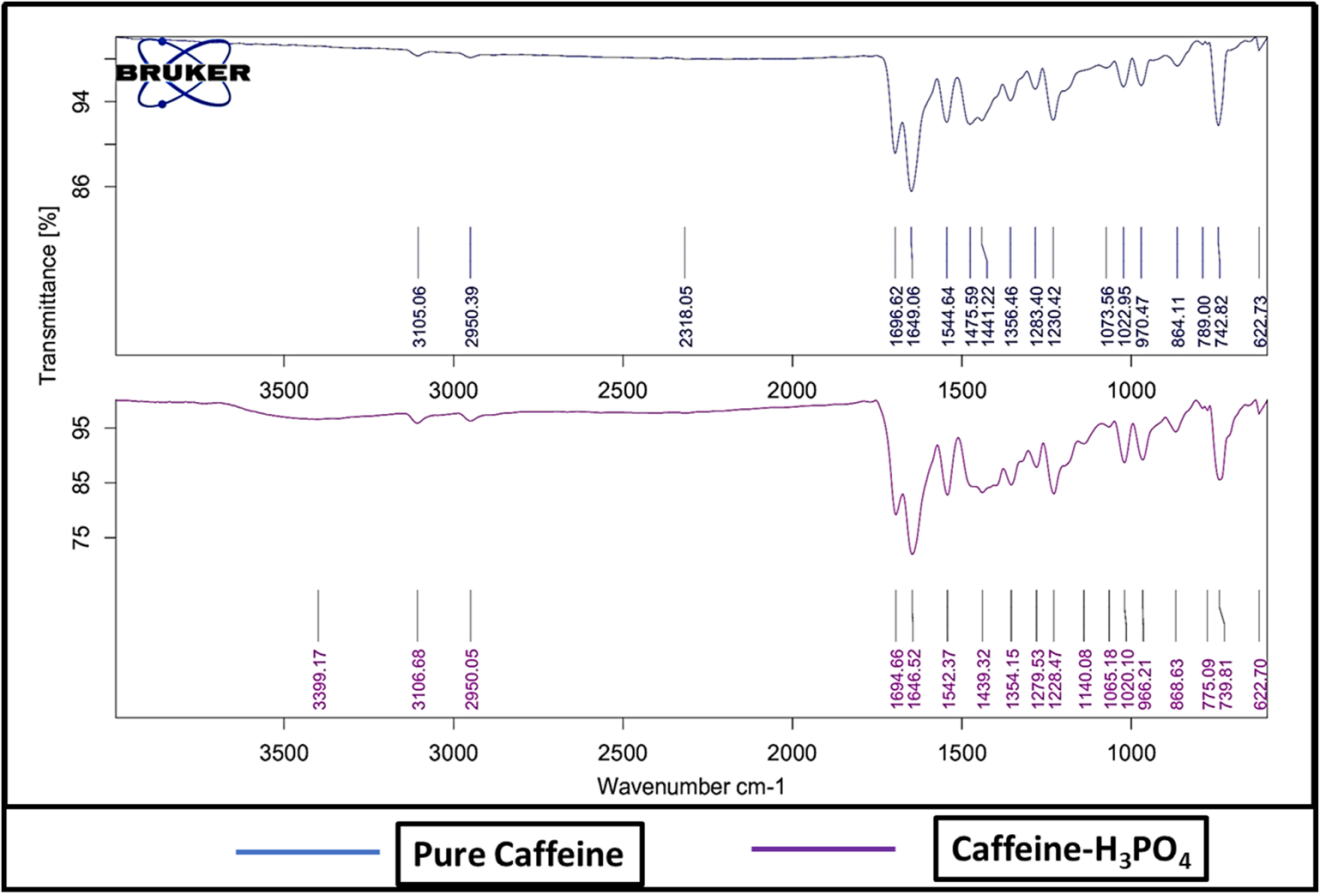
Comparative FT-IR spectra of caffeine and caffeine–H_3_PO_4_, showcasing characteristic shifts in the N–H and C=O stretching regions, indicative of molecular interaction and successful formation of the caffeine–phosphate complex.

The synthesis of the caffeine–H_3_PO_4_ catalyst was carried out by treating caffeine with phosphoric acid, followed by thorough characterization using various analytical techniques, including FT-IR, NMR, XRD, SEM, EDX, TGA, DTG, DTA, and DSC analyses. FT-IR spectra were recorded for pristine caffeine and the catalyst in the range of 500–4000 cm^−1^ to investigate the structural modifications and functional group interactions resulting from acid treatment, as illustrated in Fig. 3. FT-IR spectroscopy offers the earliest and most striking evidence of this conversion. The catalyst salt shows a broad band around 3399 cm^−1^, which corresponds to hydrogen-bonded N–H/O–H stretching created upon protonation by phosphoric acid, whereas pure caffeine shows sharp C–H stretching bands at 3105 and 2950 cm^−1^ along with strong carbonyl absorptions in the range of 1696–1649 cm^−1^. Minor shifts in the C–H stretching peaks (3106 and 2950 cm^−1^) and a downward shift in the carbonyl bands (1694 and 1646 cm^−1^) reflect subtle changes in the electronic environment induced by phosphate coordination. Furthermore, two vibrational bands appear in the fingerprint region, particularly between 1065–1020 cm^−1^ and 622 cm^−1^, characteristic of phosphate ion vibrations. These spectral modifications, combined with band broadening effects, provide compelling evidence for the successful formation of the catalyst.

**Fig. 4:**
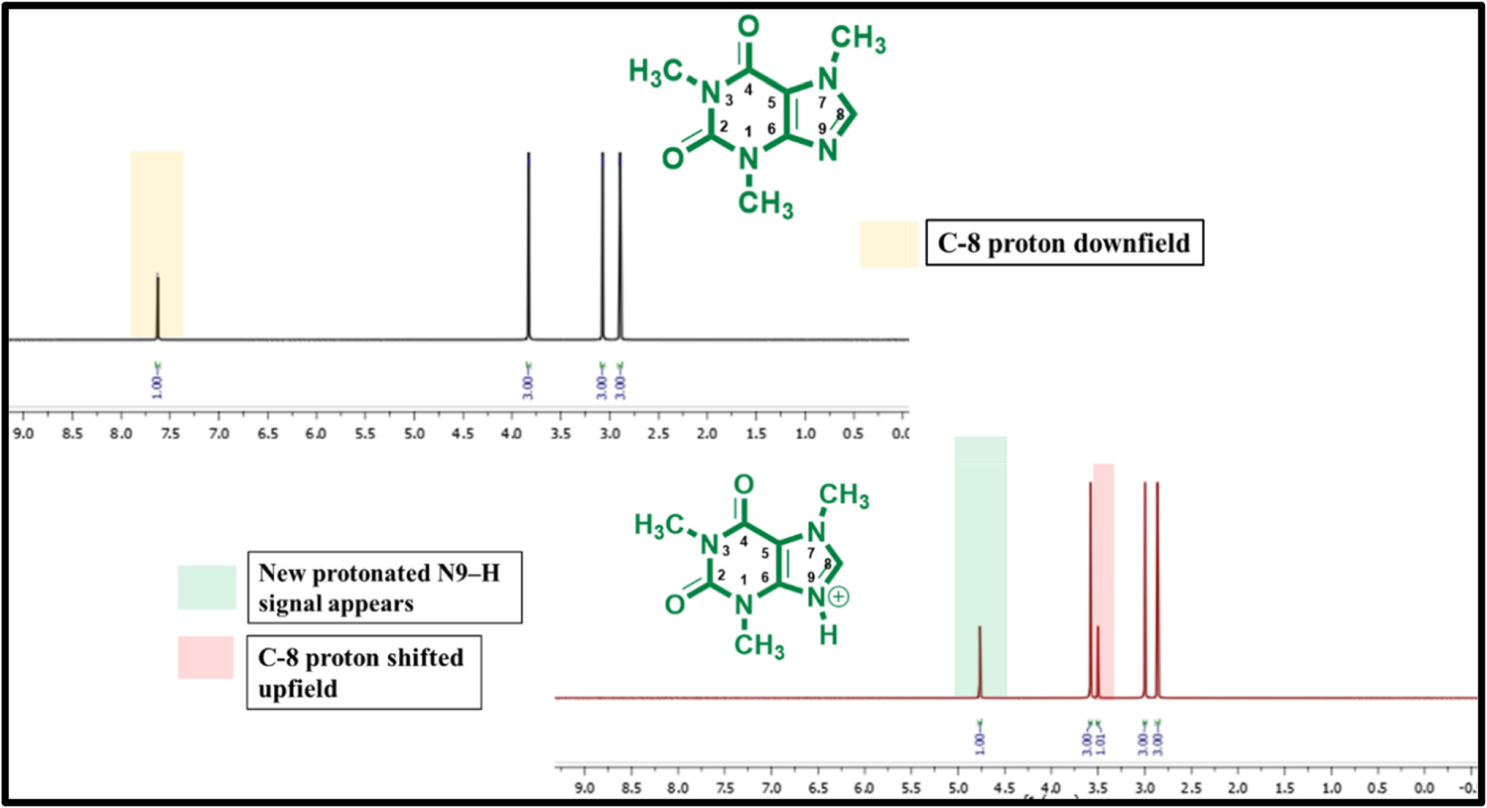
Comparative ^1^H NMR spectra of caffeine and caffeine–H_3_PO_4_, displaying distinct chemical shift variations that confirm protonation of caffeine and the formation of a stable caffeine–phosphate adduct.

The interaction between caffeine and phosphoric acid was further validated through comparative ^1^H NMR analysis, which distinctly highlighted the structural and electronic modifications occurring upon complex formation. In the spectrum of pure caffeine, the C-8 proton resonance appeared in its normal downfield position, characteristic of the neutral xanthine framework. However, when caffeine interacted with H_3_PO_4_, a clear shift in this region was observed along with the emergence of a new signal corresponding to a protonated N9–H, confirming that protonation had taken place at the nitrogen center. These spectral alterations point to a notable redistribution of electron density within the caffeine ring system, indicating the formation of a new ionic environment. The observed shifts reflect strong hydrogen-bonding interactions and proton exchange between caffeine and the phosphate moiety, leading to the stabilization of a cationic caffeine–phosphate complex. This protonation not only modifies the local chemical environment but also enhances the electron-withdrawing character of the xanthine core, which is likely responsible for its improved catalytic performance. Thus, the NMR evidence clearly demonstrates that caffeine undergoes a proton-assisted structural reorganization in the presence of phosphoric acid, resulting in an active catalytic species capable of efficient proton transfer and substrate activation in the reaction system (Fig. 4).

**Fig. 5:**
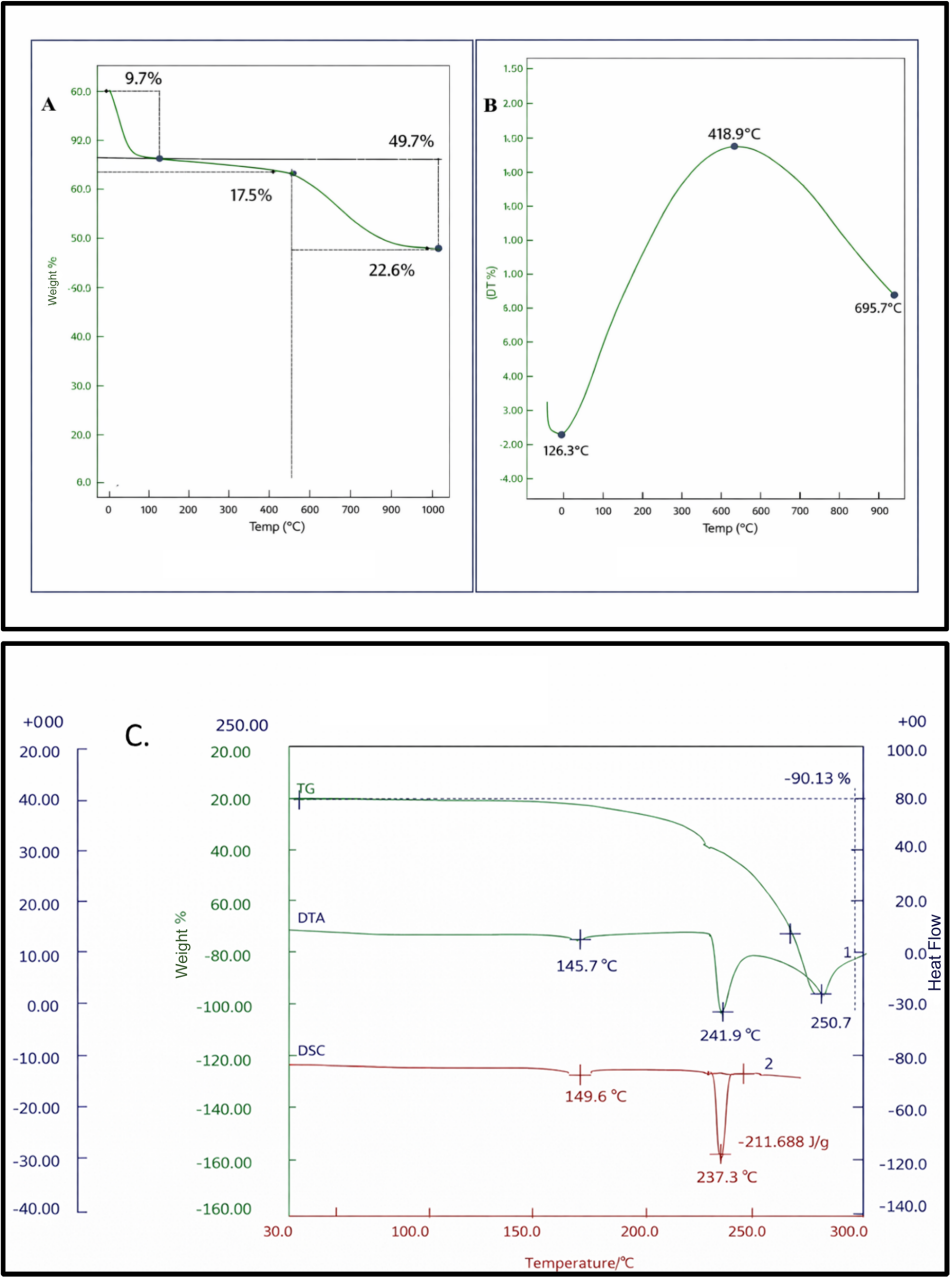
(A) TGA and (B) DTG curves (C) DTA–DSC of the catalyst recorded under an inert atmosphere.

The thermal stability and decomposition behavior of the catalyst were evaluated by TGA–DTG analysis, as presented in Fig. 5. The TGA curve exhibits an initial weight loss of approximately 9.7% below 150 °C, which can be attributed to the removal of physically adsorbed moisture and weakly bound surface species. A second gradual weight loss of about 17.5% observed up to ∼400 °C is associated with the decomposition and structural rearrangement of phosphoric acid functionalities interacting with the caffeine framework. The major weight loss step, accounting for approximately 22.6%, occurs beyond 400 °C and corresponds to the thermal degradation of the caffeine backbone. Notably, a residual mass of nearly 50% remains at higher temperatures, indicating the formation of a thermally stable carbonaceous residue stabilized by phosphate species. The DTA–DSC curves of the caffeine–H_3_PO_4_ catalyst recorded under an inert atmosphere exhibit distinct thermal events associated with structural rearrangement and phase transitions. A weak endothermic feature observed around ∼145–150 °C is attributed to molecular reorganization and strengthening of acid–base interactions between caffeine and phosphoric acid. A prominent endothermic peak appearing in the 235–245 °C range, accompanied by a sharp DSC signal, corresponds to melting and partial decomposition of the organic–acidic framework, indicating good thermal stability of the catalyst up to this temperature.

**Fig. 6:**
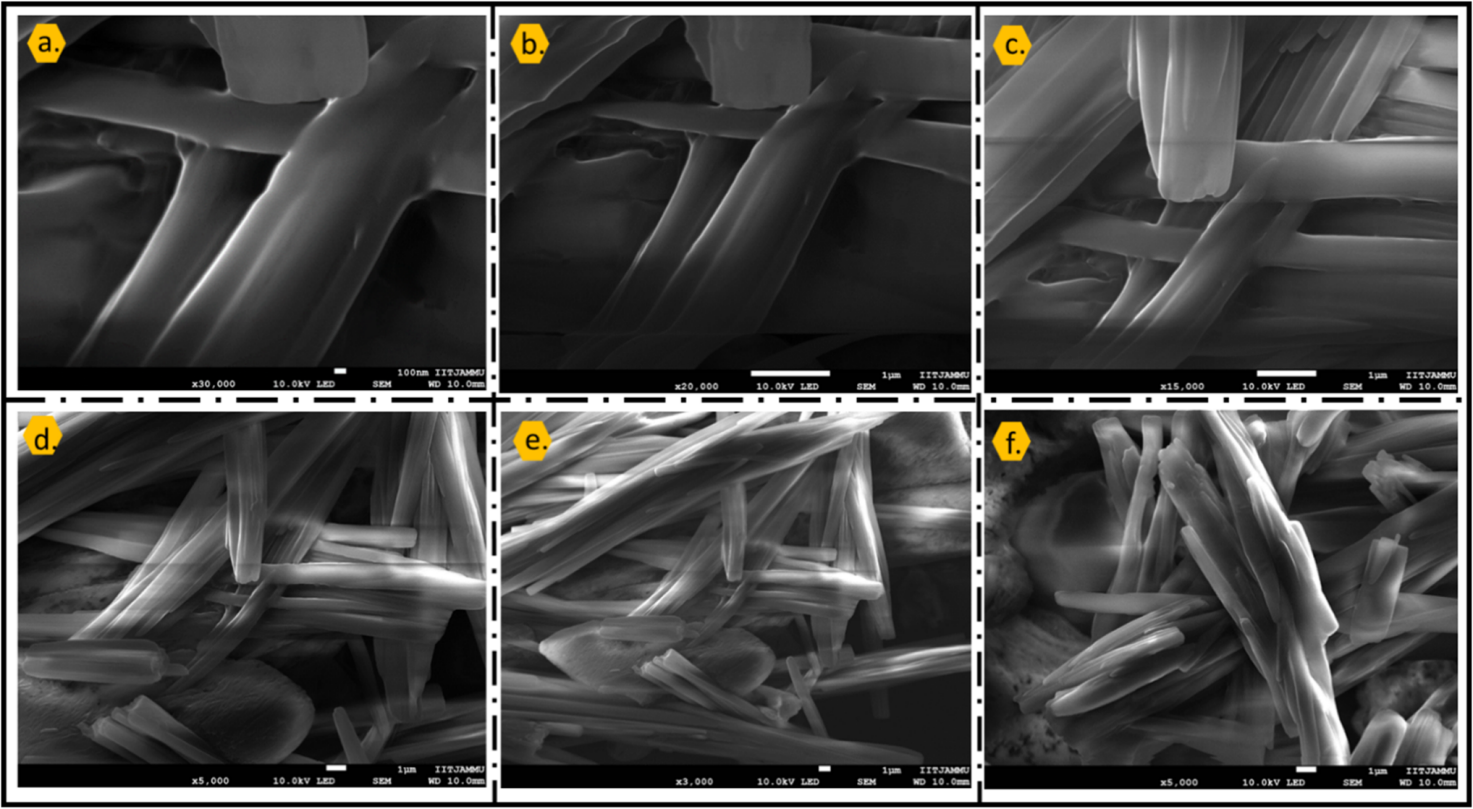
FESEM micrographs (a–f) illustrating the elongated crystalline morphology of the catalyst. High-magnification images (a–c) reveal smooth, compact crystalline domains, while lower-magnification images (d–f) show aggregation into an interwoven porous network.

The FESEM images of the catalyst, shown in Fig. 6(a)–(f), reveal a well-defined microstructural morphology composed predominantly of elongated rod- and fiber-like crystallites. At higher magnifications (Fig. 6(a)–(c)), the surface appears smooth yet compact, indicating the formation of ordered crystalline domains resulting from strong acid–base interactions between caffeine molecules and phosphoric acid. The lower-magnification images (Fig. 6(d)–(f)) clearly demonstrate the aggregation of these rod-shaped structures into an interwoven network, generating interstitial voids and channels that contribute to an overall porous architecture. Such a morphology is expected to facilitate improved accessibility of active acidic sites, thereby enhancing the catalytic performance.^53^ The uniform distribution and consistent shape of the microstructures further suggest successful incorporation of H_3_PO_4_ within the caffeine framework without severe structural collapse.

**Fig. 7:**
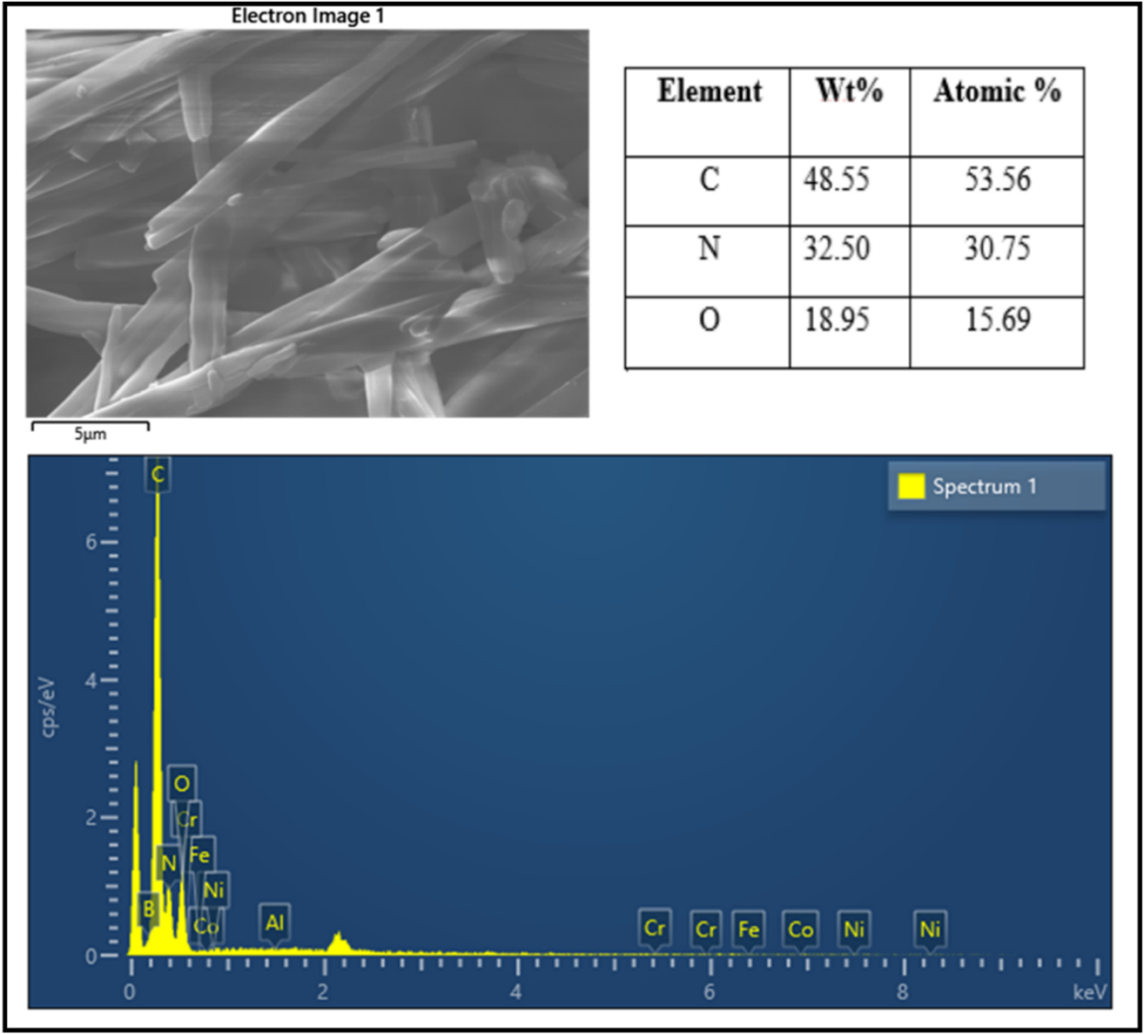
EDX analysis of the catalyst confirming the presence of its constituent elements.

The corresponding EDX spectrum verifies the presence of carbon, nitrogen, and oxygen as the main constituent elements of the catalyst. The predominance of carbon and nitrogen is attributed to the caffeine framework, whereas the incorporation of oxygen arises from phosphoric acid functionalization. Importantly, the absence of any detectable metallic signals further confirms the metal-free character and high purity of the synthesized catalyst (Fig. 7).

**Fig. 8:**
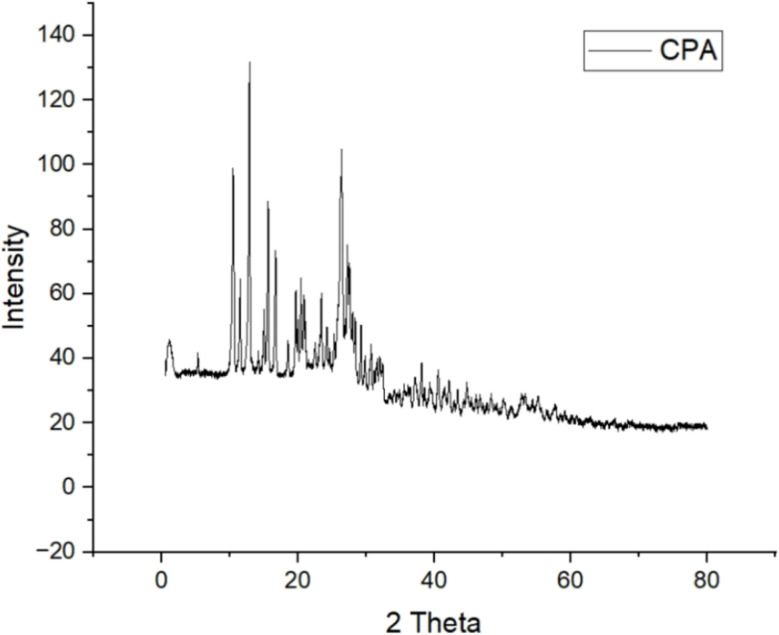
XRD pattern of the synthesized catalyst showing well-defined diffraction peaks, indicating the formation of an ordered crystalline framework.

The XRD pattern of the catalyst exhibits multiple sharp and intense peaks predominantly in the 5–30° 2*θ* range, indicating a well-defined crystalline structure. The high-intensity reflections suggest good crystallinity with distinct lattice ordering, while the gradual reduction in peak intensity at higher diffraction angles reflects a decrease in diffracted intensity with increasing 2*θ*. It is important to note that the straight line visible adjacent to the catalyst label represents the software-generated legend indicator for the plotted diffractogram and does not correspond to any diffraction peak or additional phase. Therefore, only the observed diffraction peaks within the pattern were considered for structural interpretation and phase analysis (Fig. 8).

The catalyst was comprehensively characterized using SEM, XRD, EDX, FT-IR, NMR, and TGA analyses. FT-IR and NMR confirmed the successful incorporation of acidic functionality while preserving the precursor framework. XRD indicated a well-ordered structure free of crystalline impurities, and SEM revealed a uniform microstructure. EDX verified the expected elemental composition, and LC-MS confirmed molecular integrity. TGA demonstrated excellent thermal stability, underscoring the catalyst's robustness. Together, these results establish the catalyst's structural integrity, compositional purity, and suitability for efficient heterocyclic synthesis.

A model three-component condensation involving barbituric acid, an appropriate aldehyde, and urea was carried out to optimize the conditions for the synthesis of pyrimido[4,5-*d*]pyrimidine derivatives. Initial reactions conducted without any catalyst, either in ethanol or under solvent-free conditions, led to negligible product formation, highlighting the essential role of a catalytic system (Table 1, entries 1–3). Several conventional organic acid catalysts, including sulphamic acid, citric acid, and tartaric acid, were then evaluated under identical conditions (Table 1, entries 4–6). While these acids promoted the reaction, they only afforded moderate yields with incomplete conversions and required longer reaction times. Other strategies, such as refluxing in ethanol or solvent-free grinding, were also explored but resulted in either prolonged reaction times or poor reproducibility with lower yields. In contrast, the optimized catalytic system significantly enhanced the transformation. Among the solvents tested, ethanol provided the best balance between solubility, reaction rate, and yield. Using 8–9 mol% of the catalyst in ethanol at 40 °C, the reaction proceeded efficiently to furnish the desired pyrimido[4,5-*d*]pyrimidine derivatives in high yields (typically 90–95%) within 30 minutes (Table 1, entry 9). Increasing the catalyst loading beyond this level did not improve the yield, whereas lower amounts decreased efficiency. Notably, in the absence of a catalyst, no product was obtained even after prolonged reaction times, emphasizing the critical role of the catalyst in both activating the reactants and accelerating the cyclocondensation process.

**Table 1:** Optimization of reaction conditions for the synthesis of fused pyrimidine derivative using various catalysts and highlighting the effect of different parameters on product yield and reaction efficiency

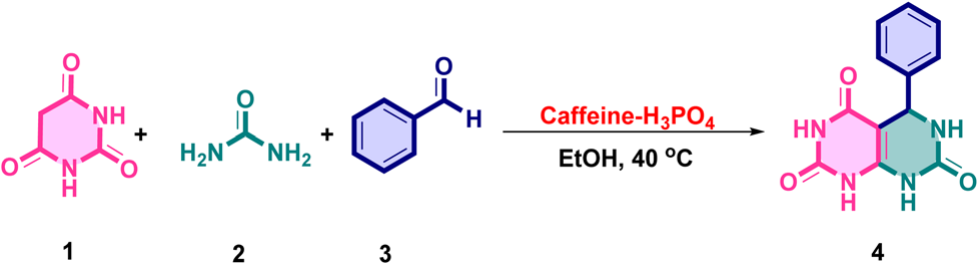						
Entry	Catalyst (mol%)	Solvent (volume)	Mode/Operation	Temp (°C)	Time (min)	Yield (%) (isolated)
1	—	EtOH (5 mL)	Grinding	RT	15	Trace
2	—	Solvent-free	Stirring/grinding	RT	120	Trace
3	—	EtOH (5 mL)	Stirring	RT	120	Trace
4	Sulfamic acid (10)	EtOH (5 mL)	Reflux	78	60	52
5	Citric acid (10)	EtOH (5 mL)	Reflux	78	60	57
6	Tartaric acid (15)	EtOH (5 mL)	Reflux	78	60	61
7	Caffeine–H_3_PO_4_ (8)	— (neat)	Grinding	RT	45	72
8	Caffeine–H_3_PO_4_ (15)	EtOH (5 mL)	Reflux	78	60	75
**9**	**Caffeine–H_3_PO_4_ (8)**	**EtOH (5 mL)**	**Stirring**	**40**	**25–30**	**90–95**
10	Caffeine–H_3_PO_4_ (8)	EtOH (5 mL)	Stirring	RT	25	75
11	Caffeine–H_3_PO_4_ (9)	EtOH (5 mL)	Stirring	RT	20	82
12	Caffeine–H_3_PO_4_ (8)	H_2_O (5 mL)	Stirring	RT	30	68

**Fig. 9:**
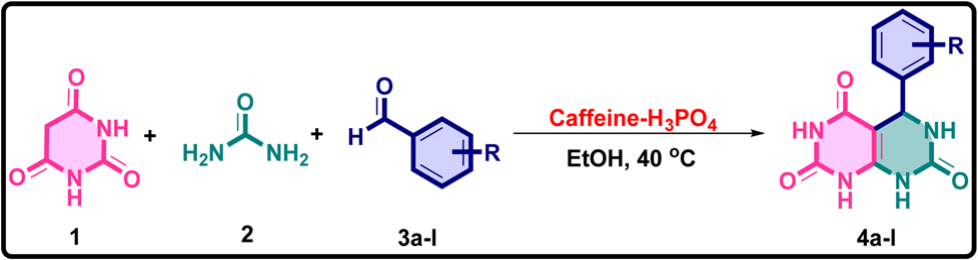
Schematic representation of the one-pot synthesis of fused pyrimidine derivatives (4a–l) catalyzed by the caffeine–H_3_PO_4_ system in ethanol.

Applying this approach to a broader range of aldehydes, the methodology was applied to a variety of substituted aromatic aldehydes, encompassing both electron-donating and electron-withdrawing groups. In all cases, the corresponding pyrimido[4,5-*d*]pyrimidine derivatives were obtained in excellent yields, demonstrating that the reaction proceeds efficiently irrespective of the electronic nature of the substituents. These outcomes collectively underscore the effectiveness of caffeine–H_3_PO_4_ as a highly efficient, recyclable, and environmentally benign catalyst. Compared to conventional organic acids, prolonged heating, or solvent-free grinding methods, this catalytic system offers superior performance, making it particularly well-suited for the rapid and sustainable synthesis of biologically relevant fused pyrimidine frameworks (Fig. 9).

**Fig. 10:**
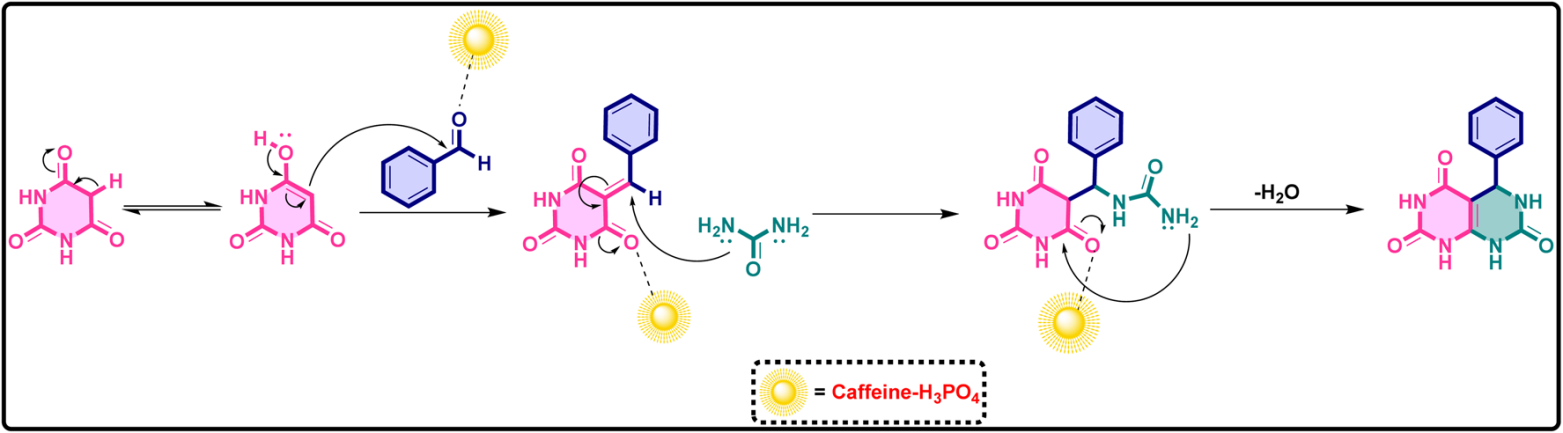
Proposed mechanism for the caffeine–H_3_PO_4_ catalyzed condensation pathway illustrating the activation of the carbonyl group, nucleophilic addition, cyclization, and subsequent dehydration steps leading to the fused pyrimidine scaffold.

The outstanding catalytic performance of the caffeine–H_3_PO_4_ system in this transformation arises from its cooperative dual-functionality, effectively integrating acid catalysis with molecular mediation. Within the reaction medium, phosphoric acid donates protons to activate the carbonyl group of the aldehyde through transient hydrogen bonding and protonation, significantly amplifying its electrophilic character. This activation enables a facile condensation with barbituric acid, giving rise to the arylidenebarbiturate intermediate *via* a classical Knoevenagel-type pathway. Concurrently, the caffeine scaffold participates as a molecular stabilizer, its π-electron-rich and heteroatom-laden framework engages in non-covalent interactions such as π–π stacking and hydrogen bonding with reactive intermediates. These interactions promote efficient substrate alignment and lower the activation barrier for subsequent transformations. In the following stage, the urea molecule, rendered nucleophilic under the mildly acidic environment, undergoes addition to the electrophilic β-carbon of the arylidenebarbiturate, leading to amide bond formation. Sequential proton rearrangements and dehydration steps culminate in the construction of the product framework. The synergy between caffeine and H_3_PO_4_ thus ensures a seamless catalytic cycle, enhancing both the rate and selectivity of the reaction under sustainable, metal-free, and solvent-efficient conditions. The plausible mechanistic sequence for the caffeine–H_3_PO_4_ catalyzed synthesis of barbiturate–urea conjugates is outlined in Fig. 10.^54^

**Fig. 11:**
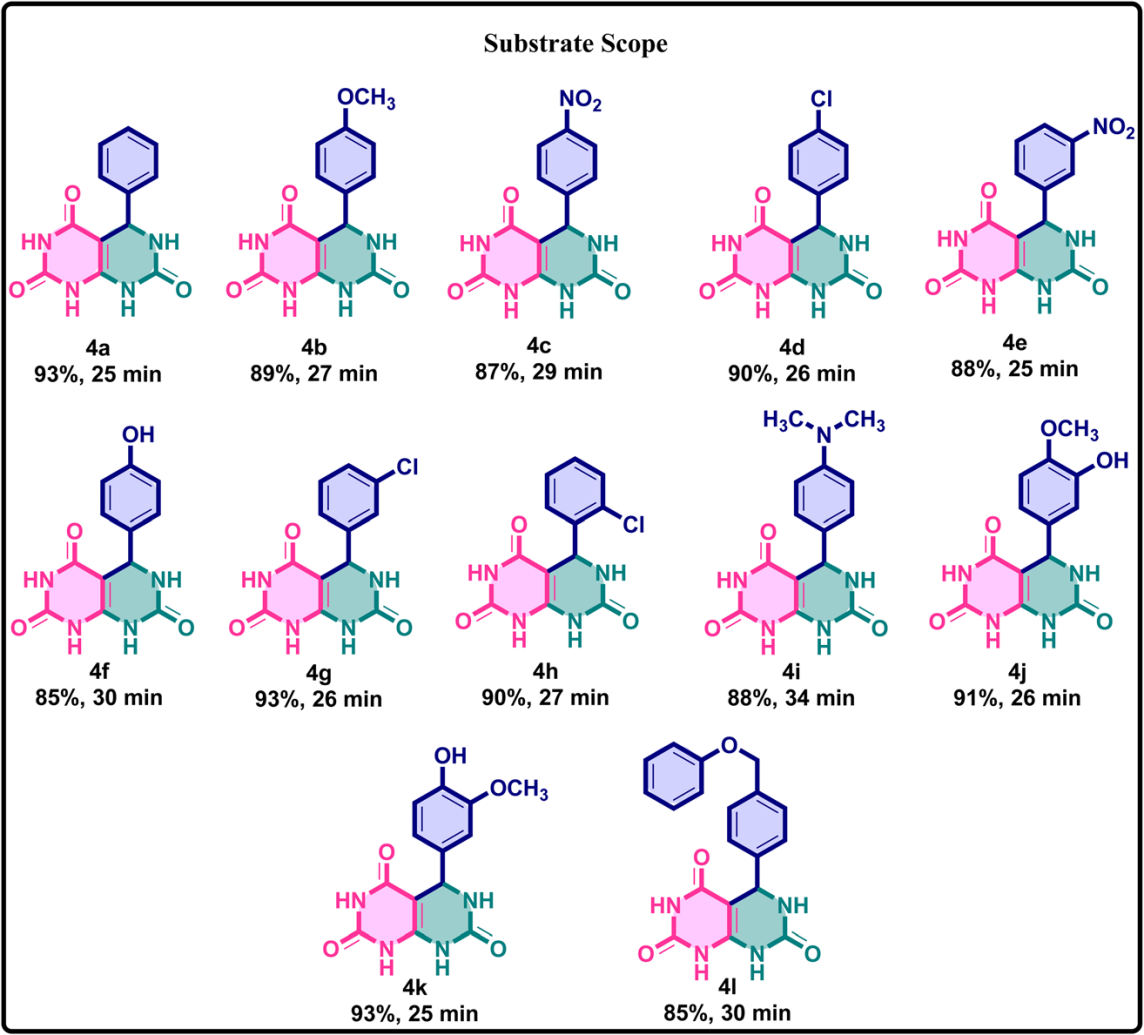
Substrate scope showing representative examples of products obtained with different substituted aromatic aldehydes bearing electron-donating and electron-withdrawing groups under the optimized reaction conditions.

Building on the optimized protocol, a series of derivatives were subsequently synthesized under identical reaction conditions, demonstrating the versatility and reproducibility of the improved method. The approach consistently afforded high yields and purity, highlighting its efficiency and broad applicability for heterocyclic synthesis (Fig. 11).

### Docking analysis

To investigate the potential biological relevance of the synthesized fused pyrimidine derivatives (4a–l), molecular docking studies were carried out against two biologically important enzymes, dihydrofolate reductase (DHFR, PDB ID: 2FZI) and cyclin-dependent kinase 2 (CDK2, PDB ID: 3PXQ). DHFR plays a crucial role in folate metabolism and nucleotide biosynthesis, and its inhibition is a well-established strategy in antimicrobial and antitubercular drug discovery. CDK2 is a key regulatory enzyme involved in cell-cycle progression and DNA replication, and its dysregulation is closely associated with uncontrolled cellular proliferation and cancer development. Docking simulations were performed to evaluate the binding orientations, key intermolecular interactions, and stability of the synthesized compounds within the active sites of both enzymes. This comparative computational analysis provided insights into how subtle structural variations among the derivatives influence their binding affinities and molecular recognition patterns toward each target. The results offer a theoretical framework for understanding their prospective DHFR- and CDK2-inhibitory behavior and support further experimental biological validation studies.

### Binding profiles of compounds for CDK2 protein

**Fig. 12:**
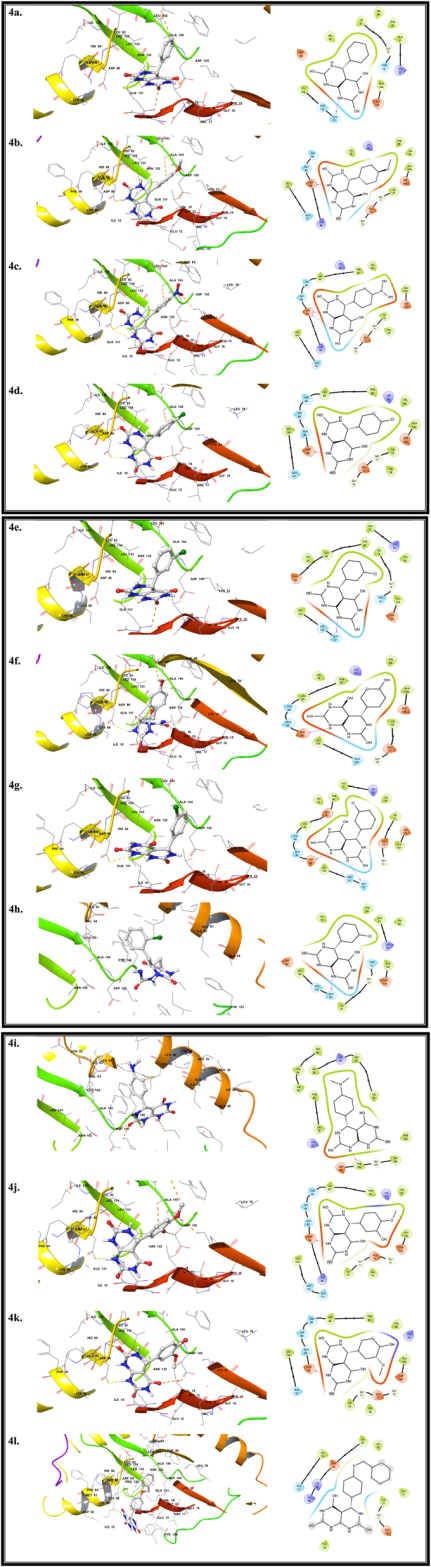
Interaction analysis of the synthesized compounds with the 3PXQ target, shown through both spatial (3D) and schematic (2D) representations.

To elucidate the potential biological interactions of the synthesized fused pyrimidine derivatives (4a–l), molecular docking studies were conducted to assess the binding affinities and interaction patterns of compounds with CDK2. The obtained docking scores ranged from −8.7 to −10.8 kcal mol^−1^, indicating strong to moderate binding affinities of the ligands. Among these, compound 4e displayed the highest docking score (−10.8 kcal mol^−1^), signifying the most favorable binding energy and stable interaction, followed closely by 4g (−10.1 kcal mol^−1^) and 4a (−10.0 kcal mol^−1^). Derivatives possessing electron-withdrawing substituents such as –NO_2_ and –Cl (compounds 4e, 4g, and 4h) generally exhibited enhanced binding affinities, attributed to their improved electronic compatibility with the enzyme's active pocket. Compounds 4i, 4j, and 4k showed moderate docking scores ranging from −8.7 to −9.3 kcal mol^−1^, suggesting acceptable interaction stability within the binding site. Interestingly, compound 4l also demonstrated a comparatively high docking score (−9.5 kcal mol^−1^), highlighting its potential as an additional active lead molecule within the synthesized series (Fig. 12).

Overall, the docking analysis suggests that the caffeine–H_3_PO_4_ catalyzed fused pyrimidine derivatives possess notable binding potential, with 4e, 4g, and 4l emerging as the most promising candidates for further biological evaluation (Table 2).

**Table 2:** Amino acid residues involved in the molecular interactions between the synthesized compound and the target protein, illustrating key hydrogen bonds, hydrophobic contacts, and binding site affinities

Pose name	Docking score	Amino acid interaction
4a	−10	Ala116, Ala279, Asn132, Asp258, Gln113, Gln265, Gln287, **Gly114**, His119, His121, His125, His161, His268, His283, His295, His60, His71, His84, **Ile10**, Leu128
4b	−9.2	Ala144, Ala31, Asn132, Asp86, **Gly13**, Ile10, **Leu134**, Phe80, **Thr14**, **Val18**, Val64
4c	−9	**Ala116**, Ala279, Asn132, Asp258, Gln113, Gln265, Gln287, Gly114, His119, His121, His125, His161, His268, His283, His295, His60, His71, His84, **Ile10**, Leu128, Leu281, Leu298
4d	−9.1	**Ala144**, Gln131, **Gly11**, Hid84, Ile10, Leu134, Lys89, Phe80, Val18val64
4e	−10.8	Ala116, Ala279, Asn132, **Leu143**, Asp258, **Gln131**, Gln265, **Gln287**, Gly114, His119, His121, His125, His161, His268, His283, His295, His60, His71, His84, Ile10
4f	−9.1	Ala144, Gly11, Hid84, Ile10, **Leu134**, Gln131, Leu83, **Lys89**, Phe80, Val18, Val64
4g	−10.1	Ala116, Ala279, Asn132, **Asp258**, Gln113, **Gln265**, Gln287, Gly114, His119his121, His125, His161, His268, His283, His295, His60, His71, His84, Ile10,
4h	−9.6	Ala144, Asp145, **Gln131**, Gly11, Gly13, His84, **Ile**10, **Leu134**, Lys33, Phe80, Val18, Val64
4i	−8.8	Ala116, Ala279, Asn132, **Asp258**, Gln113, Gln265, Gln287, **Gly114**, His119, His121, His125, His161, His268, His283, His295, His60, His71, His84, Ile10
4j	−8.7	Ala144, Ala31, Asn132, **Asp86**, Gly13, Ile10, **Leu134**, Leu83, Phe80, Thr14, Val18, Val64
4k	−9.3	Ala116, **Ala279**, Asn132, Asp258, Gln113, **Gln265**, Gln287, Gly114, His119, His121, His125, His161, His268, His283, His295, His60, His71, His84, Ile10
4l	−9.5	Ala31, Asn132, **Gly11**, Gly13, Ile10, **Leu134**, Leu83, Lys33, Phe80, Thr14, Val18, Val64

### Binding profiles of compounds with DHFR protein

**Fig. 13:**
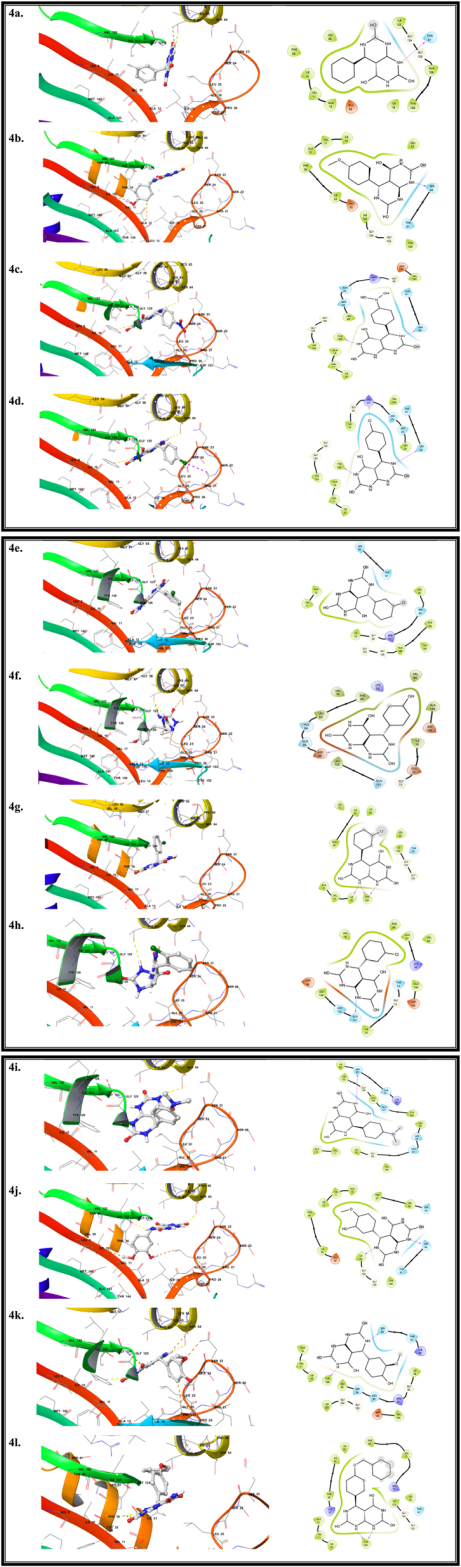
Interaction analysis of the synthesized compounds with the 2FZI target, shown through both spatial (3D) and schematic (2D) representations.

Docking simulations were performed for compounds 4a–l to investigate their binding behavior within the active site of DHFR. All compounds demonstrated appreciable binding affinities, with docking scores ranging from −8.3 to −10.8 kcal mol^−1^, indicating stable ligand–enzyme interactions. Among the evaluated compounds, 4l exhibited the strongest binding affinity, with a docking score of −10.8 kcal mol^−1^, suggesting the formation of a highly stable complex. Other derivatives, including 4g, 4h, 4c, and 4f, also showed favorable binding energies, reflecting efficient accommodation within the enzyme pocket. Analysis of the binding modes revealed that ligand stabilization is mainly governed by a combination of hydrophobic interactions and polar contacts with key active-site residues. Amino acids such as histidine, aspartate, glutamate, isoleucine, leucine, valine, and alanine were repeatedly involved, underscoring their importance in maintaining binding stability. The recurrent participation of hydrophobic regions together with histidine-rich residues supports strong ligand anchoring within the catalytic cavity (Fig. 13).

Overall, the docking results indicate that the synthesized compounds possess good binding compatibility with the DHFR active site, with 4l emerging as a promising lead candidate for further biological and mechanistic investigations (Table 3).

**Table 3:** Key amino acid residues involved in the molecular interactions between the synthesized compounds and the target protein, highlighting hydrogen bonding, hydrophobic contacts, and binding site interactions

Pose name	Docking score	Amino acid interaction
4a	−8.3	Ala126, Glu32, Gly124, Ile10, Ile123, Ile19, Leu25, Phe36, **Thr61**, Tyr129, Val11
4b	−8.9	**Ala95**, Asp149, Glu172, His100, His135, His151, His174, His187, His93, **Ile76**, Leu102, Leu98, Lys96, Met133, Pro26, Thr108, Val78
4c	−9.3	Gly124, **Ile10**, Ile33, Leu25, Phe36, Tyr129, Val11
4d	−8.9	Ala95, Asp149, Glu172, His100, His135, His151, His174, His187, His93, Ile76, Leu102, Leu98, Lys96, Met133, Pro26, **Tyr108**, Val78
4e	−8.8	Ala12, Asp153, **Gly124**, Gly20, Ile10, Leu25, Val11, Val154
4f	−9.3	Ala95, **Asp149**, Glu172, His100, His135, His151, His174, His187, His93, Ile76, Leu102, Leu98, Lys96, Met133, Pro26, **Thr108**, Val78
4g	−9.5	Ala12, Arg21, Asn23, **Gly124**, Gly20, Ile10, Ile19, Leu25, Ser24, Val11, Val154
4h	−9.4	Ala95, Asp149, Glu172, His100, His135, His151, His174, His187, His93, Ile76, Leu102, Leu98, **Lys96**, Met133, Pro26, **Thr108**, Val78
4i	−8.5	Ala126, Arg21, Gly124, Gly20, Ile19, Leu25, **Ser24**, Val11, Val154
4j	−8.9	Ala95, Asp149, Glu172, His100, His135, His151, His174, His187, His93, **Ile76**, Leu102, **Leu98**, Lys96, Met133, Pro26, Thr108, Val78
4k	−9	Asn23, **Gly125**, Gly20, Ile10, **Ile123**, Ile19, Leu25, Ser24, **Thr129**, **Val11**, Val154
4l	−10.8	Ala95, Asp149, Glu172, His100, His135, His151, His174, His187, His93, Ile76, Leu102, Leu98, Lys96, Met133, Pro26, **Tyr108**, Val78

### Physicochemical properties of the synthesized compounds

Table 4 summarizes the key physicochemical attributes of the synthesized molecules. All compounds fall within the acceptable molecular weight range for drug-like candidates, while their iLOGP values indicate balanced lipophilicity. The number of hydrogen bond donors and acceptors aligns well with established drug-likeness criteria. Additionally, the molecular refractivity and TPSA values suggest favorable potential for bioavailability. Collectively, these findings demonstrate that the compounds strongly comply with Lipinski's rule of five, supporting their suitability for further pharmacological evaluation.

**Table 4:** Physicochemical properties of the synthesized compounds, confirming compliance with Lipinski's rule of five and favorable drug-like characteristics

Molecule	H-bond acceptors	H-bond donors	MR	TPSA	iLOGP	Lipinski violations
4a	3	4	74.57	106.85	0.75	0
4b	4	4	81.06	116.08	1.05	0
4c	5	4	83.39	152.67	0.4	0
4d	3	4	79.58	106.85	1.02	0
4e	3	4	79.58	106.85	1.02	0
4f	4	5	76.59	127.08	0.42	0
4g	3	4	79.58	106.85	1.02	0
4h	3	4	79.58	106.85	0.98	0
4i	3	4	88.78	110.09	1.04	0
4j	5	5	83.08	136.31	0.9	0
4k	5	5	83.08	136.31	0.95	0
4l	4	4	105.55	116.08	1.68	0

### Drug-likeness, pharmacokinetic behavior, and physicochemical attributes

The ADME properties of the synthesized pyrimidine derivatives (4a–l) were predicted through computational analysis to assess their pharmacokinetic suitability. All the compounds displayed moderate Caco-2 permeability (around −5.1 to −6.2), indicating acceptable intestinal transport potential. Their intestinal absorption was predicted to be high, suggesting that the molecules can be efficiently absorbed through the gastrointestinal tract. The skin permeability (log *K*_p_) values reflected limited penetration through the skin, which is typical for compounds with relatively larger molecular structures. None of the molecules were identified as P-glycoprotein (P-gp) substrates or inhibitors, implying that their bioavailability is unlikely to be affected by efflux transport mechanisms. The predicted volume of distribution (VDss) values (0.41–0.86 L kg^−1^) and fraction unbound (fu) values (0.33–1.27) suggest moderate tissue distribution with an adequate level of free drug available in circulation. The predicted BBB permeability (log BB ≈ −2.0 to −3.2) indicates limited passage across the blood–brain barrier, while most of the derivatives were classified as CNS permeant, implying that certain compounds may still reach central nervous targets. In terms of metabolic behavior, all compounds were found to be non-substrates of CYP3A4 and non-inhibitors of CYP1A2 and CYP2D6, suggesting lower risk of metabolic interference. However, most of the derivatives showed inhibitory potential toward CYP2C19 and CYP2C9 isoenzymes, indicating possible influence on drugs metabolized by these enzymes. The total clearance values (3.2–9.4 mL min^−1^ kg^−1^) point to moderate elimination, while the absence of renal OCT2 substrate activity suggests a negligible likelihood of renal transporter-mediated excretion. Collectively, these *in silico* findings indicate that the synthesized derivatives possess favorable pharmacokinetic profiles, combining efficient absorption, balanced distribution, moderate metabolic stability, and safe clearance properties, making them suitable candidates for further biological evaluation (Table 5).

**Table 5:** ADMET properties of the synthesized compounds, highlighting their absorption, distribution, metabolism, excretion, and toxicity profiles

Compound	Absorption					Distribution				Metabolism							Excretion
	Caco2 permeability	Intestinal absorption (%)	Skin permeability	P-gp substrate	P-gp I inhibitor	VDss (human)	Fraction unbound	BBB permeability	CNS permeability	CYP3A4 substrate	CYP1A2 inhibitor	CYP2C19 inhibitor	CYP2C9 inhibitor	CYP2D6 inhibitor	CYP3A4 inhibitor	Total clearance	Renal OCT2 substrate
4a	−5.74	Abs	−1.38	NS	NS	0.55	1.06	−2.16	Pen	NI	NI	NS	Inhib	Inhib	NS	5.92	NI
4b	−5.74	Abs	−1.29	NS	NS	0.48	0.99	−2.21	Pen	NI	NI	NS	Inhib	Inhib	NS	6.14	NI
4c	−5.74	Abs	−1.29	NS	NS	0.48	0.99	−2.21	Pen	NI	NI	NS	Inhib	Inhib	NS	6.14	NI
4d	−5.95	Abs	−1.28	NS	NS	0.45	0.57	−3.22	NPen	NI	NI	NS	Inhib	Inhib	NS	9.42	NI
4e	−5.97	Abs	−1.38	NS	NS	0.48	0.86	−3.14	NPen	NI	NI	NS	Inhib	Inhib	NS	9.3	NI
4f	−5.52	Abs	−1.39	NS	NS	0.86	1.01	−2.79	Pen	NI	NI	NS	Inhib	Inhib	NS	3.48	NI
4g	−5.71	Abs	−0.76	NS	NS	0.49	0.61	−2.26	Pen	NI	NI	NS	Inhib	Inhib	NS	5.97	NI
4h	−5.14	Abs	−1.3	NS	NS	0.78	0.33	−2.07	Pen	NI	NI	NS	Inhib	Inhib	NS	8.73	NI
4i	−5.74	Abs	−2.49	NS	NS	0.78	0.61	−2.62	Pen	NI	NI	NS	Inhib	Inhib	NS	9.25	NI
4j	−5.18	Abs	−1.09	NS	NS	0.41	0.57	−2.7	Pen	NI	NI	NS	Inhib	Inhib	NS	4.4	NI
4k	−6.18	Abs	−1.83	NS	NS	0.49	0.57	−2.16	Pen	NI	NI	NS	Inhib	Inhib	NS	5.92	NI
4l	−5.34	Abs	−1.37	NS	NS	0.68	1.27	−2.3	Pen	NI	NI	NS	Inhib	Inhib	NS	3.21	NI

### Cytotoxic potential

Using the MTT assay, the cytotoxic potential of the synthesized derivatives (4i–l) was evaluated against the chosen cancer cell line. The percentage of live cells was calculated in comparison to the control (DMSO-treated) after cells were exposed to different concentrations (10–100 µM) of each substance for a whole day. Table 6 provides a summary of the findings. Among the tested derivatives, compounds 4i–l exhibited moderate to good cytotoxic activity in a concentration-dependent manner. The cell viability decreased progressively with increasing compound concentration, indicating dose-dependent inhibition of cell proliferation. Notably, compound 4i showed the highest cytotoxic effect, reducing cell viability to 55.67% at 100 µM, which is comparable to the standard drug CP under identical conditions. These results imply that adding particular substituents to the core framework is crucial for increasing the synthetic derivatives' cytotoxic effectiveness.

**Table 6:** Cytotoxicity data of the synthesized compounds against selected cell lines, showing cell viability percentages and dose-dependent effects

Compound	Concentration (µM)	Cell viability (%) ± SD
Control (DMSO)	—	98.60 ± 0.84
4i	10	62.10 ± 0.97
	25	60.45 ± 1.10
	50	57.98 ± 1.08
	100	55.67 ± 1.12
4j	10	61.86 ± 1.05
	25	59.92 ± 1.02
	50	57.12 ± 1.03
	100	55.03 ± 1.15
4k	10	60.45 ± 0.98
	25	58.33 ± 1.07
	50	56.04 ± 1.11
	100	54.67 ± 1.20
4l	10	61.20 ± 1.00
	25	59.15 ± 1.08
	50	57.00 ± 1.16
	100	54.38 ± 1.22
CP (std.)	—	62.35 ± 1.05

**Fig. 14:**
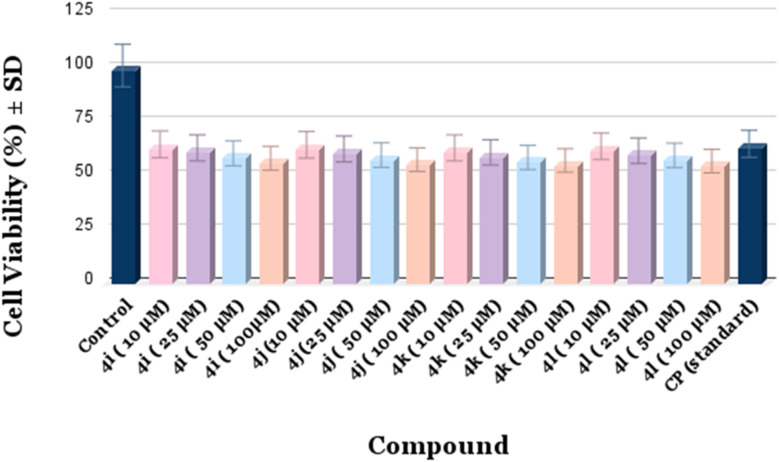
Bar graph representing the cytotoxicity of the synthesized compounds against selected cell lines, illustrating dose-dependent effects and relative cell viability.

To assess the anticancer potential of the compounds, cell viability assays were conducted, and the corresponding results are presented in the bar graph (Fig. 14).

### Antibacterial screening

The antibacterial activities of the synthesized derivatives (4a–l) were evaluated against four bacterial strains: *B. cereus*, *L. monocytogenes*, *E. coli*, and *P. aeruginosa*. The minimum inhibitory concentration (MIC) values, summarized in Table 7, revealed that all derivatives exhibited significant antibacterial activity, with MICs ranging from 4 to 12 µg mL^−1^. Among the compounds, 4j emerged as the most potent, showing particularly strong inhibition against *B. cereus* and *L. monocytogenes*. In contrast, 4a and 4b demonstrated comparatively moderate activity, while the other derivatives displayed varying levels of efficacy across the tested strains. The standard control (CP) exhibited the lowest MICs, in agreement with its well-established antibacterial profile. Overall, these results confirm that the synthesized derivatives are effective in inhibiting the growth of both Gram-positive and Gram-negative bacteria. A closer examination of the data suggests that the observed antibacterial activity is closely related to the structural features of the derivatives. Certain substitutions appear to enhance potency, particularly against specific bacterial strains, highlighting the importance of molecular design in determining biological activity.

**Table 7:** Minimum inhibitory concentration (MIC) values (µg mL^−1^) of the synthesized compounds and standard drug against selected bacterial strains

Compound	Minimum inhibitory concentration (µg mL^−1^)			
	*B. cereus*	*L. monocytogenes*	*E. coli*	*P. aeruginosa*
4a	10	11	12	12
4b	9	10	11	12
4c	8	9	10	11
4d	7	8	9	11
4e	6	7	8	9
4f	5	6	7	8
4g	6	7	8	9
4h	8	9	10	11
4i	7	8	9	11
4j	4	5	6	7
4k	5	6	7	8
4l	6	7	8	9
CP (std.)	2	3	2	4

**Fig. 15:**
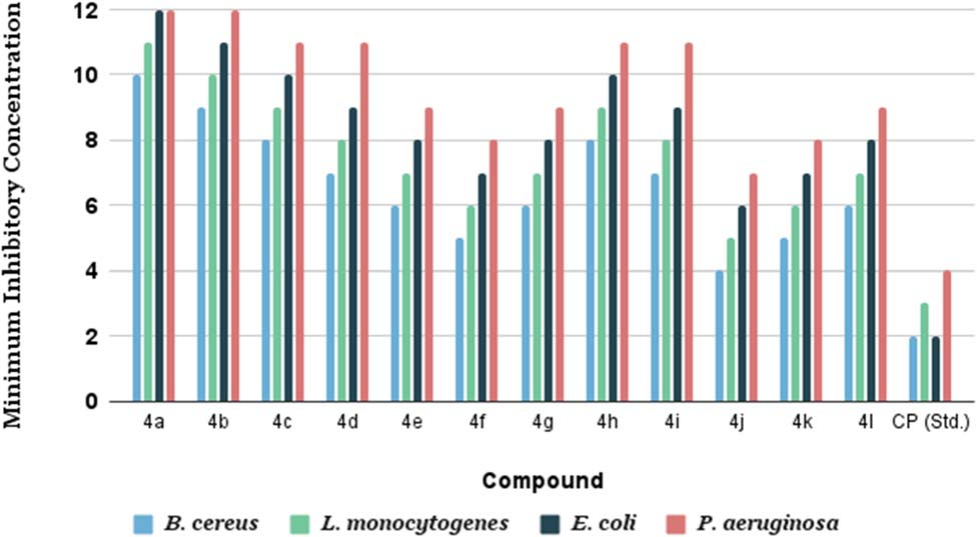
Comparative bar graph of minimum inhibitory concentration (MIC) values (µg mL^−1^) of the synthesized compounds against Gram-positive and Gram-negative bacterial strains.

The variations in MIC values across the series provide insight into structure–activity relationships, offering a valuable framework for the future design and optimization of more effective antibacterial agents. Collectively, these findings demonstrate that the synthesized derivatives represent a promising scaffold for further exploration in antibacterial drug development and underscore their potential for application against a range of pathogenic bacteria (Fig. 15).

### Gram-scale synthesis

**Fig. 16:**
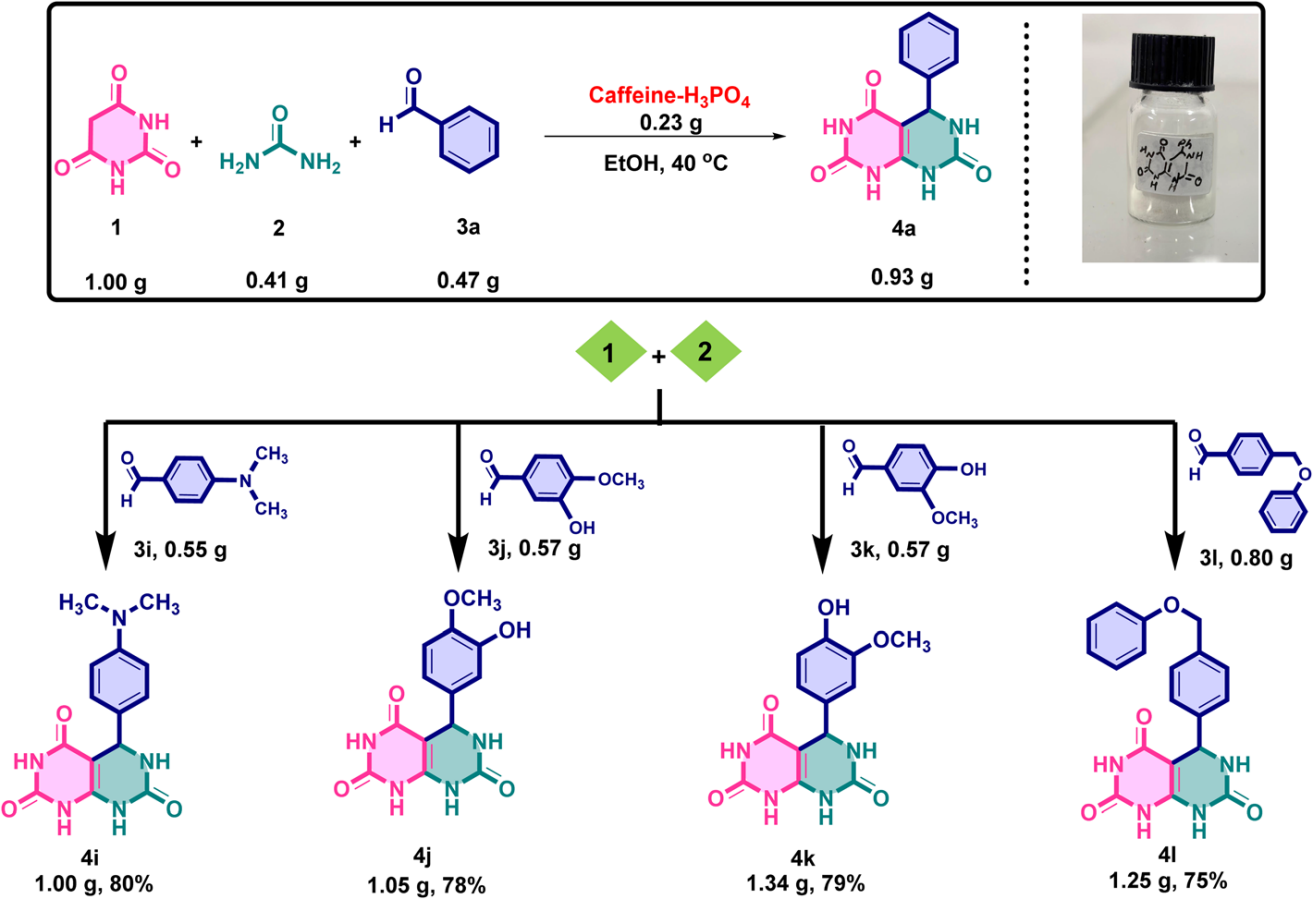
Gram-scale synthesis of the target fused pyrimidine derivatives, demonstrating the scalability and practical applicability of the optimized reaction protocol.

To evaluate the scalability and efficiency of the developed caffeine–H_3_PO_4_-catalyzed protocol, a gram-scale synthesis of the target pyrimido[4,5-*d*]pyrimidine derivative was initially attempted using benzaldehyde as the model substrate. Barbituric acid (1.00 g, 7.8 mmol), urea (0.41 g, 6.8 mmol), and benzaldehyde (0.47 g, 3.9 mmol) were subjected to condensation in ethanol (20 mL) at 40 °C in the presence of caffeine–H_3_PO_4_ (0.23 g, 8 mol%) as a catalyst. The reaction proceeded smoothly, affording the desired product 4a as white crystalline solid in 88% yield (0.93 g) after recrystallization from ethanol. Encouraged by this result, the methodology was subsequently extended to a variety of substituted aromatic aldehydes to examine its substrate scope and general applicability. The reactions furnished the corresponding derivatives (4i–l) in excellent isolated yields ranging from 75–80%, specifically: 4i (1.00 g, 80%), 4j (1.05 g, 78%), 4k (1.34 g, 79%), and 4l (1.25 g, 75%). The outcomes clearly demonstrate the robustness and efficiency of the catalytic system under mild conditions, confirming its suitability for gram-scale synthesis. The representative reaction scheme and the obtained products are illustrated in Fig. 16.

### Green chemistry assessment

The green chemistry evaluation of the synthesized fused pyrimidine derivatives (4a–l) demonstrated excellent sustainability performance based on key parameters. The AE (79.5–83.9%), RME (69.0–75.5%), E-factor (0.32–0.45), and PMI (14.4–18.4) values collectively indicate that the developed method is both efficient and environmentally favorable. All reactions proceeded efficiently with high yields (85–93%), confirming the reliability of the adopted procedure. The low E-factor values reflected minimal waste formation, while the PMI values suggested optimal material utilization with limited solvent and reagent consumption. The AE and RME values revealed that a major portion of the reactant mass was effectively converted into the desired products, minimizing the generation of unwanted by-products.

Overall, these results confirm that the present synthetic route provides a green, atom-economical, and sustainable approach for the preparation of fused pyrimidine derivatives, aligning well with the core principles of green chemistry.

## Experimental

### Standard protocol for the preparation of the catalyst

**Fig. 17:**
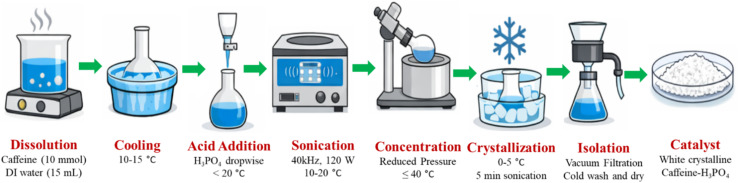
Schematic representation of the sonication-assisted synthesis of caffeine–H_3_PO_4_. The diagram outlines the key steps involved in salt formation and isolation.

A clear solution was achieved by gently heating 15 mL of deionized water with caffeine (1.94 g, 10.0 mmol) to 40–50 °C. After letting the solution settle to room temperature, it was transferred into a 100 mL round-bottom flask that was kept between 10 and 15 °C in an ice bath. After that, phosphoric acid (H_3_PO_4_, 85%, 1.0 mL, 10.0 mmol) was added dropwise over the course of five to seven minutes while being continuously stirred magnetically to keep the temperature below 20 °C. To encourage full protonation and salt production, the mixture was then sonicated for 15 minutes at 10–20 °C in an ultrasonic bath (40 kHz, 120 W). In order to induce crystallization, the resultant solution was concentrated under reduced pressure at ≤40 °C to near saturation, cooled to 0–5 °C, and then briefly sonicated for five minutes. To obtain a white crystalline product, the solid caffeine–H_3_PO_4_ was collected by vacuum filtration under cold circumstances, cleaned with ice-cold water and a little amount of cold acetone, and dried under reduced pressure at 40 °C (Fig. 17).

### Recycling of the catalyst

**Fig. 18:**
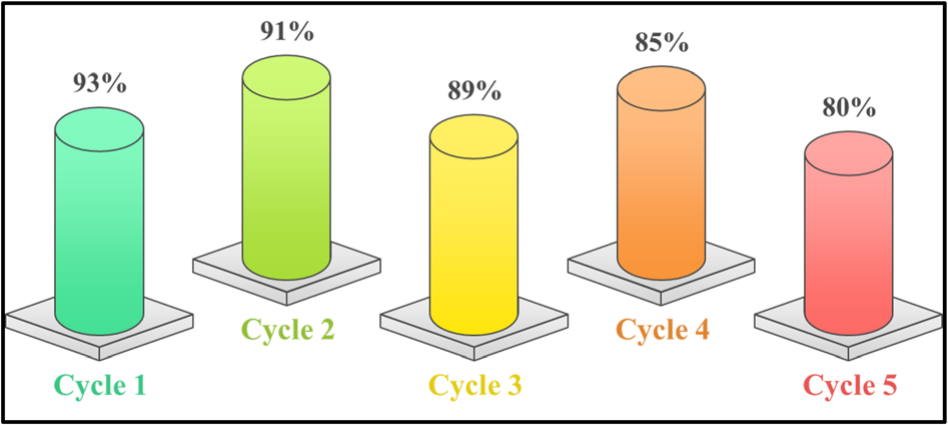
Sustainability profiling of the caffeine–H_3_PO_4_ catalyst through repeated recyclability tests.

In order to assess the catalyst's potential as a sustainable green catalyst, its reusability was investigated. Following each reaction, the mixture was quenched, the catalyst was recovered using a straightforward aqueous work-up, and water was then removed under low pressure. The recovered solid was dried under vacuum at 40 °C, cleaned with ethanol to remove any remaining organic contaminants, and then used straight away in the subsequent cycle without additional purification. The catalyst showed good stability and recyclability under the applied reaction conditions by maintaining high activity across four successive cycles, with the product yield marginally declining from 93% in the first run to 80% in the fifth run (Fig. 18).

**Fig. 19:**
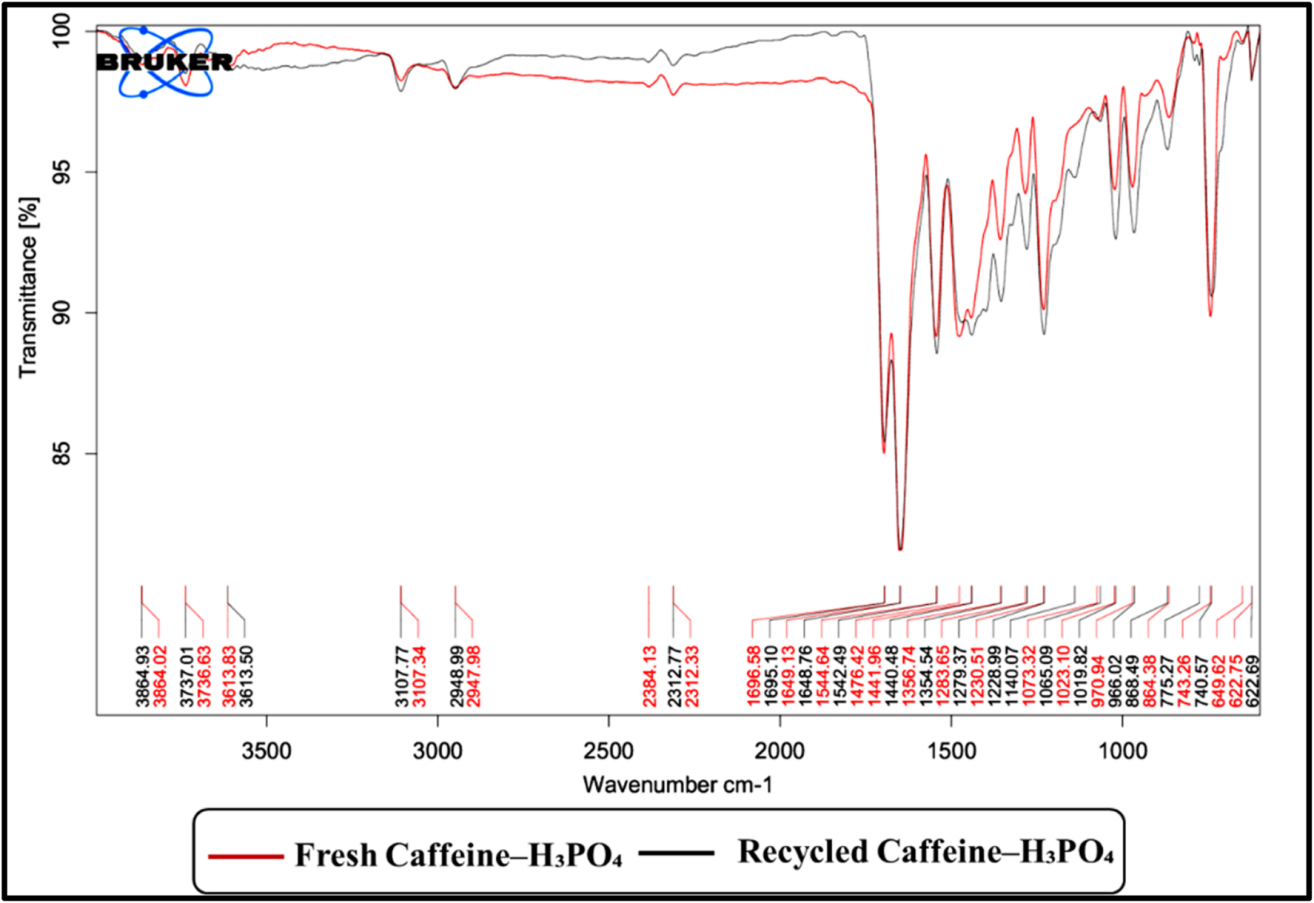
FT-IR analysis of the caffeine–H_3_PO_4_ catalyst in fresh and reused forms, confirming its structural stability and reusability.

Structural analysis of the recovered catalyst *via* FT-IR confirmed that its integrity remained largely unchanged after repeated use. The characteristic absorption bands were well retained, with no significant shifts or loss of intensity, indicating the preservation of key functional groups. The gradual decrease in yield observed after multiple catalytic cycles may be attributed to factors such as partial leaching of the catalyst, surface passivation, or minor structural reorganization, which can reduce active site availability over repeated use. This observation demonstrates the high stability and reusability of the catalyst under the applied reaction conditions (Fig. 19).

## Conclusions

Using recyclable, metal-free caffeine–H_3_PO_4_ as a green catalyst, this work offers an effective multicomponent method for the synthesis of pyrimido[4,5-*d*]pyrimidine derivatives. The protocol establishes itself as a sustainable substitute for conventional heterocyclic syntheses by operating under ethanolic circumstances, producing high yields in a brief reaction time, and requiring only straightforward work-up techniques. The catalyst was rigorously characterized using FT-IR, NMR, TGA-DTG analyses, XRD, and EDX, confirming its structural stability and retained catalytic activity. Its reusability over four consecutive cycles further underscores its operational robustness and efficiency. Environmental assessments using green chemistry metrics highlighted the eco-friendly nature of the method, with high atom economy, low E-factor, and minimal waste generation. The synthesized pyrimido[4,5-*d*]pyrimidine derivatives were subjected to preliminary biological evaluations and computational studies, which revealed promising antibacterial and cytotoxic potential. ADMET predictions indicated favorable absorption, balanced distribution, metabolic stability, and acceptable clearance, supporting the drug-likeness of the compounds. Overall, this work demonstrates not only the practical utility and recyclability of caffeine–H_3_PO_4_ as an effective catalyst but also the medicinal relevance of the resulting fused heterocycles. By integrating experimental synthesis, spectral characterization, eco-metric validation, biological testing, and *in silico* profiling, the study provides a comprehensive framework for developing pyrimido[4,5-*d*]pyrimidine derivatives as potential lead scaffolds in pharmaceutical research.

## Conflicts of interest

The authors declare no conflict of interest.

## Abbreviations

MCRs: Multicomponent reactions
FT-IR: Fourier transform-infrared
NMR: Nuclear magnetic resonance
TGA: Thermogravimetric analysis
AE: Atom economy
RME: Reaction mass efficiency
PMI: Process mass intensity
E-factor: Environmental factor
ADMET: Chemical absorption, distribution, metabolism, excretion, and toxicity
OCT 2: Organic cation transporter 2
CNS: Central nervous system
BBB: Blood brain barrier
hERG I: Human ether-a-go-go-related gene
Abs: Absorbed
NS: Non-substrate
NI: Non-inhibitor
Pen: Penetrant
NPen: Non-penetrant
P-gp: P-Glycoprotein
P-gp Substrate: P-Glycoprotein substrate
CYP: Cytochrome P450
CDK: Cyclin-dependent kinase
CP: Cyclophosphamide

## Supplementary Material

RA-016-D5RA10127A-s001

## Data Availability

The data supporting the findings of this study are included within the article and its supplementary information (SI). Additional data related to this work are available from the corresponding author upon reasonable request. Supplementary information is available. See DOI: https://doi.org/10.1039/d5ra10127a.
