# Supplementary material for: A green fabrication of pharmacologically relevant fused pyrimidines using recyclable caffeine–H3PO4 catalyst: insight into antibacterial and cytotoxic efficacy
Source: RSC Adv. 2026 Feb 13;16(10):8902–20. doi: 10.1039/d5ra10127a (PMC12903880; doi:10.1039/d5ra10127a)
Supplement: RA-016-D5RA10127A-s001 [file RA-016-D5RA10127A-s001.pdf]

Supplementary Information (SI) for RSC Advances.
This journal is © The Royal Society of Chemistry 2026

A Green Fabrication of Pharmacologically Relevant Fused Pyrimidines Using Recyclable Caffeine-H_3PO_4 Catalyst: Insight into Antibacterial and Cytotoxic Efficacy

Supplementary Information

Ayushi Bhatnagar[a], Nitish Rai[b], Namita Ashish Singh[c], Vidhi Jain[c] , Juhi Goyal[d] , and Gangotri Pemawat[a*]

[a]Department of Chemistry, University College of Science, Mohanlal Sukhadia University, Udaipur, Rajasthan, India 313001.

[b] Department of Biotechnology and Microbiology, University College of Science, Mohanlal Sukhadia University, Udaipur, Rajasthan, India 313001.

*Correspondence: drgpemawat@mlsu.ac.in

Table of Contents:

**1.**	**Experimental**	**2**
**2.**	**NMR and mass spectra of synthesized compounds**	**2-26**
4a	5-Phenyl-5,8-dihydropyrimido[4,5-*d*]pyrimidine-2,4,7(1*H*,3*H*,6*H*)-trione	
4b	5-(4-Methoxyphenyl)-5,8-dihydropyrimido[4,5-d]pyrimidine-2,4,7(1H,3H,6H)-trione	
4c	5-(4-Nitrophenyl)-5,8-dihydropyrimido[4,5-*d*]pyrimidine-2,4,7(1*H*,3*H*,6*H*)-trione	
4d	5-(4-Chlorophenyl)-5,8-dihydropyrimido[4,5-*d*]pyrimidine-2,4,7(1*H*,3*H*,6*H*)-trione	
4e	5-(3-Nitrophenyl)-5,8-dihydropyrimido[4,5-*d*]pyrimidine-2,4,7(1*H*,3*H*,6*H*)-trione	
4f	5-(4-Hydroxyphenyl)-5,8-dihydropyrimido[4,5-*d*]pyrimidine-2,4,7(1*H*,3*H*,6*H*)-trione	
4g	5-(3-Chlorophenyl)-5,8-dihydropyrimido[4,5-*d*]pyrimidine-2,4,7(1*H*,3*H*,6*H*)-trione	
4h	5-(2-Chlorophenyl)-5,8-dihydropyrimido[4,5-*d*]pyrimidine-2,4,7(1*H*,3*H*,6*H*)-trione	
4i	5-(4-(Dimethylamino)phenyl)-5,8-dihydropyrimido[4,5-*d*]pyrimidine-2,4,7(1*H*,3*H*,6*H*)-trione	
4j	5-(3-Hydroxy-4-methoxyphenyl)-5,8-dihydropyrimido[4,5-*d*]pyrimidine-	

	2,4,7(1*H*,3*H*,6*H*)-trione	
4k	5-(4-Hydroxy-3-methoxyphenyl)-5,8-dihydropyrimido[4,5-*d*]pyrimidine-2,4,7(1*H*,3*H*,6*H*)-trione	
4l	5-(4-(Benzyloxy)phenyl)-5,8-dihydropyrimido[4,5-*d*]pyrimidine-2,4,7(1*H*,3*H*,6*H*)-trione	
**3.**	**Green chemistry matrix**	**27**
**4.**	**Docking analysis**	**28**
**5.**	**Bioavailability radar plots**	**29**
**6.**	**Antiproliferative activity**	**30**
**7.**	**Antibacterial screening**	**30**
**8.**	**Reference**	**31**

1. Experimental:

The characterization and monitoring of the experimental procedures were carried out using standard analytical techniques. The synthesized catalyst was analyzed by FTIR spectroscopy using a Bruker instrument. Reaction progress was tracked by thin-layer chromatography (TLC) on silica gel plates, and visualization was done under 3NOS UV cabinets. Melting points were measured on an electric thermal melting point apparatus and are reported without correction. ^{1}H and ^{13}C NMR spectra were obtained on a JEOL 400 MHz spectrometer using $CDCl_3$ and DMSO-d_6 as solvents, with tetramethylsilane (TMS) as the internal reference. All reagents and chemicals were procured from Sigma-Aldrich, Loba-Chemie, and Merck, and were used as received, without further purification.

2. NMR and mass spectra of synthesized compounds:

4a: 5-Phenyl-5,8-dihydropyrimido[4,5-*d*]pyrimidine-2,4,7(1*H*,3*H*,6*H*)-trione

Off white solid, yield 93%, m.p.-276–278 ^{o}C, ^{1}H NMR (400 MHz, DMSO-d6) δ 9.33 (s, 1H, NH), 7.35 – 7.24 (m, 5H, Ar-H), 6.98 (s, 1H, NH), 6.61 (s, 1H, NH), 6.32 (s, 1H, NH), 5.41 (s, 1H, CH).13C NMR (101 MHz, DMSO-d_6) δ 158.43, 157.36, 154.92, 149.30, 140.75, 128.65, 128.65, 128.55, 128.45, 128.45, 93.03, 53.87. LC–MS (ESI^{+}): tR = 3.24 min; found = 258.49 Anal. % $C_{12}H_{10}N_4O_3$, C, 55.81; H, 3.90; N, 21.70; O, 18.59.

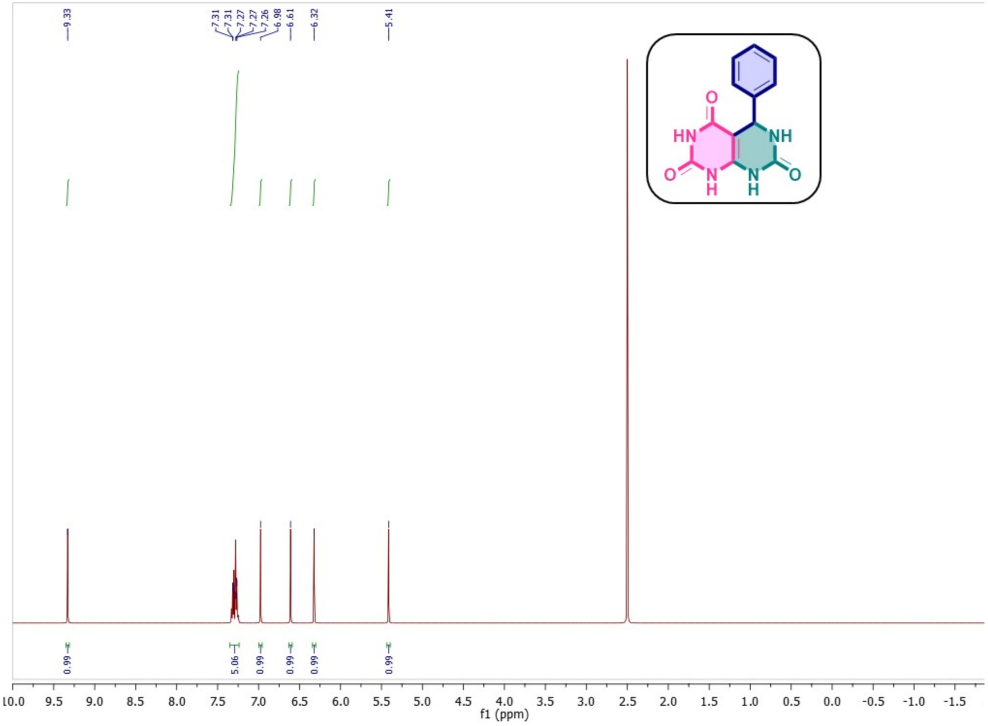

Figure 1: ^{1}H NMR spectrum of compound 4a

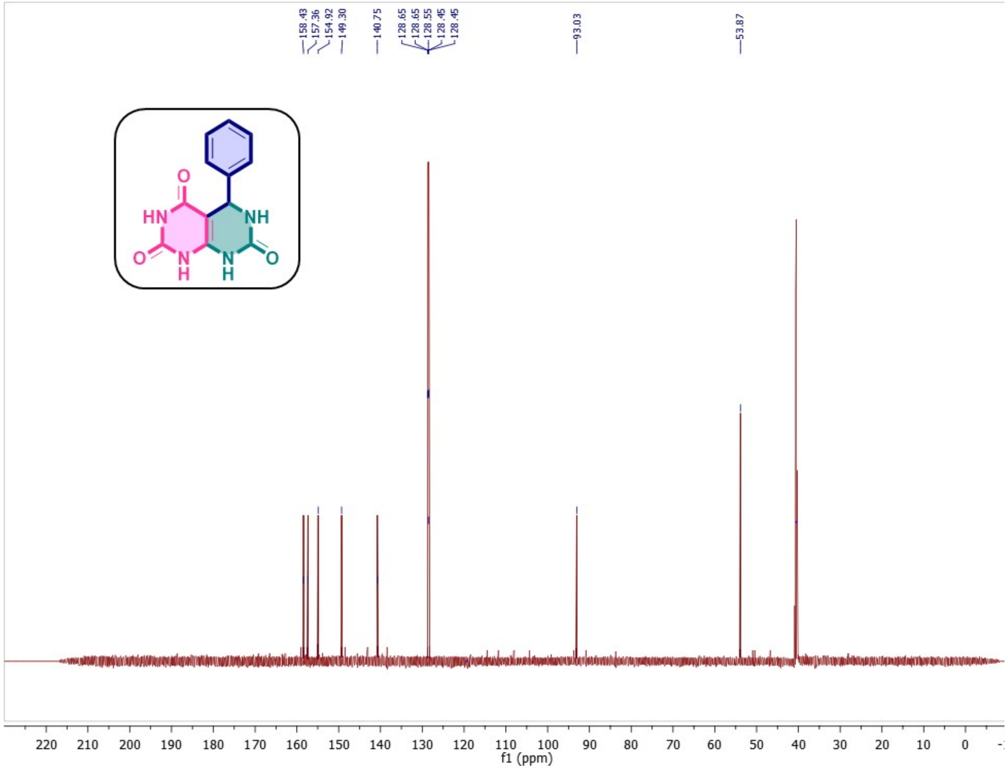

Figure 2: ^{13}C NMR spectrum of compound 4a

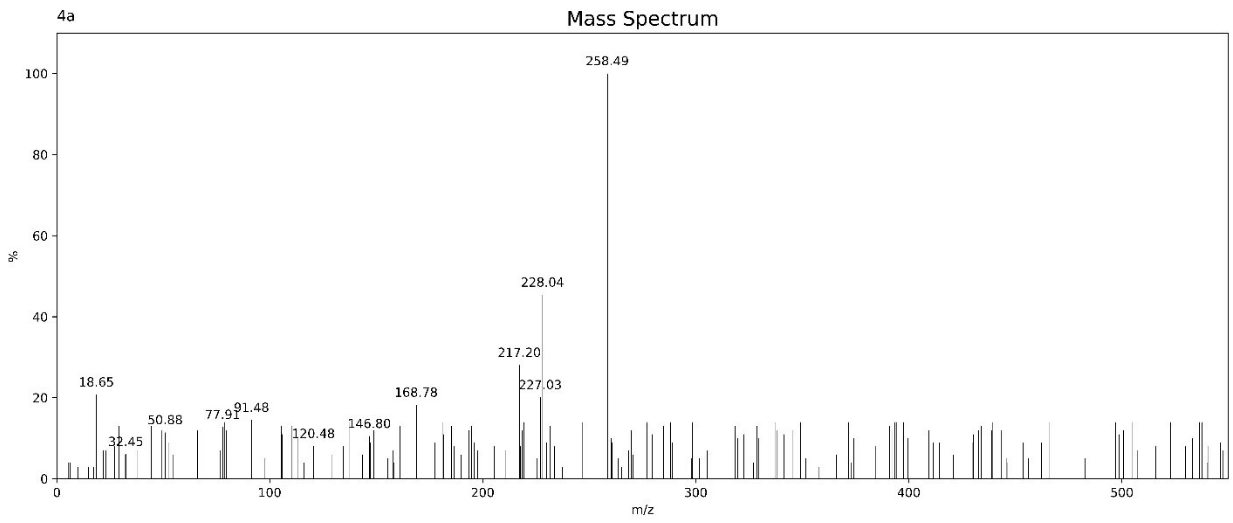

Figure 3: LC-MS spectrum of compound 4a

4b: 5-(4-Methoxyphenyl)-5,8-dihydropyrimido[4,5-*d*]pyrimidine-2,4,7(1*H*,3*H*,6*H*)-trione

Cream colored solid, yield 89%, m.p.-268–270 ^{o}C, ^{1}H NMR (400 MHz, DMSO-d6) δ 7.41 (s, 1H), 7.19 (d, *J* = 7.5 Hz, 2H), 6.96 (s, 1H), 6.90 (d, *J* = 7.5 Hz, 2H), 6.60 (s, 1H), 6.02 (s, 1H), 5.64 – 5.60 (m, 1H), 3.80 (s, 3H). 13C NMR (101 MHz, DMSO-d_6) δ 158.43, 157.36, 154.92, 149.30, 140.75, 128.65, 128.65, 128.55, 128.45, 128.45, 93.03, 53.87. LC–MS (ESI$^+$): tR = 2.13 min; found = 288.11 Anal. % $C_{13}H_{12}N_4O_4$, C, 54.17; H, 4.20; N, 19.44; O, 22.20

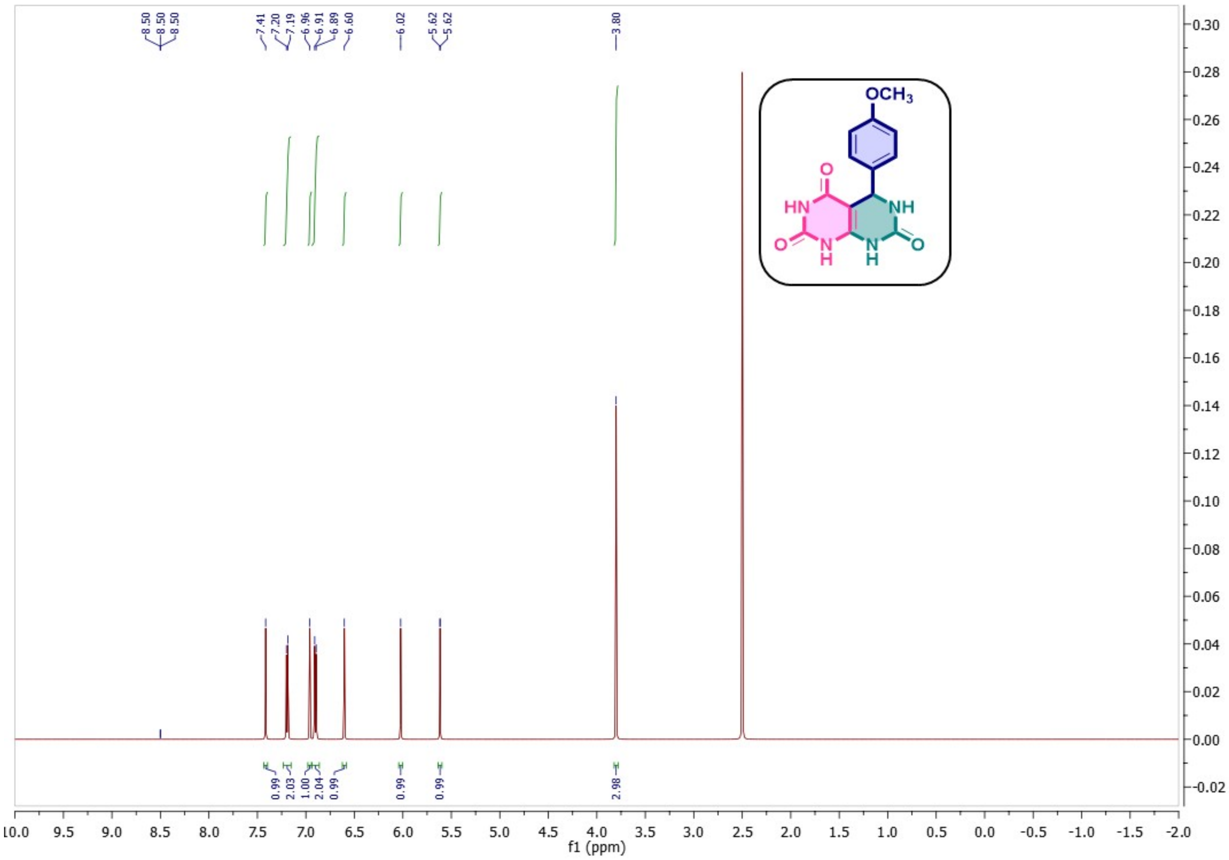

Figure 4: ^{1}H NMR spectrum of compound 4b

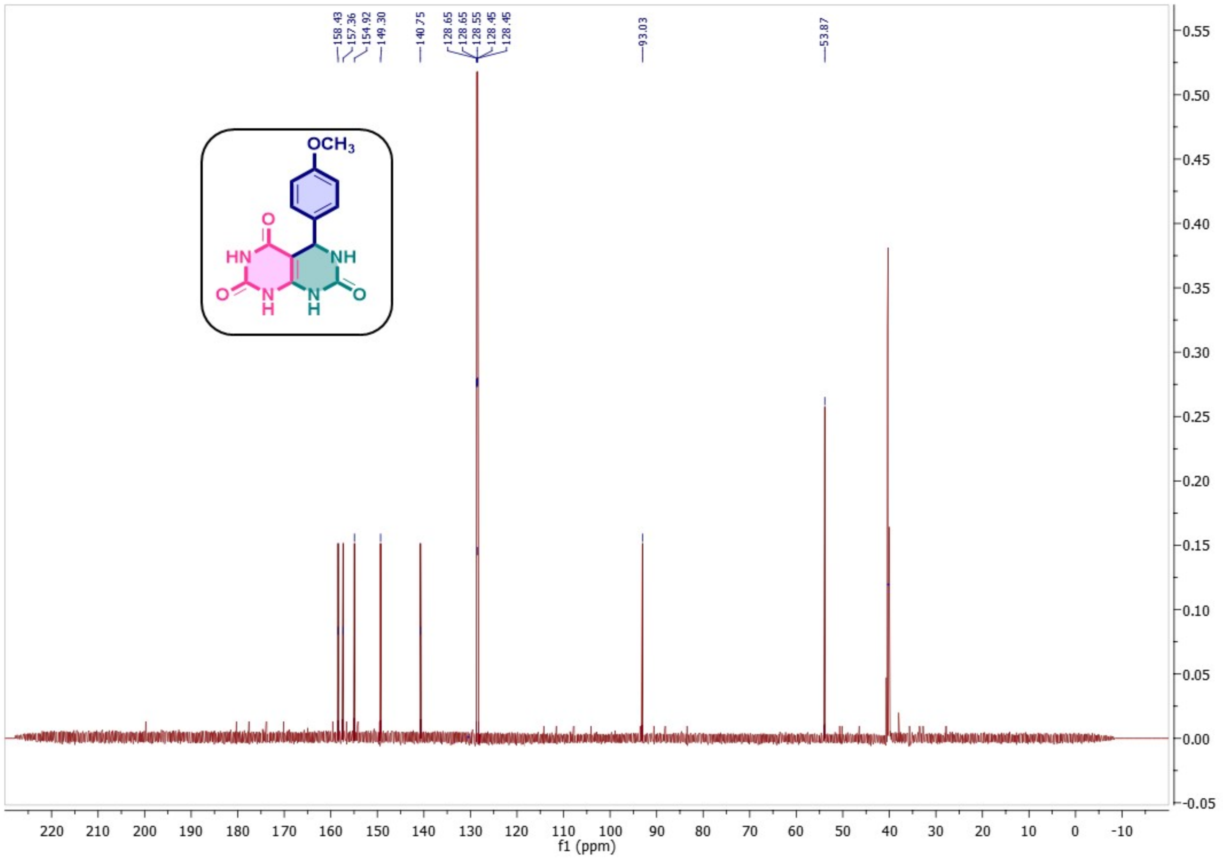

Figure 5: ^{13}C NMR spectrum of compound 4b

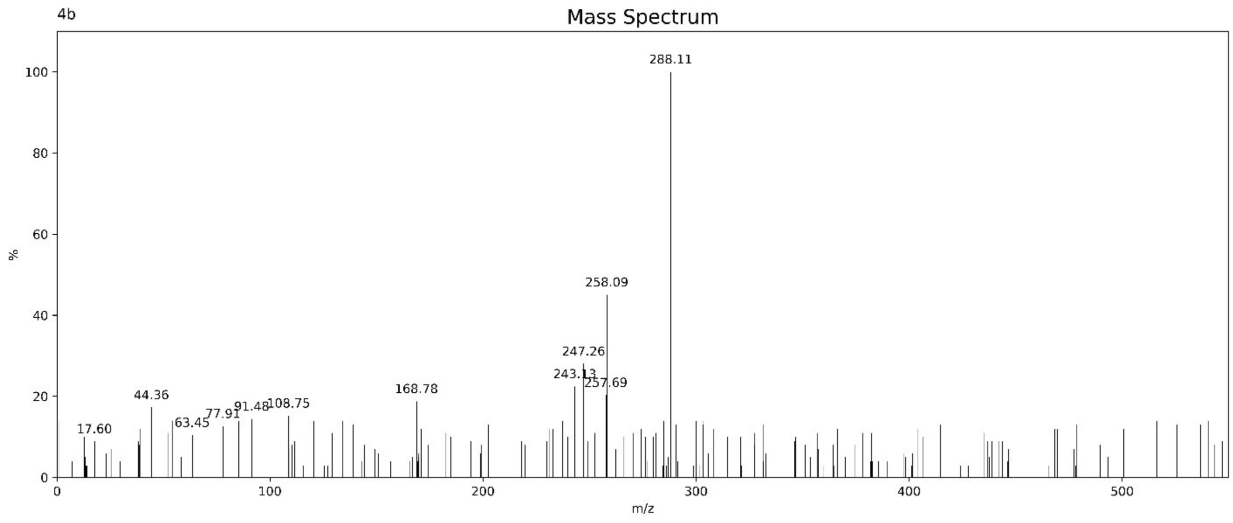

Figure 6: LC-MS spectrum of compound 4b

4c: 5-(4-Nitrophenyl)-5,8-dihydropyrimido[4,5-*d*]pyrimidine-2,4,7(1*H*,3*H*,6*H*)-trione

Yellow colored solid, yield 87%, m.p.-292–294 ^oC. ^1H NMR (400 MHz, DMSO-d6) δ 9.60 (s, 1H), 8.24 – 8.10 (m, 2H), 7.53 – 7.39 (m, 2H), 7.01 (d, J = 16.3 Hz, 2H), 6.04 (s, 1H), 5.32 (s, 1H). 13C NMR (101 MHz, DMSO-d_6) δ 158.43, 157.36, 154.92, 149.75, 149.30, 148.88, 128.10, 128.10, 123.25, 123.25, 93.03, 53.87. LC–MS (ESI^+): tR = 2.82 min; found = 303.18 Anal. % $C_{12}H_9N_5O_5$, C, 47.53; H, 2.99; N, 23.10; O, 26.38.

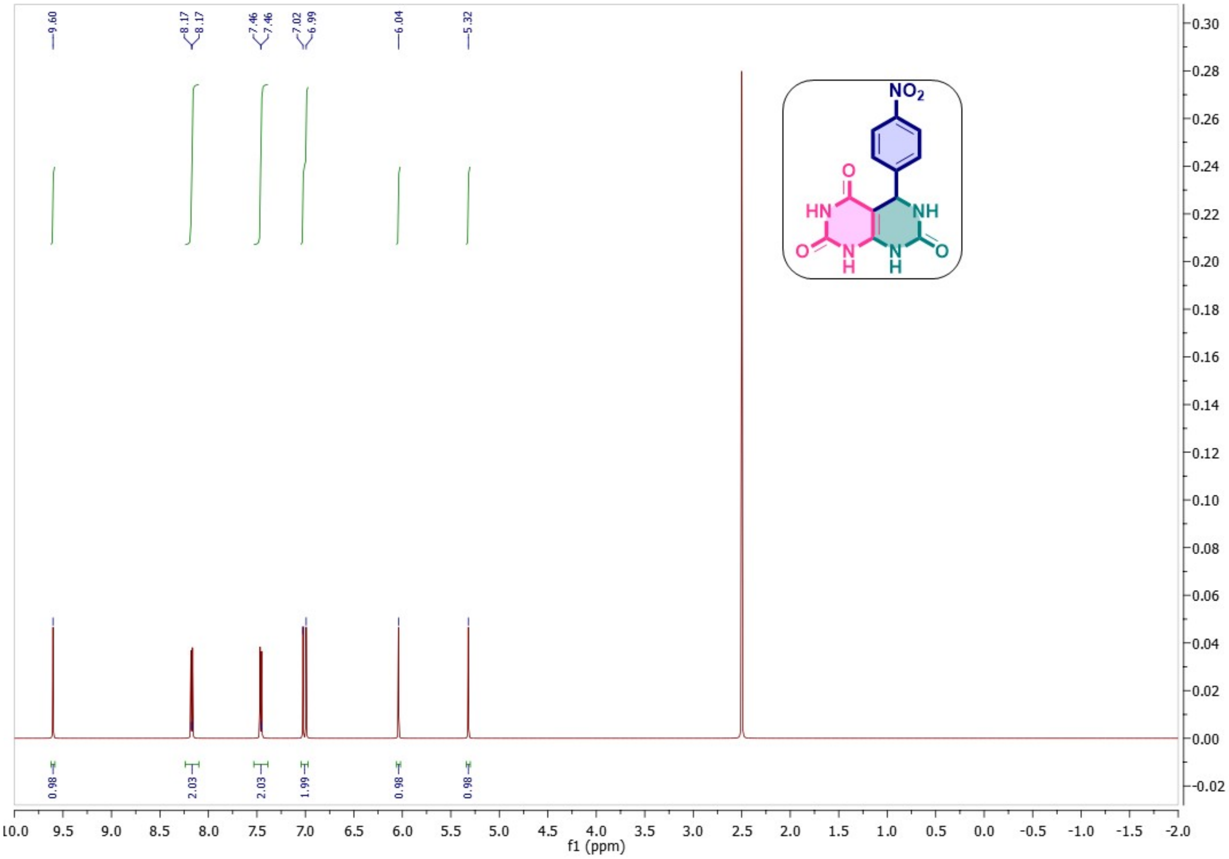

Figure 7: ^1H NMR spectrum of compound 4c

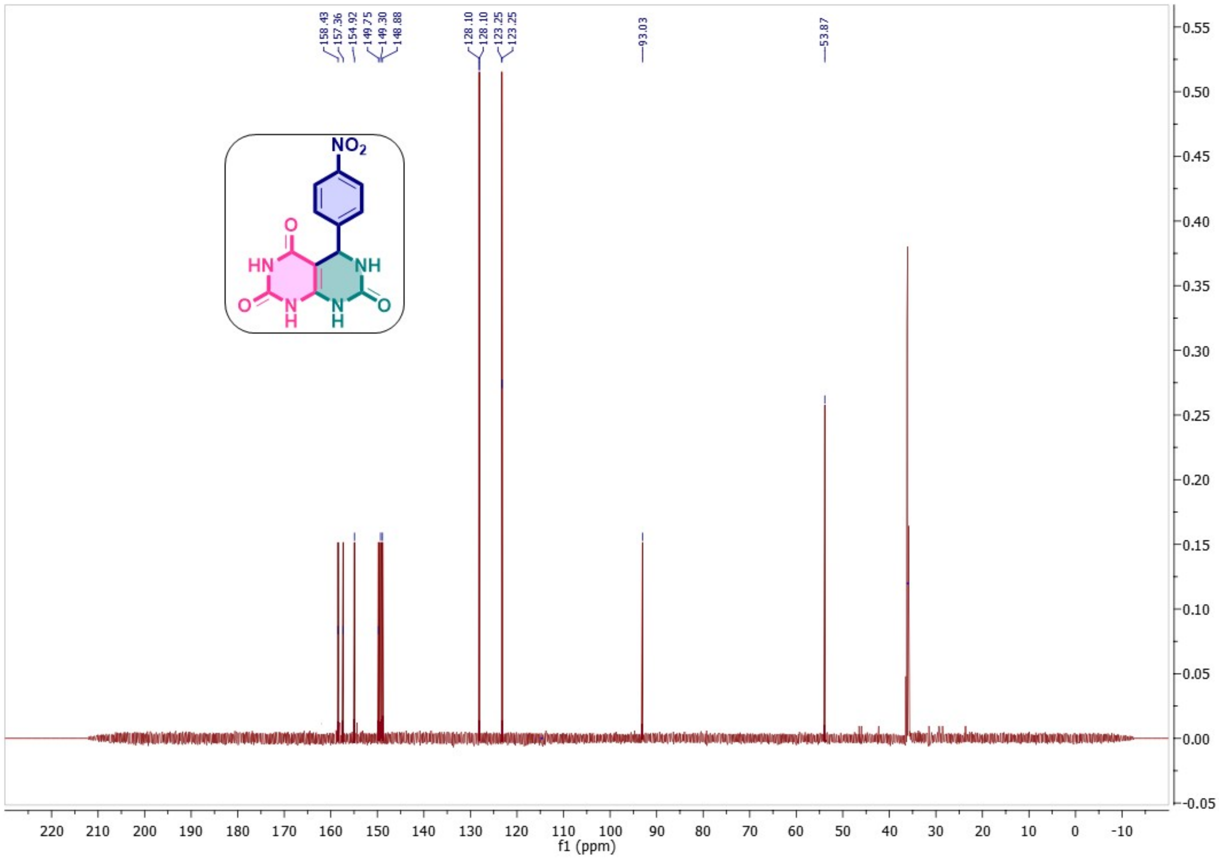

Figure 8: ^{13}C NMR spectrum of compound 4c

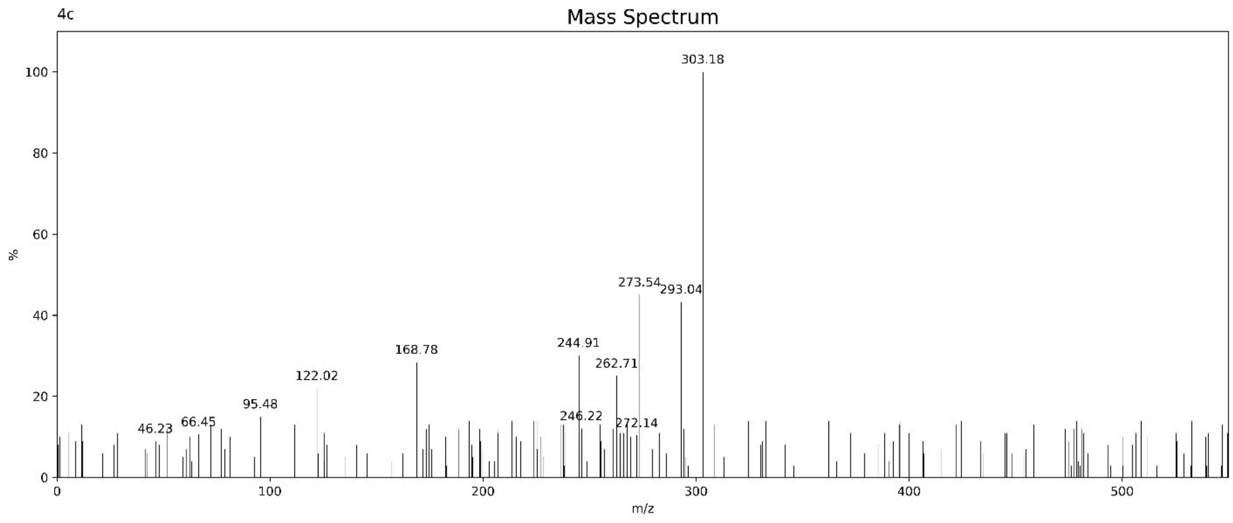

Figure 9: LC-MS spectrum of compound 4c

4d: 5-(4-Chlorophenyl)-5,8-dihydropyrimido[4,5-*d*]pyrimidine-2,4,7(1*H*,3*H*,6*H*)-trione

Pale yellow colored solid, yield 90%, m.p.- 280-282 °C, ^{1}H NMR (400 MHz, DMSO-d6) δ 8.30 (s, 1H), 8.03 – 7.89 (m, 2H), 7.52 – 7.38 (m, 2H), 6.96 (s, 1H), 6.58 (s, 1H), 6.12 (s, 1H), 5.57 (s, 1H). 13C NMR (101 MHz, DMSO-d6) δ 158.43, 157.36, 154.92, 149.30, 138.86, 133.48, 130.63, 130.63, 128.55, 128.55, 93.03, 53.87. LC–MS (ESI$^+$): tR = 3.22 min; found = 292.09 Anal. % $C_{12}H_9ClN_4O_3$, C, 49.25; H, 3.10; Cl, 12.11; N, 19.14; O, 16.40.

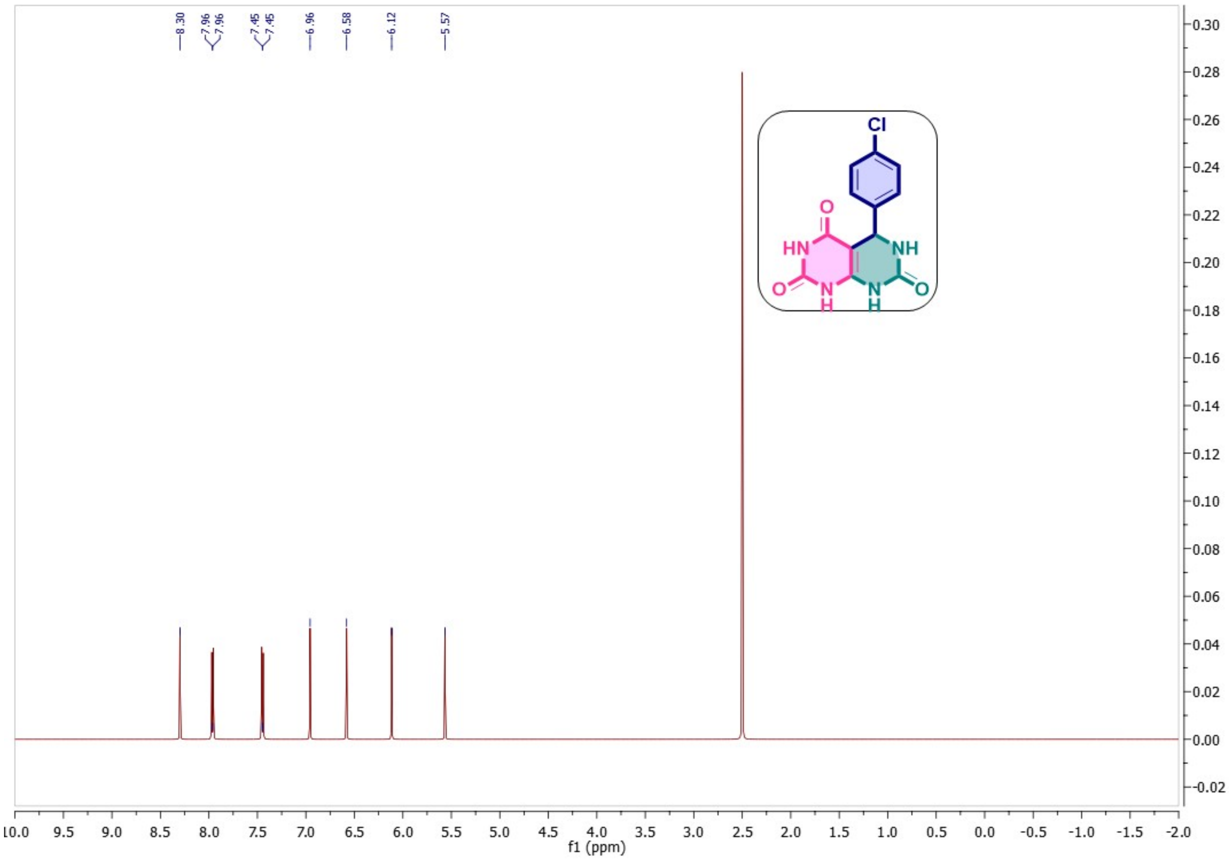

Figure 10: ^{1}H NMR spectrum of compound 4d

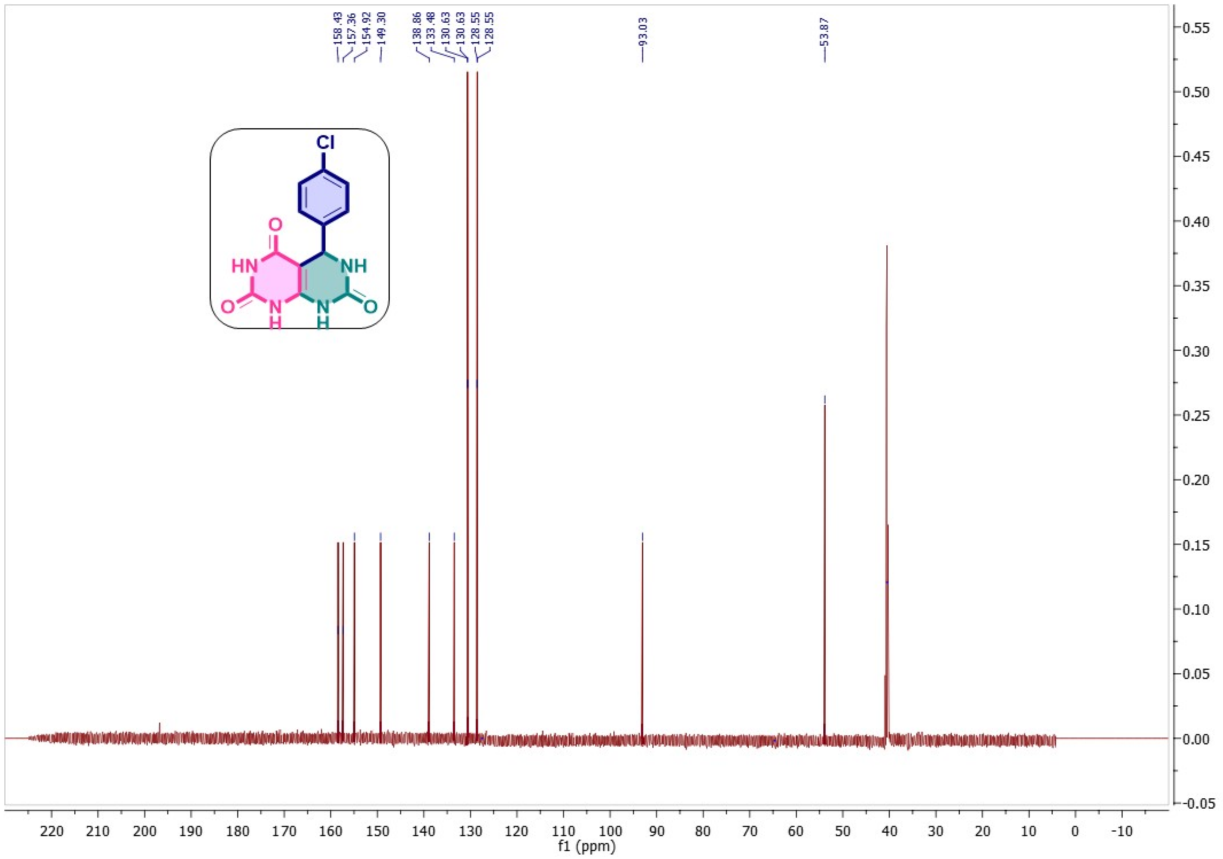

Figure 11: ^{13}C NMR spectrum of compound 4d

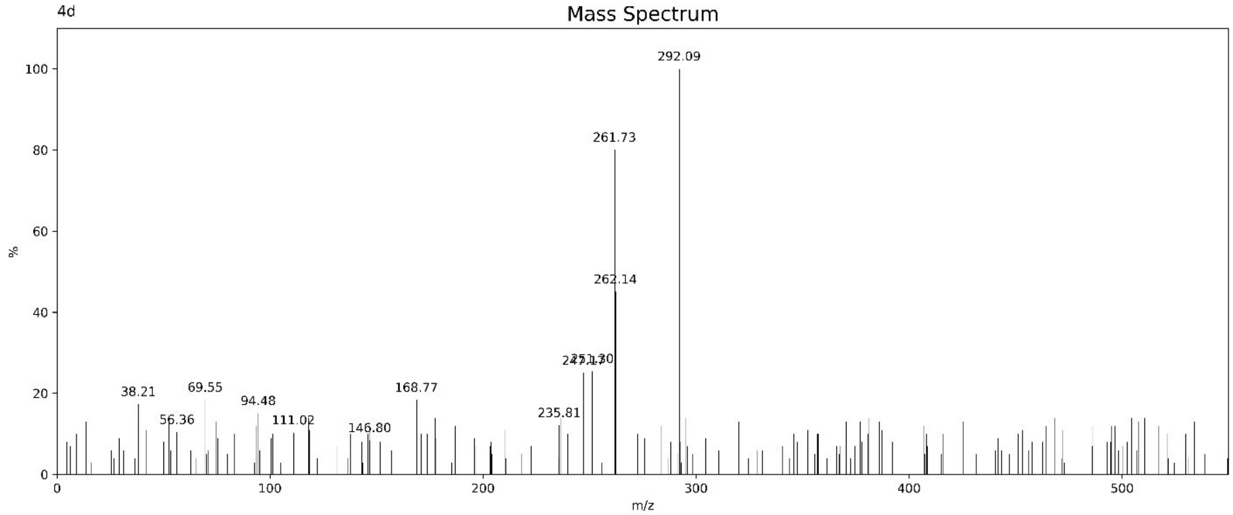

Figure 12: LC-MS spectrum of compound 4d

4e: 5-(3-Nitrophenyl)-5,8-dihydropyrimido[4,5-*d*]pyrimidine-2,4,7(1*H*,3*H*,6*H*)-trione

Straw yellow colored solid, yield 88%, m.p.- 288-290 ^{o}C. ^{1}H NMR (400 MHz, DMSO-d6) δ 8.32 (s, 1H), 8.09 (s, 1H), 7.49 (s, 1H), 7.35 (s, 1H), 7.19 (s, 1H), 6.95 (s, 1H), 6.63 (s, 1H), 6.18 (s, 1H), 5.64 (s, 1H). 13C NMR (101 MHz, DMSO-d6) δ 158.43, 157.36, 154.92, 149.30, 148.57, 141.97, 135.57, 130.19, 127.19, 126.66, 93.03, 53.88. LC–MS (ESI^{+}): tR = 3.25 min; found = 292.20 Anal. % $C_{12}H_9N_5O_5$, C, 47.53; H, 2.99; N, 23.10; O, 26.38.

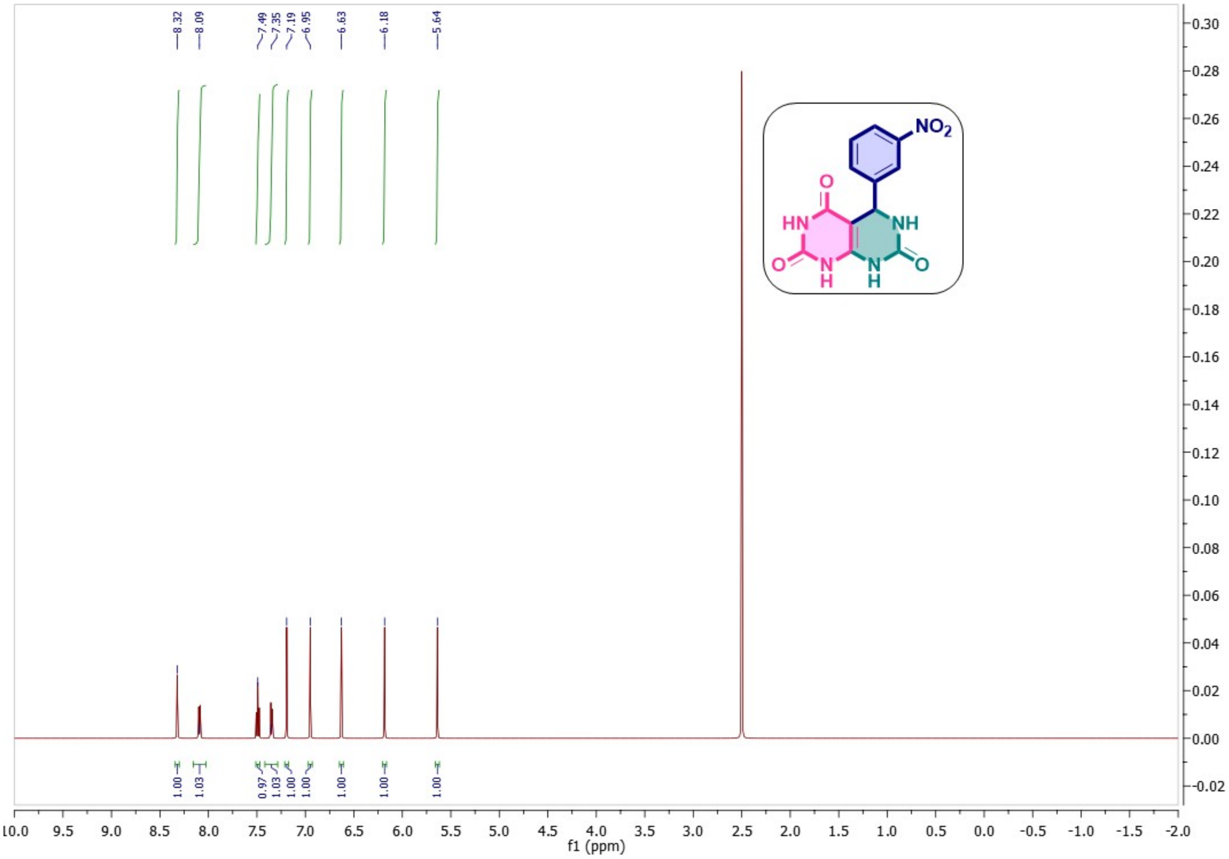

Figure 13: ^{1}H NMR spectrum of compound 4e

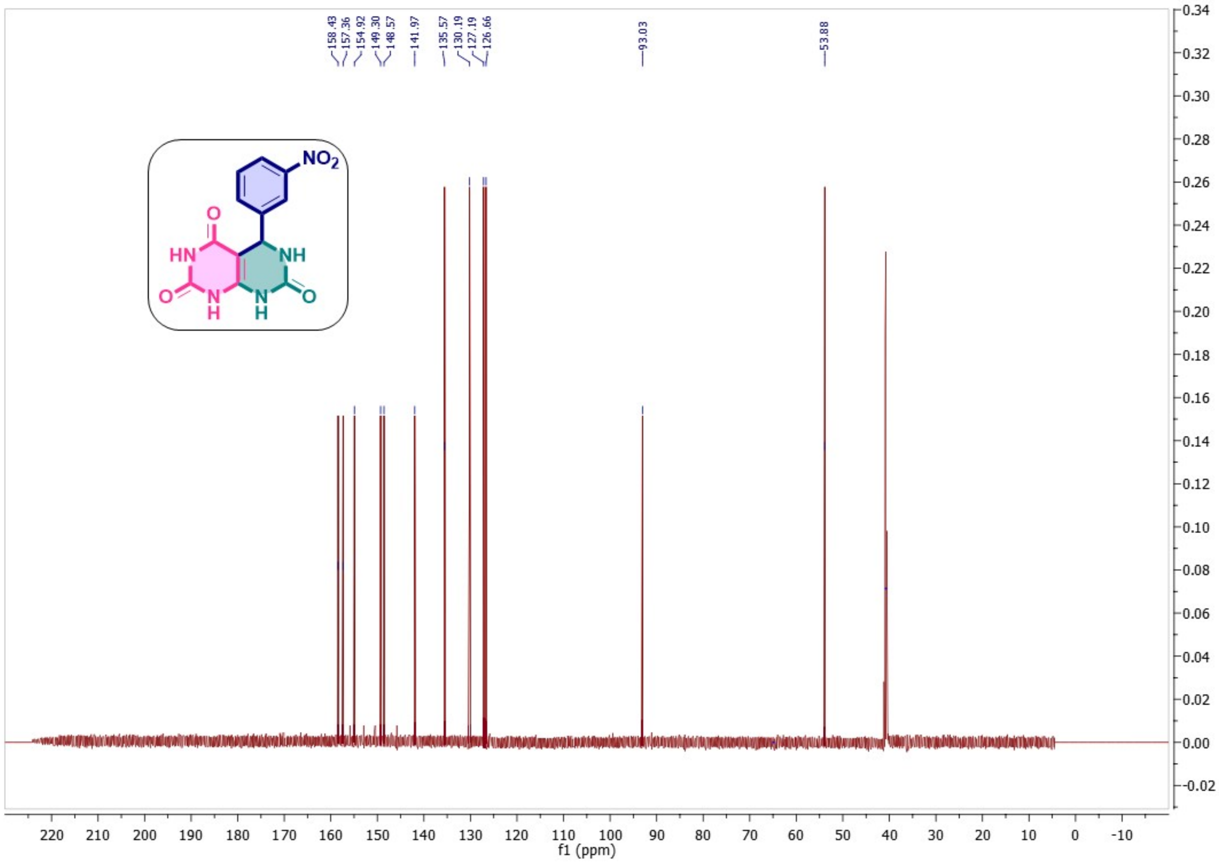

Figure 14: ^{13}C NMR spectrum of compound 4e

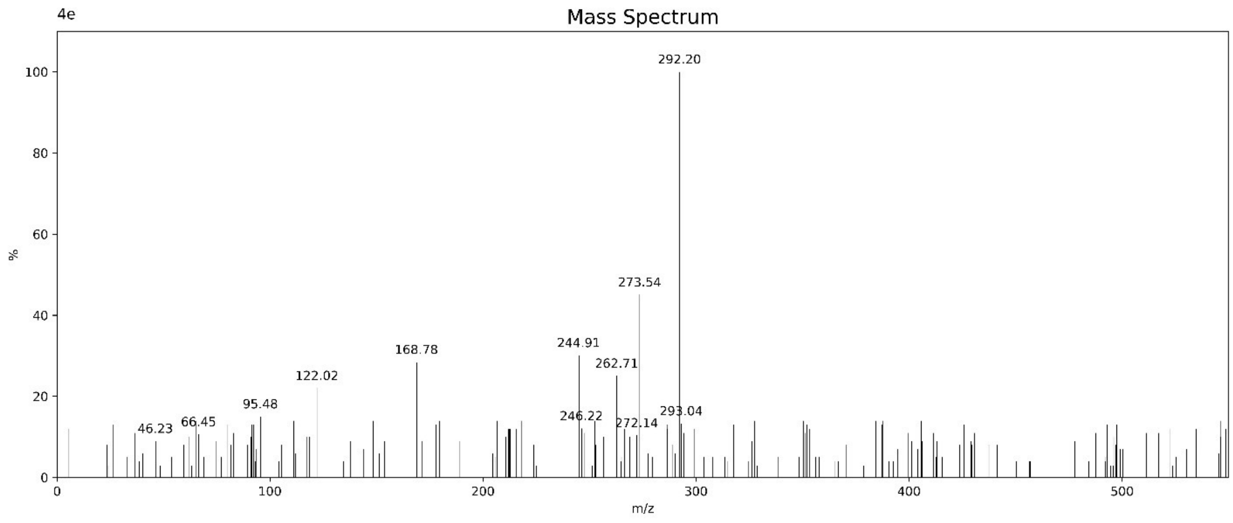

Figure 15: LC-MS spectrum of compound 4e

4f: 5-(4-Hydroxyphenyl)-5,8-dihydropyrimido[4,5-*d*]pyrimidine-2,4,7(1*H*,3*H*,6*H*)-trione

Cream white colored solid, yield 88%, m.p.- 275-277 °C. ^{1}H NMR (400 MHz, DMSO-d6) δ 7.87 (s, 1H), 7.14 – 7.01 (m, 2H), 6.99 (s, 1H), 6.85 – 6.70 (m, 2H), 6.61 (s, 1H), 6.07 (s, 1H), 5.61 (s, 1H), 3.88 (s, 1H). 13C NMR (101 MHz, DMSO-d6) δ 158.43, 157.63, 157.36, 154.92, 149.30, 131.30, 129.80, 129.80, 115.59, 115.59, 93.03, 53.87. LC–MS (ESI^{+}): tR = 2.13 min; found = 274.17 Anal. % $C_{12}H_{10}N_4O_4$, C, 52.56; H, 3.68; N, 20.43; O, 23.34.

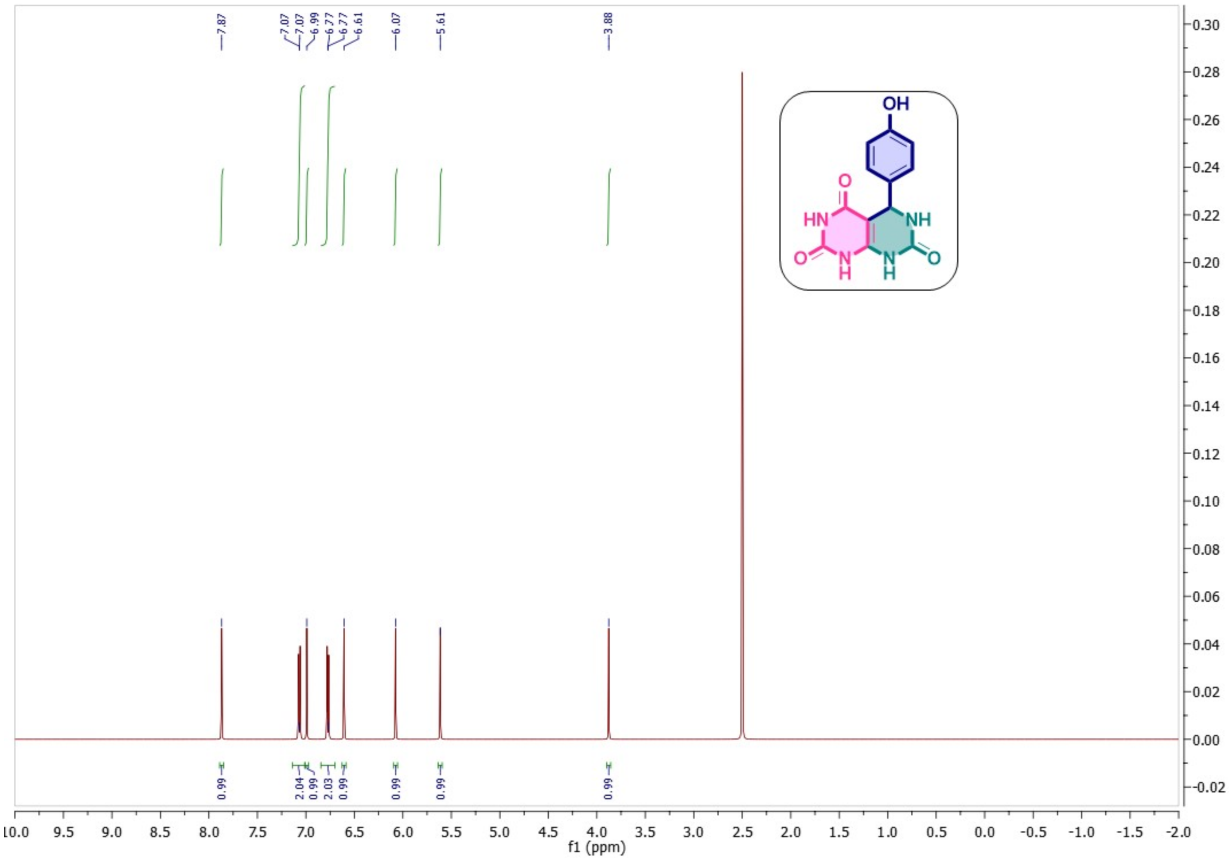

Figure 16: ^{1}H NMR spectrum of compound 4f

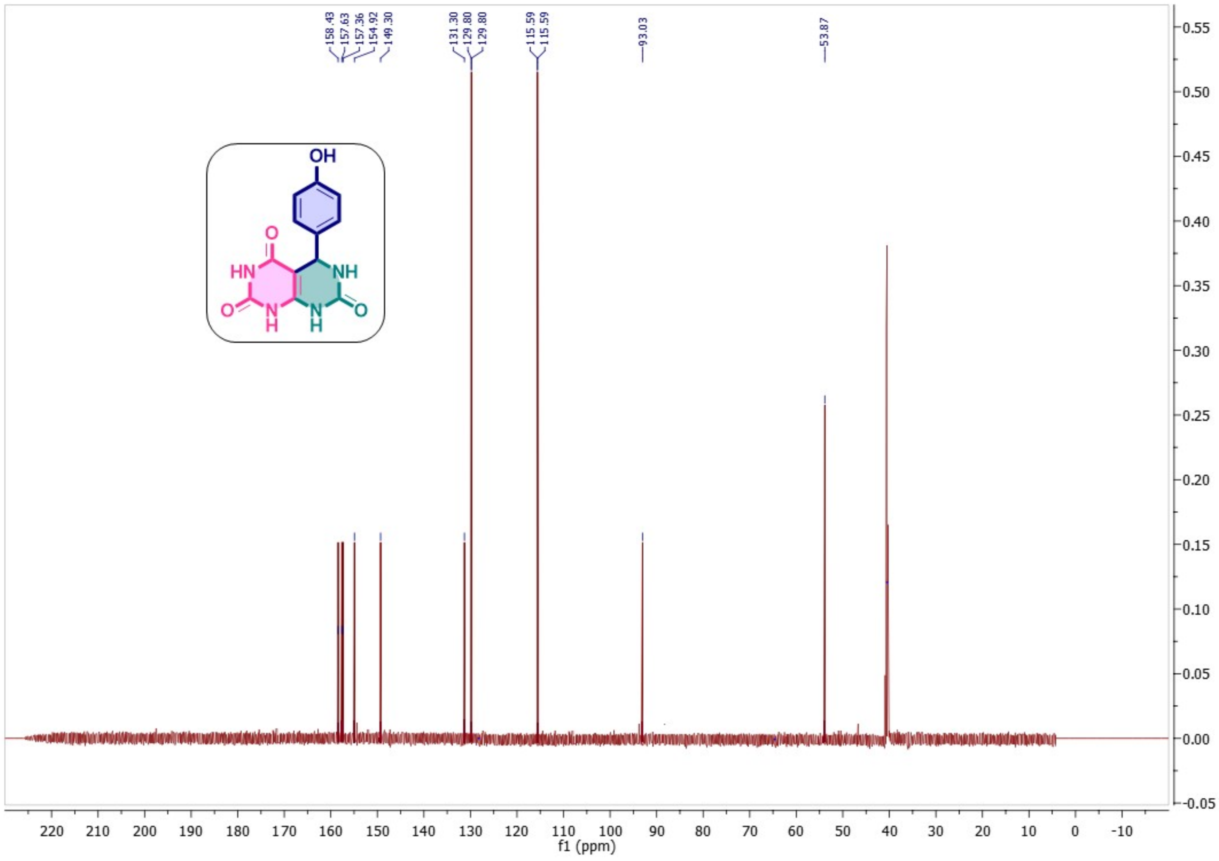

Figure 17: ^{13}C NMR spectrum of compound 4f

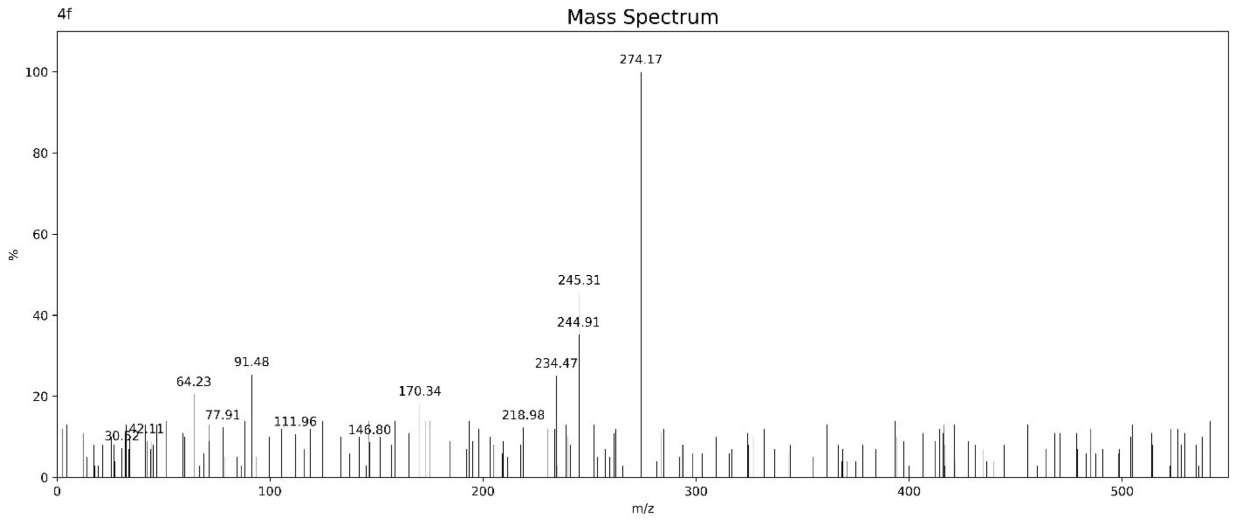

Figure 18: LC-MS spectrum of compound 4f

4g: 5-(3-Chlorophenyl)-5,8-dihydropyrimido[4,5-*d*]pyrimidine-2,4,7(1*H*,3*H*,6*H*)-trione

Greyish white colored solid, yield 90%, m.p.- 283-285 °C.^{1}H NMR (400 MHz, DMSO-d6) δ 7.82 (s, 1H), 7.36 (s, 1H), 7.25 (d, *J* = 2.0 Hz, 2H), 7.06 (s, 1H), 7.00 (s, 1H), 6.61 (s, 1H), 6.13 (s, 1H), 5.61 (s, 1H). 13C NMR (101 MHz, DMSO-d6) δ 158.43, 157.36, 154.92, 149.30, 143.55, 133.41, 128.94, 128.69, 127.65, 126.92, 93.03, 53.88. LC–MS (ESI$^+$): tR = 3.38 min; found = 292.10 Anal. % $C_{12}H_9ClN_4O_3$, C, 49.25; H, 3.10; Cl, 12.11; N, 19.14; O, 16.40.

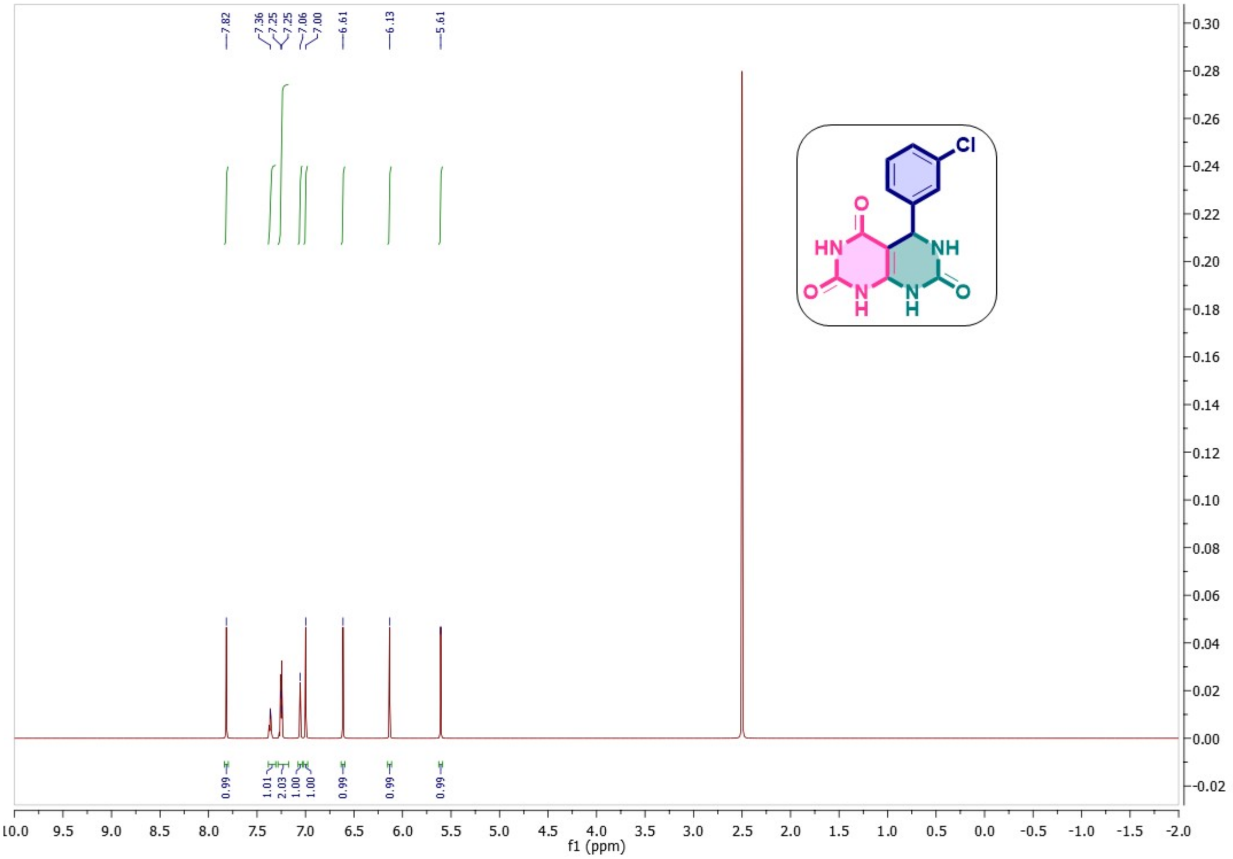

Figure 19: ^{1}H NMR spectrum of compound 4g

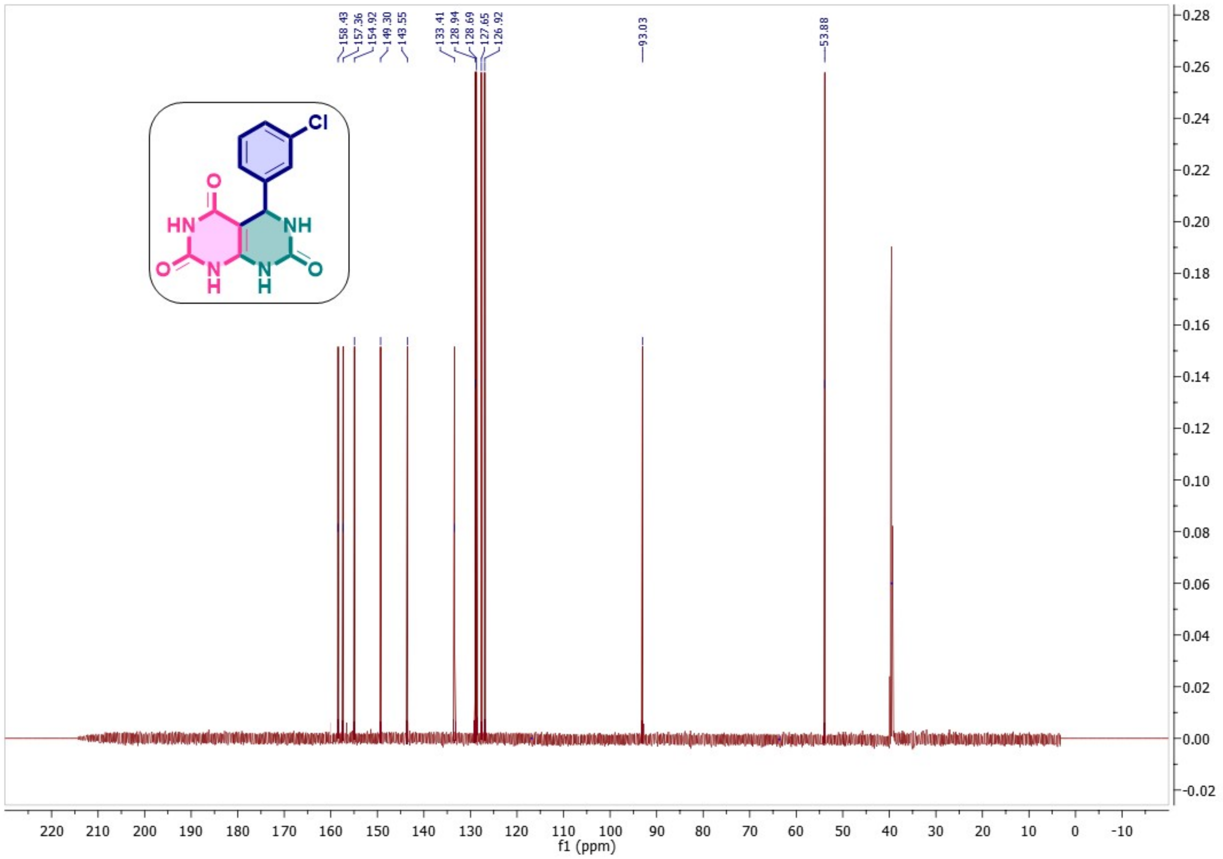

Figure 20: ^{13}C NMR spectrum of compound 4g

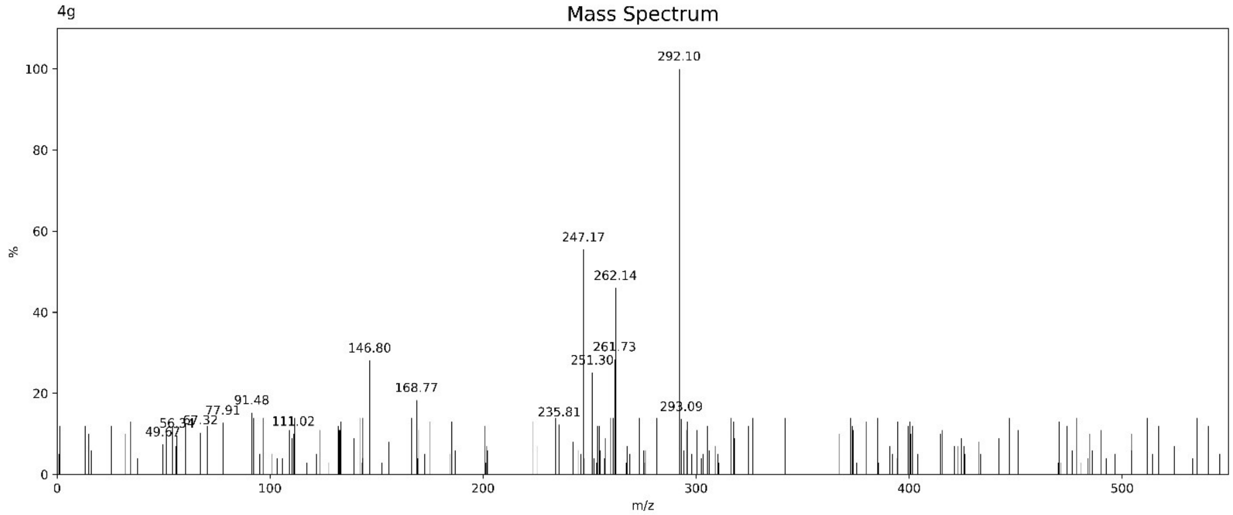

Figure 21: LC-MS spectrum of compound 4g

4h: 5-(2-Chlorophenyl)-5,8-dihydropyrimido[4,5-*d*]pyrimidine-2,4,7(1*H*,3*H*,6*H*)-trione

Off white colored solid, yield 88%, m.p.- 279-281 °C. ^{1}H NMR (400 MHz, DMSO-d6) δ 9.02 (s, 1H), 7.39 (s, 1H), 7.30 (s, 1H), 7.25 (s, 1H), 7.21 (s, 1H), 7.02 (s, 1H), 6.64 (s, 1H), 6.18 (s, 1H), 5.40 (s, 1H). 13C NMR (101 MHz, DMSO-d6) δ 158.43, 157.36, 154.92, 149.36, 139.91, 134.11, 131.80, 130.22, 129.36, 126.76, 91.54, 53.00. LC–MS (ESI$^+$): tR = 3.23 min; found = 292.02 Anal. % $C_{12}H_9ClN_4O_3$, C, 49.25; H, 3.10; Cl, 12.11; N, 19.14; O, 16.40.

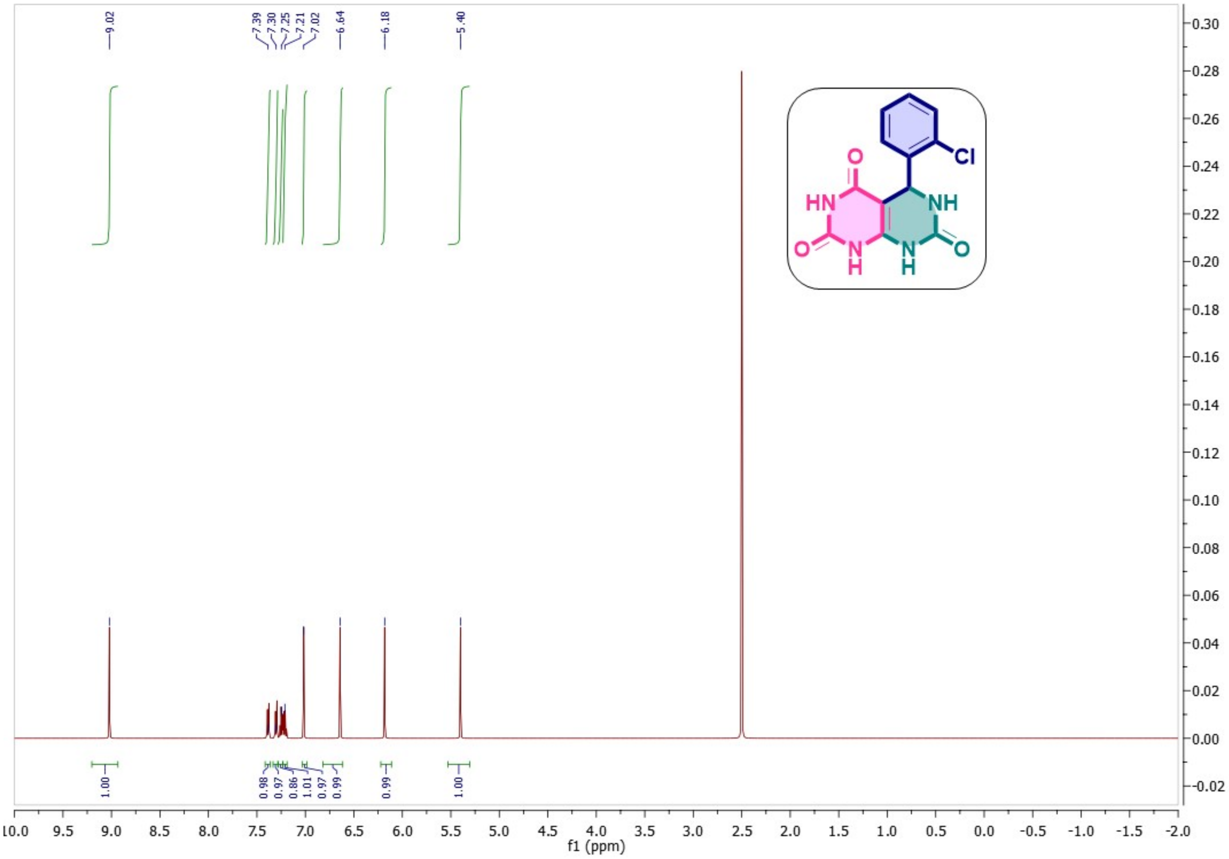

Figure 22: ^{1}H NMR spectrum of compound 4h

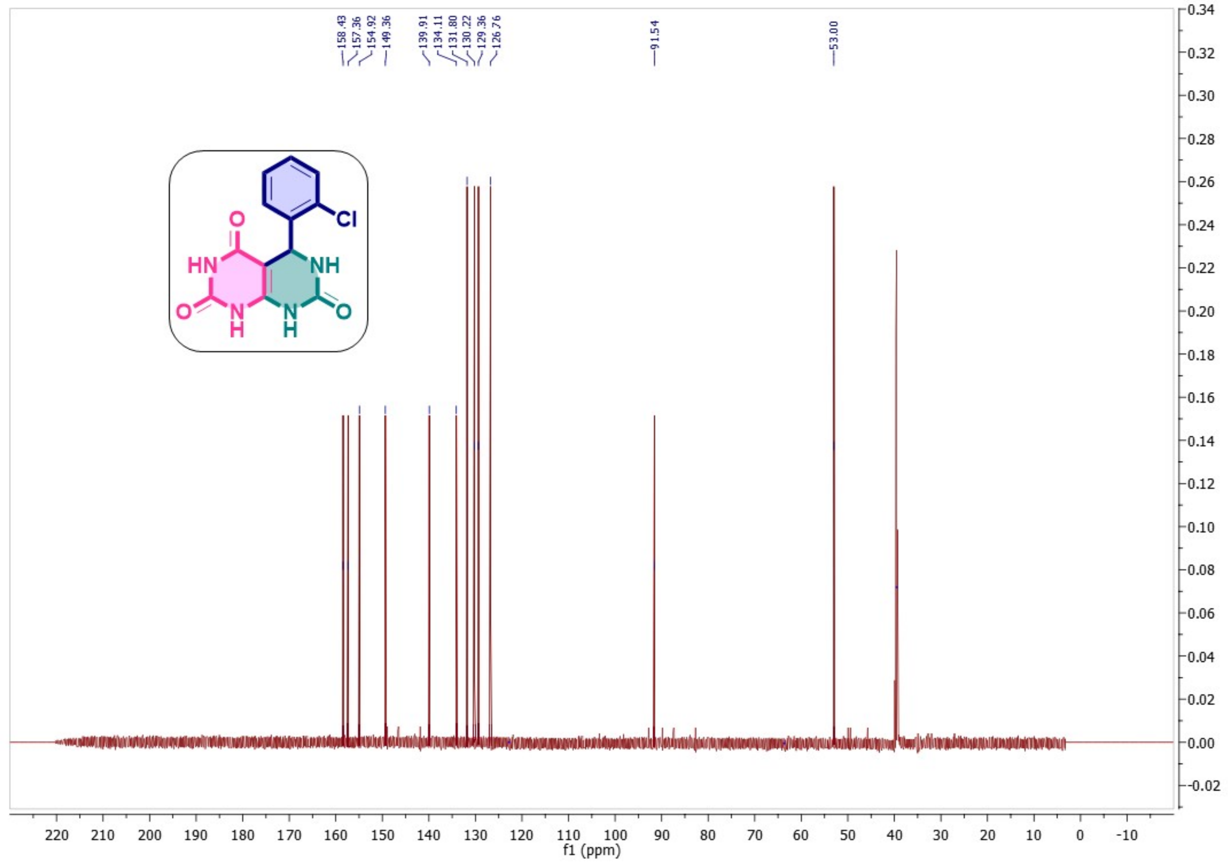

Figure 23: ^{13}C NMR spectrum of compound 4h

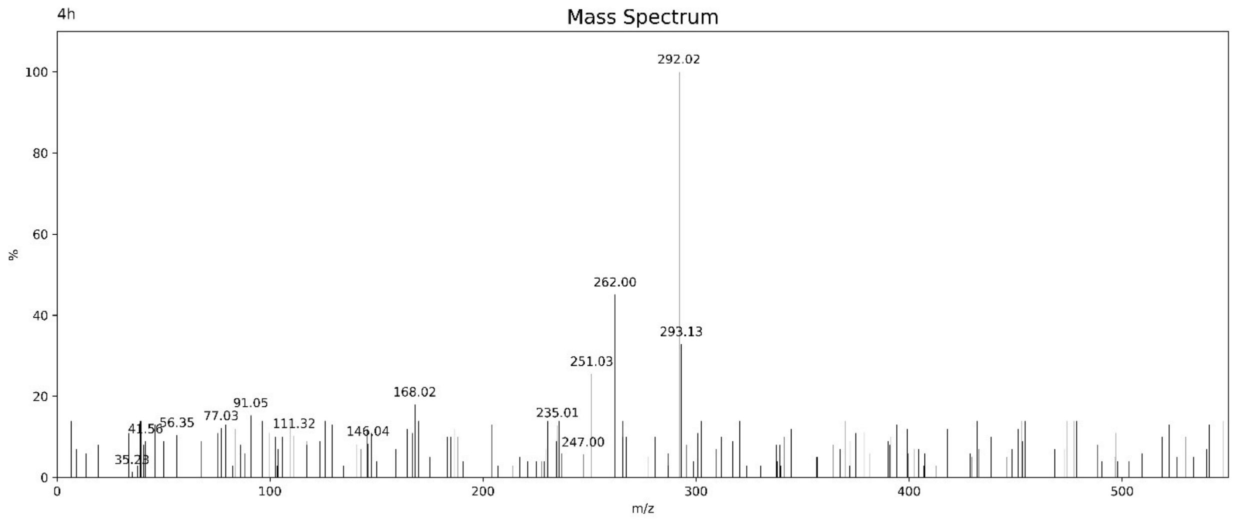

Figure 24: LC-MS spectrum of compound 4h

4i: 5-(4-(Dimethylamino)phenyl)-5,8-dihydropyrimido[4,5-*d*]pyrimidine-2,4,7(1*H*,3*H*,6*H*)-trione

Brownish yellow colored solid, yield 92%, m.p.- 264-266 ^{o}C. ^{1}H NMR (400 MHz, DMSO-d6) δ 9.16 (s, 1H), 7.15 – 7.01 (m, 2H), 6.97 (s, 1H), 6.72 – 6.59 (m, 3H), 6.20 (s, 1H), 5.41 (s, 1H), 2.91 (s, 6H).13C NMR (101 MHz, DMSO-d6) δ 158.43, 157.36, 154.92, 151.82, 149.30, 129.64, 127.85, 127.85, 112.53, 112.53, 93.03, 53.87, 41.91, 41.91. LC–MS (ESI^{+}): tR = 3.64 min; found = 301.12 Anal. % $C_{14}H_{15}N_5O_3$, C, 55.81; H, 5.02; N, 23.24; O, 15.93.

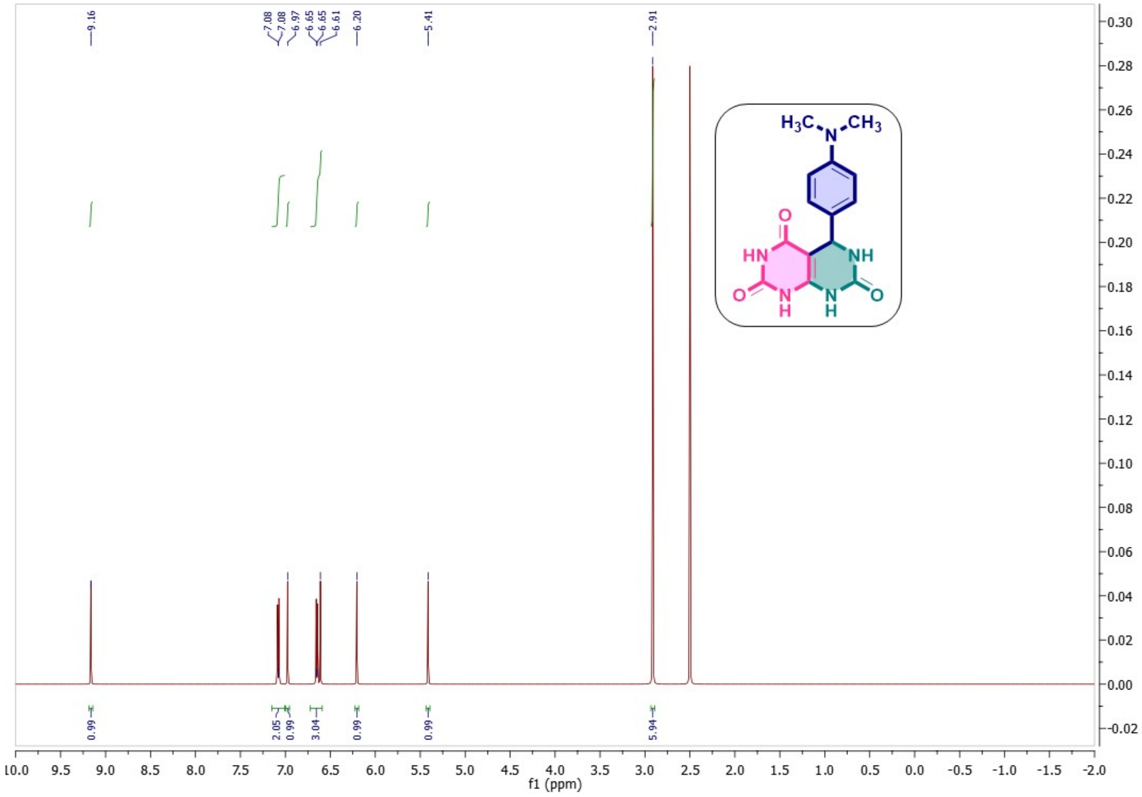

Figure 25: ^{1}H NMR spectrum of compound 4i

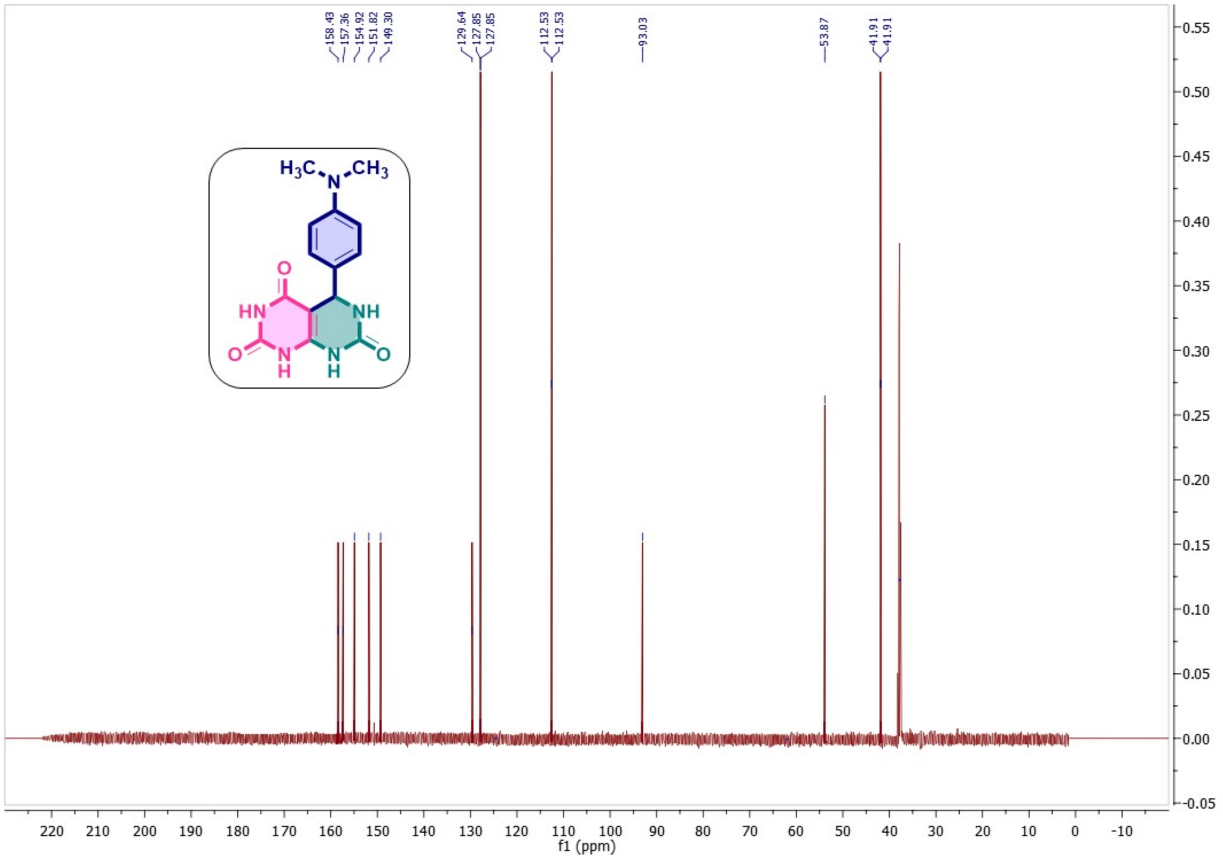

Figure 26: ^{13}C NMR spectrum of compound 4i

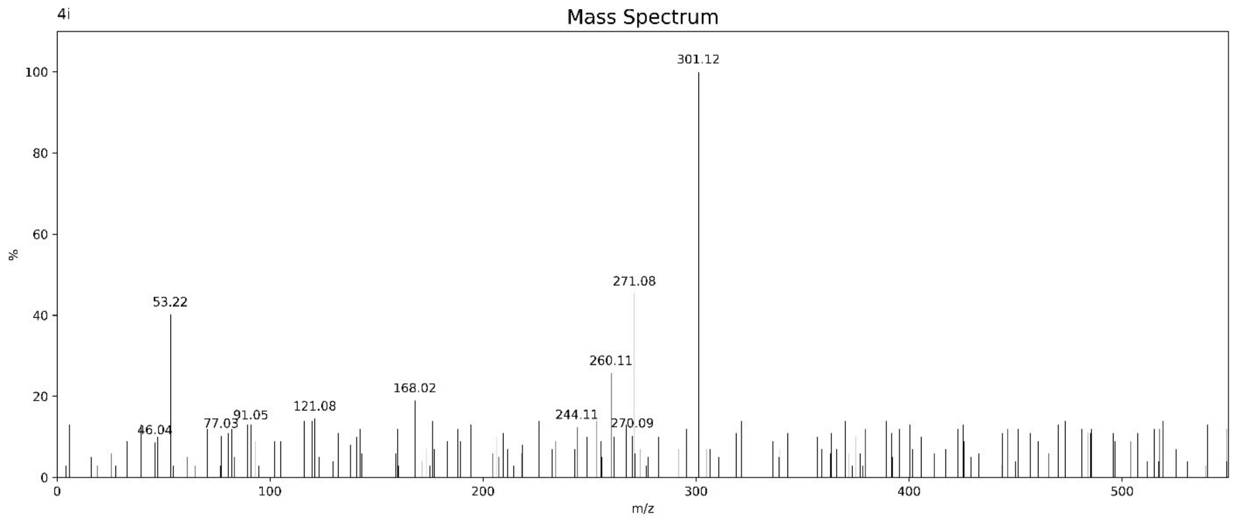

Figure 27: LC-MS spectrum of compound 4i

4j: 5-(3-Hydroxy-4-methoxyphenyl)-5,8-dihydropyrimido[4,5-*d*]pyrimidine-2,4,7(1*H*,3*H*,6*H*)-trione

Pale cream solid, yield 90%, m.p.- 272-274 °C, ^{1}H NMR (400 MHz, DMSO-d6) δ 6.96 (s, 1H), 6.83 (s, 1H), 6.78 – 6.67 (m, 3H), 6.32 (s, 1H), 6.23 (s, 1H), 5.38 (s, 1H), 4.01 (s, 1H), 3.79 (s, 3H). 13C NMR (101 MHz, DMSO-d6) δ 158.43, 157.36, 154.92, 149.30, 147.11, 145.84, 131.12, 119.25, 116.22, 114.09, 93.03, 56.79, 53.88. LC–MS (ESI^{+}): tR = 3.60 min; found = 304.14 Anal. % $C_{13}H_{12}N_4O_5$, C, 51.32; H, 3.98; N, 18.41; O, 26.29.

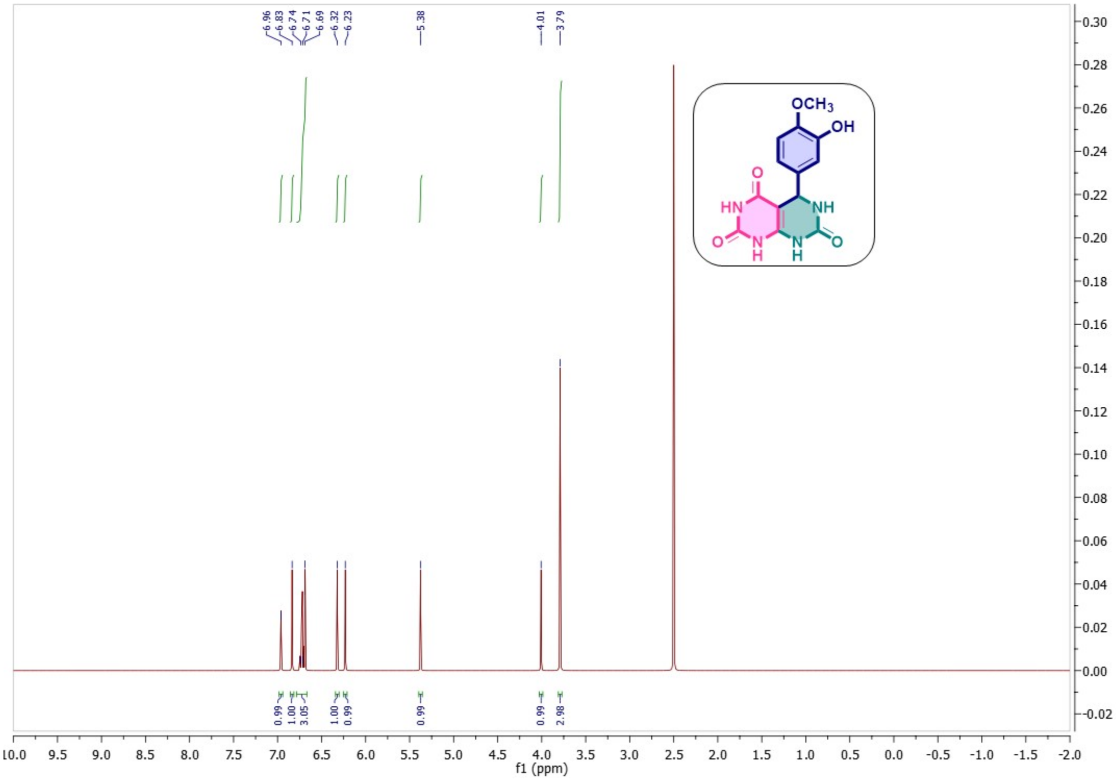

Figure 28: ^{1}H NMR spectrum of compound 4j

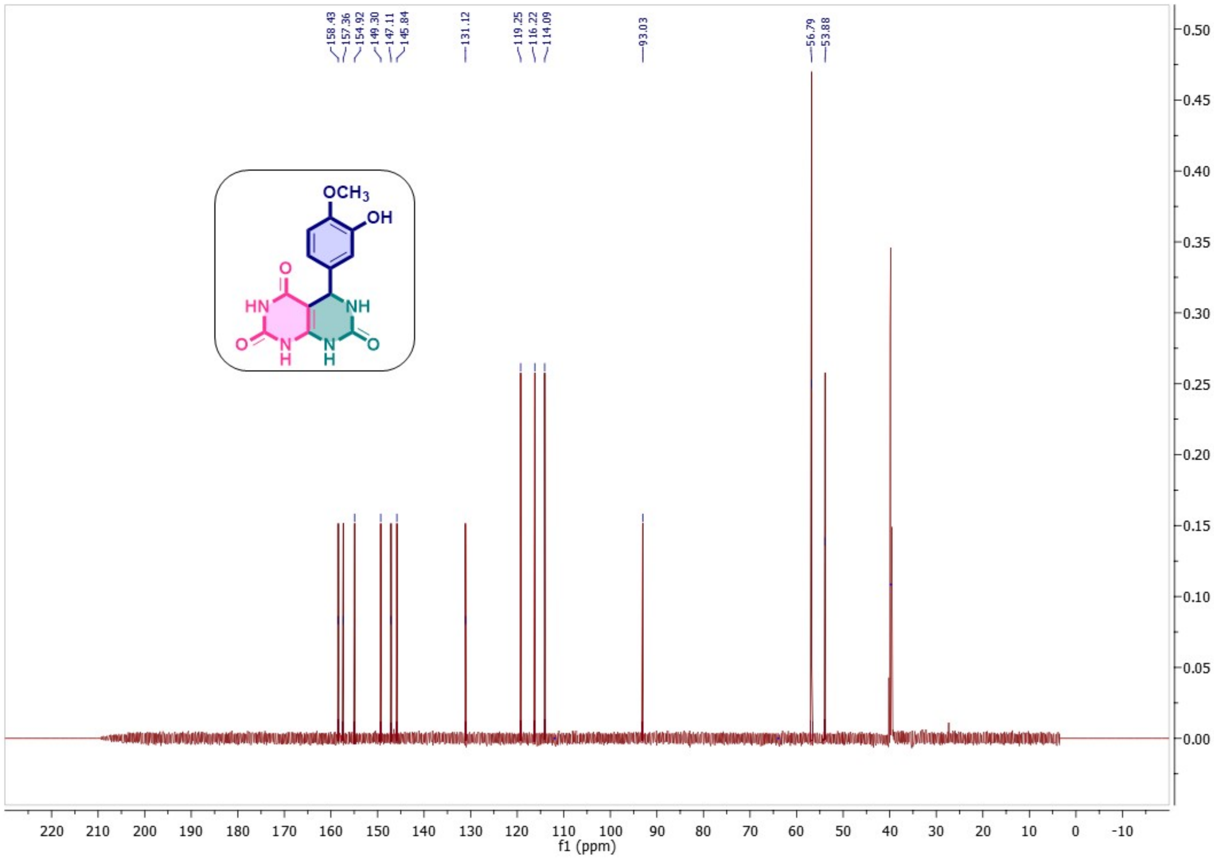

Figure 29: ^{13}C NMR spectrum of compound 4j

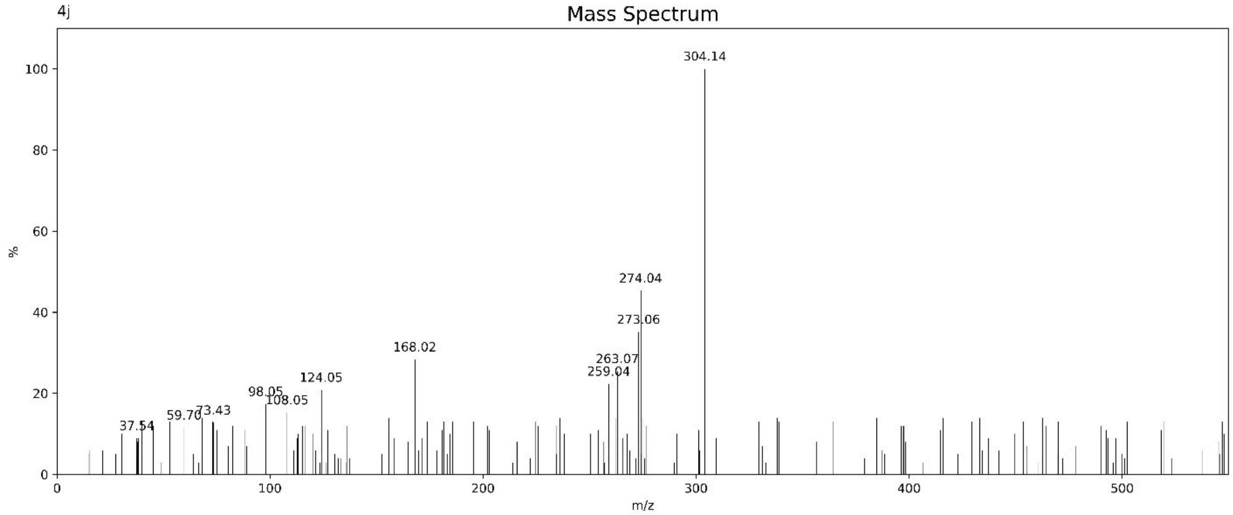

Figure 30: LC-MS spectrum of compound 4j

4k: 5-(4-Hydroxy-3-methoxyphenyl)-5,8-dihydropyrimido[4,5-*d*]pyrimidine-2,4,7(1*H*,3*H*,6*H*)-trione

Off white solid, yield 91%, m.p.- 276-278 ^{o}C, ^{1}H NMR (400 MHz, DMSO-d6) δ 8.60 (s, 1H), 6.98 (s, 1H), 6.88 (s, 1H), 6.64 (d, *J* = 31.7 Hz, 2H), 6.45 (s, 1H), 6.12 (s, 1H), 5.55 (s, 1H), 3.86 – 3.82 (m, 3H), 3.37 (s, 1H). 13C NMR (101 MHz, DMSO-d6) δ 158.43, 157.36, 154.92, 149.30, 148.03, 147.29, 132.08, 119.87, 115.76, 110.65, 93.03, 56.79, 53.88. LC–MS (ESI^{+}): tR = 1.89 min; found = 304.07 Anal. % $C_{13}H_{12}N_4O_5$, C, 51.32; H, 3.98; N, 18.41; O, 26.29.

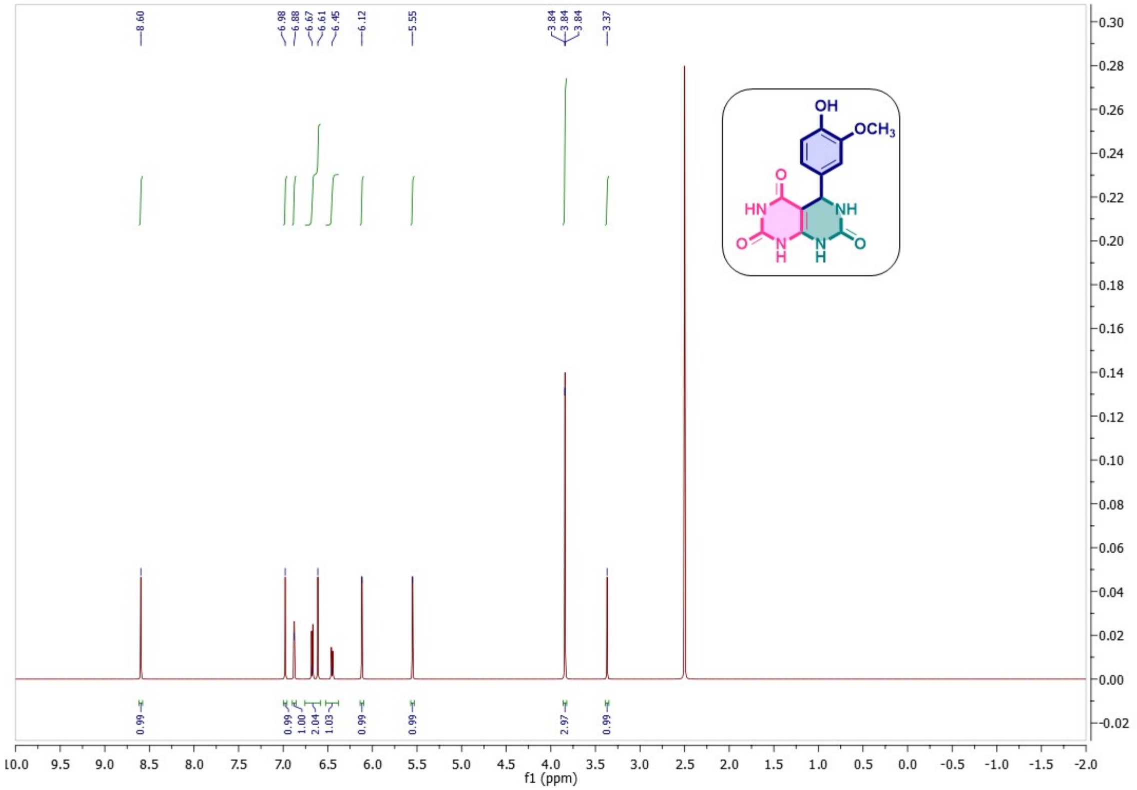

Figure 31: ^{1}H NMR spectrum of compound 4k

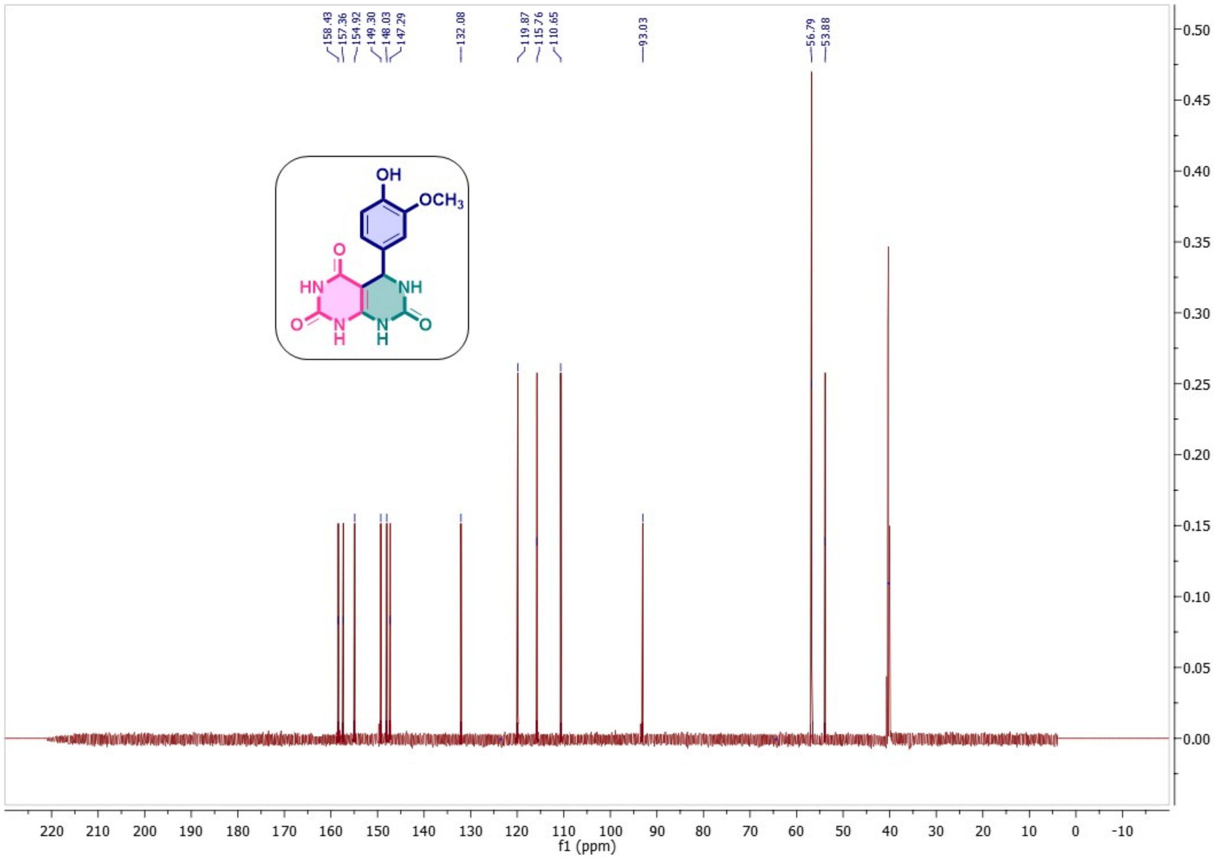

Figure 32: ^{13}C NMR spectrum of compound 4k

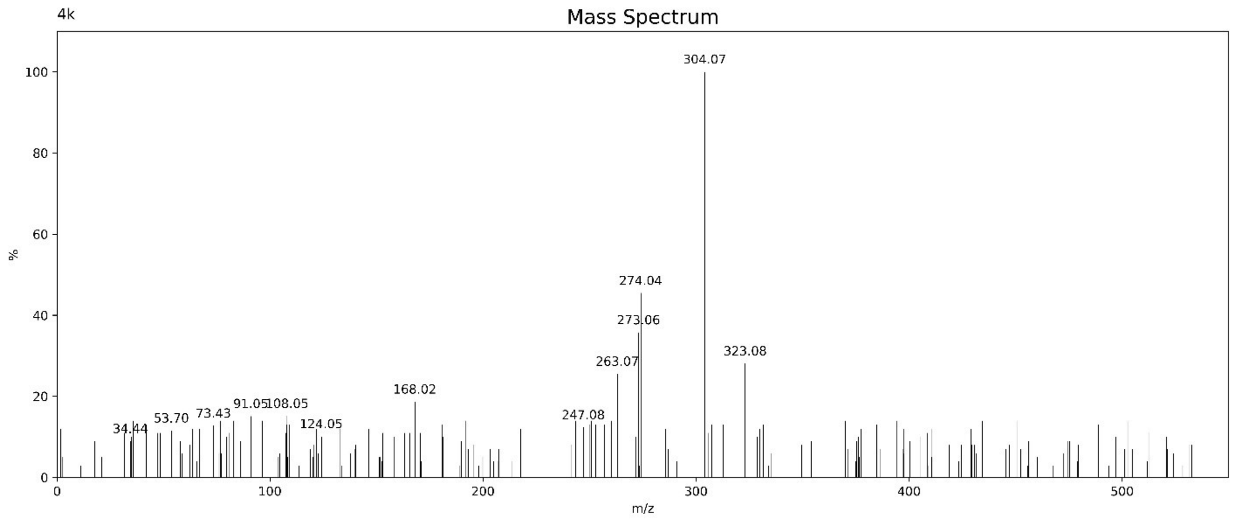

Figure 33: LC-MS spectrum of compound 4k

4l: 5-(4-(Benzyloxy)phenyl)-5,8-dihydropyrimido[4,5-*d*]pyrimidine-2,4,7(1*H*,3*H*,6*H*)-trione

Off white solid, yield 95 %, m.p.- 276-278 °C, ^{1}H NMR (400 MHz, DMSO-d6) δ 7.32 – 7.26 (m, 4H), 7.22 (s, 1H), 7.19 – 7.09 (m, 2H), 7.00 – 6.88 (m, 3H), 6.62 (s, 1H), 6.34 (s, 1H), 6.11 (s, 1H), 5.61 (s, 1H), 5.21 – 5.17 (m, 2H). 13C NMR (101 MHz, DMSO-d6) δ 158.43, 157.37, 157.36, 154.92, 149.30, 137.09, 132.76, 128.77, 128.77, 128.32, 128.32, 128.17, 128.17, 128.16, 114.95, 114.95, 93.03, 70.84, 53.87. LC–MS (ESI^{+}): tR = 1.90 min; found = 364.14 Anal. % $C_{18}H_{14}N_4O_4$, C, 62.63; H, 4.43; N, 15.38; O, 17.56.

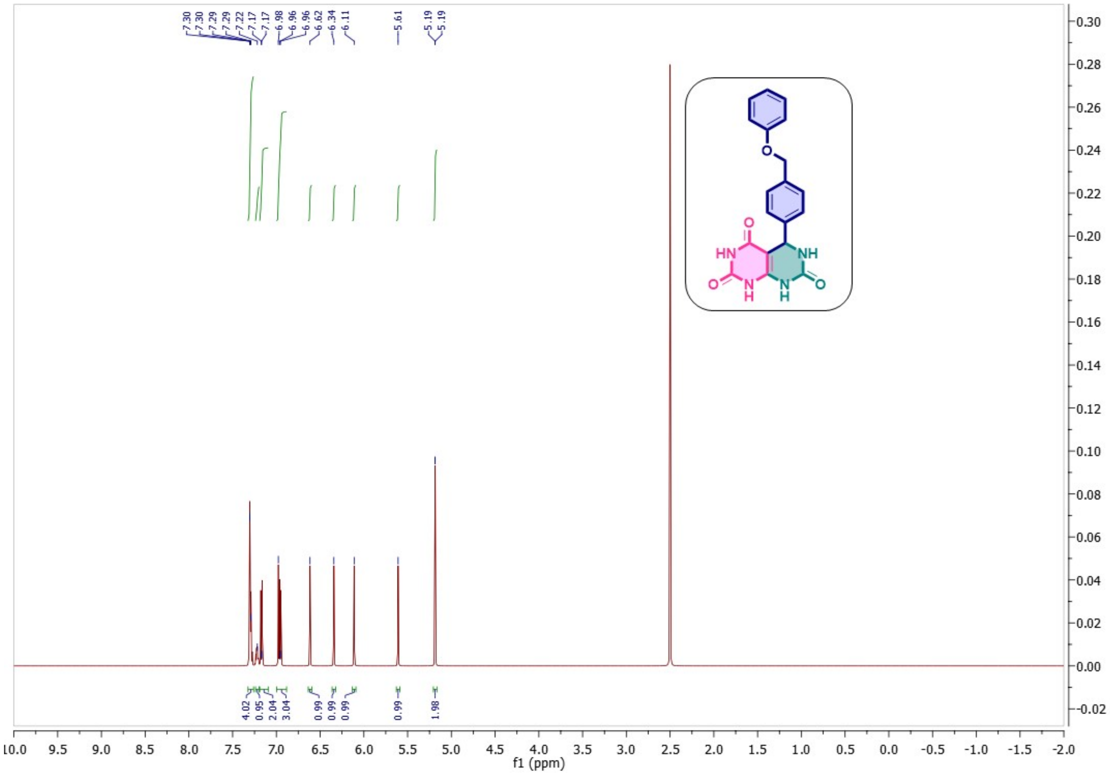

Figure 34: ^{1}H NMR spectrum of compound 4l

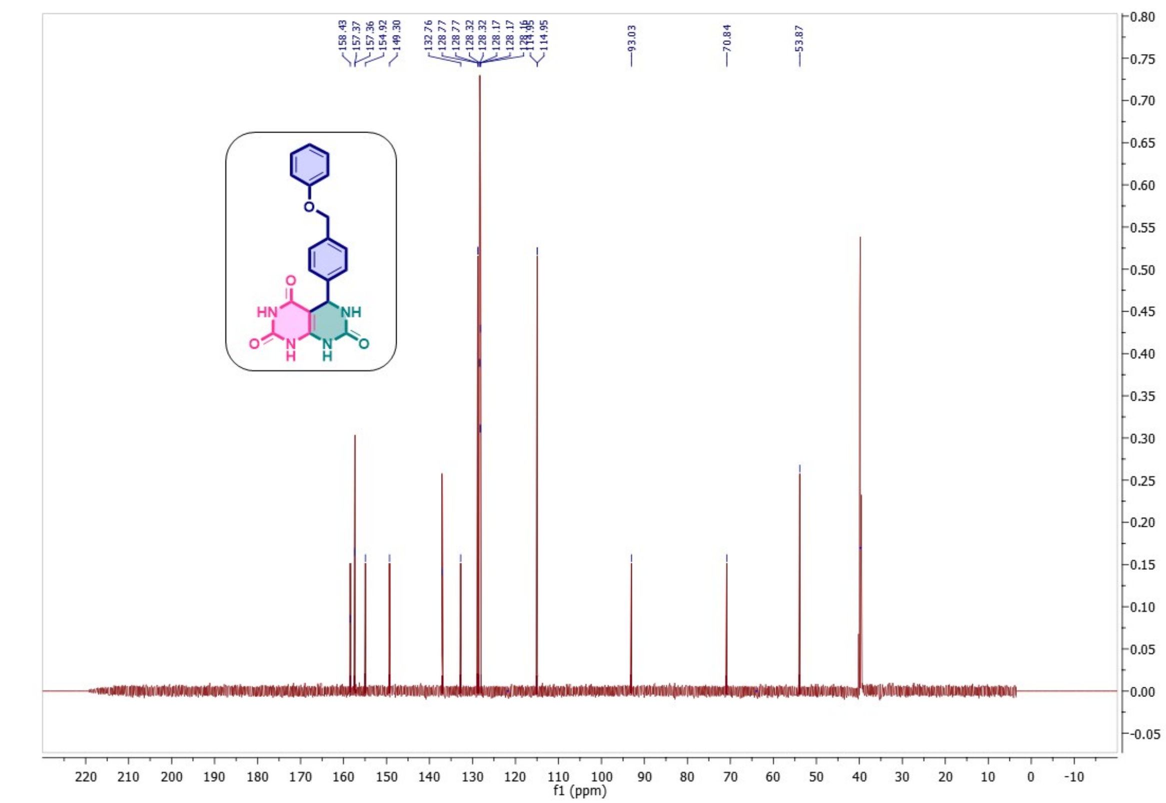

Figure 35: ^{13}C NMR spectrum of compound 4l

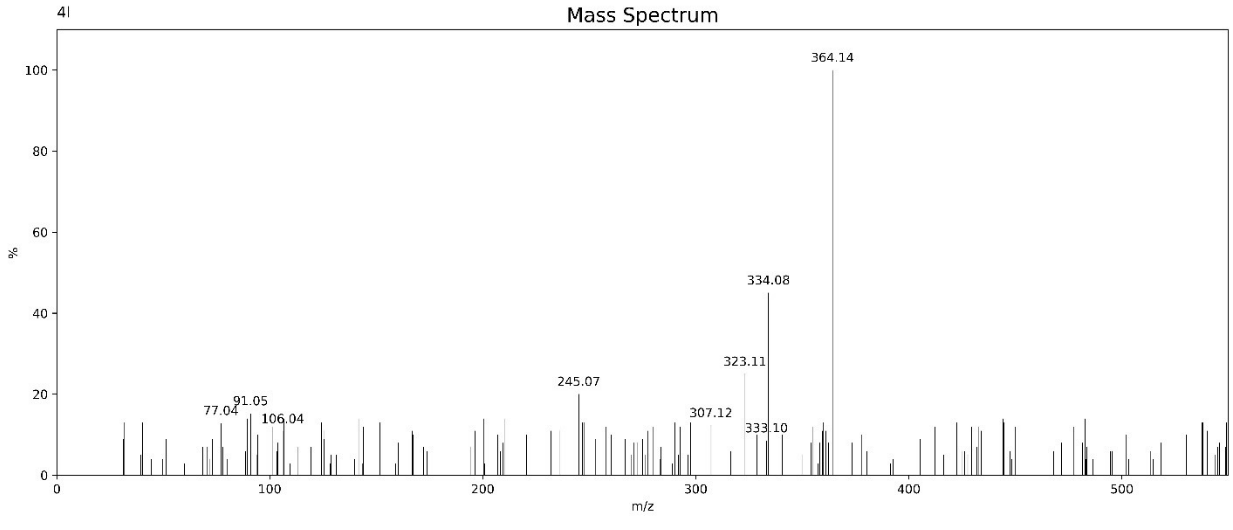

Figure 36: LC-MS spectrum of compound 4l

3. Green chemistry metrix:

The green chemistry parameters for compound **4a** were evaluated to determine the reaction's environmental efficiency and material utilization. The results indicate a high atom economy and low E-factor, reflecting an eco-friendly and efficient synthetic process (**Table 1** and **Table 2**).

Table 1: Calculation for compound 4a:

Compound	Formula wt. (g.mol⁻¹)	Mass used (g)
**Urea**	60.06	0.06006 g
**Barbituric acid**	128.09	0.12809 g
**Benzaldehyde**	106.12	0.10612 g
**Total (reactants)**		0.29427 g

Parameter	Formula	Calculation	Result
**Sum of reactant MWs**	—	60.06 + 128.09 + 106.12	**294.27 g.mol⁻¹**
**Product MW (assumed)**	—	—	**244.27 g.mol⁻¹**
**Atom Economy (AE)**	(M.W. product / Σ M.W. reactants) ×100	(244.27 / 294.27) ×100	**83.01 %**
**Reaction Mass Efficiency (RME)**	(mass of isolated product / Σ mass of reactants) ×100	(0.244 g / 0.29427 g) ×100	**82.92 %**
**Process Mass Intensity (PMI)**	Total mass input / mass of product	(0.29427 g reactants + 3.94 g EtOH + 0.039 g catalyst) / 0.244 g	**17.51**
**E-factor**	(mass of waste / mass of product)	(Σ reactant mass − product mass) / product mass = (0.29427 − 0.244) / 0.244	**0.206**
**Percent Yield (observed)**	(isolated mass / theoretical mass) ×100	(0.244 / 0.24427) ×100	**99.89 %**
**Eco-score**	100 − Σ penalty points (categories as used previously)	Yield penalty ≈ (100 − 99.89)/2 = 0.055 + solvent (1) + catalyst (1) + energy (1) + reagents (1) + workup (0) + safety (0) + waste (0) = 4.055 total penalty	**95.95**

Table 2: Green chemistry metrics and calculated parameters for the synthesis of compound 4a, including atom economy, reaction mass efficiency, PMI, E-factor, and percent yield

Green chemistry evaluation grid:

The green chemistry parameters for compounds **4b–l** were calculated to assess the efficiency and sustainability of the reaction. The results show high atom economy, good reaction mass efficiency, and low E-factor values, indicating that the method is environmentally benign and resource-efficient (**Table 3**).[1][2][3]

Table 3: The green chemistry metric calculation for all synthesized derivatives

Code	Total mass of reactants (mg)	Product MW (mg = 1 mmol theoretical mass)	Actual mass product (mg)	Yield (%)	Mass of waste (mg)	E-factor	AE (%)	RME (%)	PMI
**4b**	341.320	274.30	**244.127**	89	97.193	**0.398**	**80.36**	**71.53**	**17.70**
**4c**	417.418	350.39	**304.839**	87	112.579	**0.369**	**83.98**	**73.04**	**14.41**
**4d**	327.293	260.27	**234.243**	90	93.050	**0.398**	**79.56**	**71.58**	**18.39**
**4e**	354.363	287.34	**252.859**	88	101.504	**0.401**	**81.07**	**71.37**	**17.13**
**4f**	356.291	289.27	**245.880**	85	110.411	**0.449**	**81.18**	**69.01**	**17.62**
**4g**	356.291	289.27	**269.021**	93	87.270	**0.324**	**81.18**	**75.48**	**16.12**
**4h**	345.736	278.71	**250.839**	90	94.897	**0.378**	**80.62**	**72.57**	**17.25**
**4i**	345.736	278.71	**245.265**	88	100.471	**0.410**	**80.62**	**70.97**	**17.63**
**4j**	345.736	278.71	**253.626**	91	92.110	**0.363**	**80.62**	**73.44**	**17.06**
**4k**	357.319	290.30	**269.979**	93	87.340	**0.324**	**81.23**	**75.54**	**16.06**
**4l**	357.319	290.30	**246.755**	85	110.564	**0.448**	**81.23**	**69.06**	**17.58**

4. Docking analysis:

To bridge computational modeling with experimental outcomes, a comprehensive *in silico* workflow was employed, encompassing ligand preparation, protein refinement, molecular docking, dynamics simulations, and ADMET evaluation. Ligand structures were retrieved in SDF format, energy-minimized using the MMFF94 force field in Open Babel, and converted into PDBQT format. The crystal structure of the target protein (PDB ID) was obtained from the Protein Data Bank, followed by removal of bound ligands and water molecules. Polar hydrogens and Kollman charges were introduced, and the refined protein was stored in PDBQT format.[4][5]

Molecular docking was performed using AutoDock Vina integrated in PyRx 0.8 with an exhaustiveness of 8, after defining the grid box dimensions around the active site (____ × ____ × ____ Å^3). Binding poses were ranked by affinity and examined for key interactions using Maestro Visualizer. To probe structural stability, molecular dynamics simulations were conducted in Desmond 2021-4 with the OPLS-2005 force field. Complexes were solvated in the SPC water model within an orthorhombic box, neutralized with 0.15 M Na^+/Cl^-, minimized for 100 ps, and simulated for 100 ns under 300 K and 1.0325 bar. [6]

5. Bioavailability radar plots depicting drug-likeness properties of the ten docked candidates as obtained from SwissADME:

The assessment of drug-likeness was carried out using bioavailability radar plots generated from the SwissADME web tool. These plots offer a rapid and intuitive evaluation of key physicochemical parameters that influence oral bioavailability, including lipophilicity, molecular size, polarity, solubility, saturation, and molecular flexibility. Compounds positioned largely within the optimal range (pink zone) are predicted to exhibit favorable pharmacokinetic behavior, thereby supporting their potential as drug-like candidates (**Figure 37**).

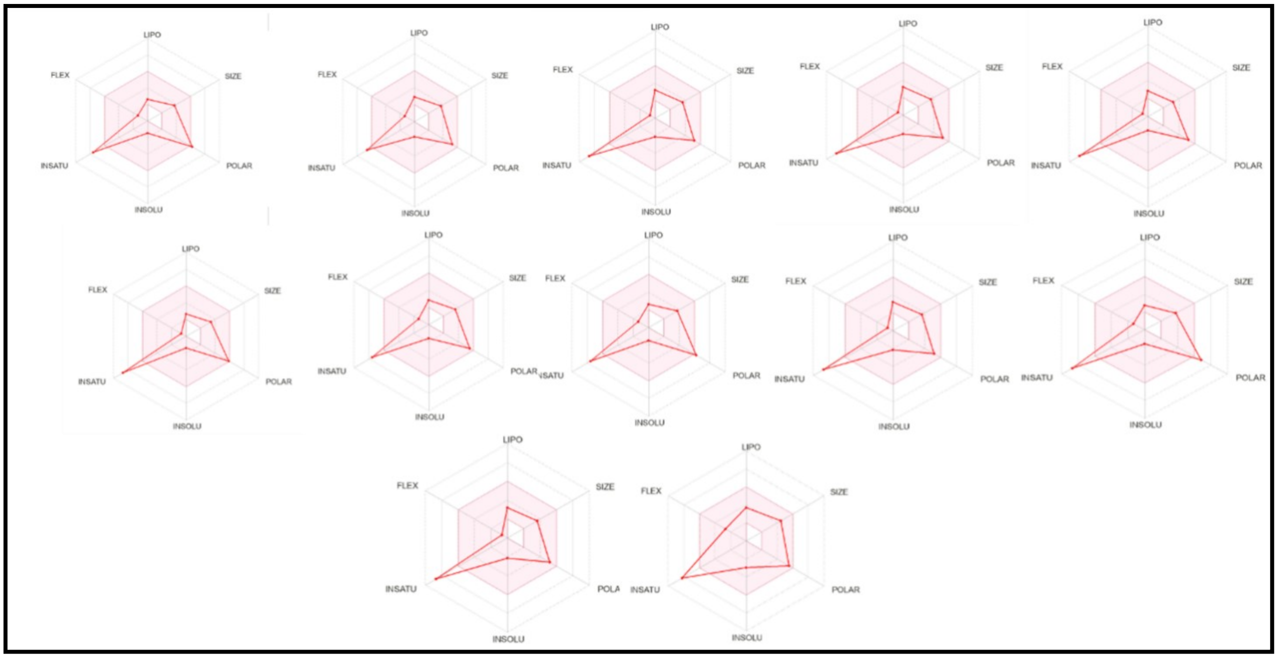

Figure 37: SwissADME [3] radar charts depicting drug-likeness of ten docked candidates across six parameters: LIPO (−7.0 to +5.0), SIZE (<500 Da), POLAR (20–130 Å^2), INSOLU (−6 to 0), INSATU (0.25–10), FLEX (0–9)

6. Antiproliferative activity:

The human neuroblastoma SH-SY5Y cell line was obtained from the National Centre for Cell Science (NCCS, Pune, India) and cultured in Ham's F12 medium (GIBCO, USA) supplemented with 10% heat-inactivated fetal bovine serum (FBS; HiMedia, India) and 1% antibiotic-antimycotic solution (CELL Clone, India). Cells were maintained at 37 °C in a humidified 5% CO_2 atmosphere and used between passages 31–35. Cytotoxicity of the synthesized compounds (**4i-l**) was assessed by the MTT assay following Rai et al. (2020). [7] SH-SY5Y cells (1×10^4/well) were seeded in 96-well plates, allowed to adhere for 24 h, and then treated with varying concentrations of compounds (10, 25, 50, 100 µM) for 72 h. After treatment, MTT solution (5 mg/mL) was added and incubated for 2 h, followed by solubilization of formazan crystals with DMSO for 45 min. Absorbance was recorded at 570 nm using a Bio-Rad microplate reader, and cell viability was expressed as % relative to untreated controls. Cyclophosphamide (CP) served as the reference drug. [8][9]

7. Antibacterial Screening:

Minimal Inhibitory Concentration:

The antimicrobial activity of the synthesized compounds (**4a–l**) was assessed using the broth microdilution method. Stock solutions of each compound were prepared in DMSO at a concentration of 1 mg/mL. Serial dilutions were made to obtain working concentrations ranging from 2–64 µg/mL. The bacterial strains (*B. cereus*, *E. coli*, *L. monocytogenes*, *P. aeruginosa*) were cultured in nutrient broth. For each assay, 100 µL of inoculum (1×10^6 CFU/mL) was added to an equal volume of diluted compound solution in sterile 96-well microplates. Plates were incubated at 37 °C for 24 hours for bacteria and 28 °C for 48 hours for fungi. MIC values were recorded as the lowest concentration at which no visible microbial growth was observed. All experiments were performed in triplicate, and standard antibiotics (ciprofloxacin for bacteria) were used as positive controls, while DMSO served as the negative control. [10-14]

8. References:

[1] Sheldon, R. A. Green chemistry and resource efficiency: towards a green economy. Green Chemistry, 18 (2016) 3180-3183.

[2] Silvanovich, Andre, Gary Bannon, Scott McClain. "The use of E-scores to determine the quality of protein alignments." Regulatory Toxicology and Pharmacology 54.3 (2009): S26-S31.

[3] Riyadi, P. H., Sari, I. D., Kurniasih, R. A., Agustini, T. W., Swastawati, F., Herawati, V. E., & Tanod, W. A. SwissADME predictions of pharmacokinetics and drug-likeness properties of small molecules present in Spirulina platensis. In IOP conference series: Earth and environmental science (Vol. 890, No. 1, p. 012021). IOP Publishing.

[4] Kagami LP, das Neves GM, Timmers LFSM, Caceres RA,Eifler-Lima VL, Geo-Measures: A PyMOL plugin for protein structure ensembles analysis, Computational Biology and Chemistry (2020)

[5] El-Daly, M. M., Bajrai, L. H., Alandijany, T. A., Alsaady, I. M., Gattan, H. S., Alhamdan, M. M., Dwivedi, V.D., & Azhar, E. I. Exploring *Echinacea angustifolia* for anti-viral compounds against Zika virus RNA-dependent RNA polymerase: a computational study. Scientific Reports, 15 (2025), 4060.

[6] Srivastava AK, Srivastava S, Kumar V, Ghosh S, Yadav S, Malik R, Roy P, Prasad R. Identification and mechanistic exploration of structural and conformational dynamics of NF-kB inhibitors: rationale insights from in silico and *in vitro* studies. Journal of Biomolecular Structure and Dynamics. 42 (2024):1485-505.

[7] Srivastava S, Singh Choudhary B, Mehta P, Sukanya, Sharma M, Malik R. Molecular dynamics insights for PI3K-δ inhibition & structure guided identification of novel PI3K-δ inhibitors. Journal of Biomolecular Structure and Dynamics. 37 (2019) 2404-14.

[8] Goyal, J., Jain, P., Jain, V., Banerjee, D., Bhattacharyya, R., Dey, S., & Rai, N. Melamine Exacerbates Neurotoxicity in D-Galactose-Induced Neuronal SH-SY5Y Cells. Journal of Aging Research, 1 (2023), 6635370.

[9] Buranaamnuay, K. The MTT assay application to measure the viability of spermatozoa: A variety of the assay protocols. Open Veterinary Journal, 11 (2021), 251-269.

[10] Nalawade, T. M., Bhat, K. G., Sogi, S. Antimicrobial activity of endodontic medicaments and vehicles using agar well diffusion method on facultative and obligate anaerobes. International Journal of Clinical Pediatric Dentistry, 9 (2016), 335.

[11] Bhargav, H. S., Shastri, S. D., Poornav, S. P., Darshan, K. M., & Nayak, M. M. Measurement of the Zone of Inhibition of an Antibiotic. In 2016 IEEE 6th International Conference on Advanced Computing (IACC) (pp. 409-414). IEEE. (2016)

[12] Gould, J. C., Bowie, J. H. The determination of bacterial sensitivity to antibiotics. Edinburgh Medical Journal, 59 (1952), 178.

[13] Barnard, R. T. The zone of inhibition. Clinical Chemistry, 65 (2019), 819-819.

[14] Acharya, T., Hare, J. Sabouraud agar and other fungal growth media. In Laboratory protocols in fungal biology: Current methods in fungal biology (pp. 69-86). Cham: Springer International Publishing. (2022).
